# Autoregressive Models Applied to Time-Series Data in Veterinary Science

**DOI:** 10.3389/fvets.2020.00604

**Published:** 2020-09-17

**Authors:** Michael P. Ward, Rachel M. Iglesias, Victoria J. Brookes

**Affiliations:** ^1^Sydney School of Veterinary Science, The University of Sydney, Sydney, NSW, Australia; ^2^Australian Government Department of Agriculture, Water and the Environment, Canberra, ACT, Australia; ^3^School of Animal and Veterinary Sciences, Faculty of Science, Charles Sturt University, Wagga Wagga, NSW, Australia; ^4^Graham Centre for Agricultural Innovation, NSW Department of Primary Industries, Charles Sturt University, Wagga Wagga, NSW, Australia

**Keywords:** time-series analysis, veterinary science, methods, animal disease, canine parvovirus

## Abstract

A time-series is any set of *N* time-ordered observations of a process. In veterinary epidemiology, our focus is generally on disease occurrence (the “process”) over time, but animal production, welfare or other traits might also be of interest. A common source of time-series datasets are animal disease monitoring and surveillance systems. Here, we scan the application of methods to analyse time-series data in the peer-reviewed, published literature. Based on this literature scan we focus on autocorrelation and illustrate the recommended steps using ARIMA (Autoregressive Integrated Moving Average Models) methods via analysis of a time-series of canine parvovirus (CPV) events in a pet dog population in Australia, 2009 to 2015. We conclude by identifying the barriers to the application of ARIMA methods in veterinary epidemiology and suggest some possible solutions. In the literature scan the selected 37 studies focused mostly on infectious and parasitic diseases, predominantly for analytical, rather than descriptive or predictive, purposes. Trends and seasonality were investigated, and autocorrelation analyzed, in most studies, most commonly using R software. An approach to analyzing autocorrelation using ARIMA methods was then illustrated using a time-series (week and month units) of CPV events in a pet dog population in Australia, reported to a national companion animal disease surveillance system. This time-series was derived by summing veterinarian reports of confirmed CPV diagnoses. We present data analysis output generated via the R statistical environment, and make this code available for the reader to apply to this or other time-series datasets. We also illustrate prediction of CPV events by rainfall as a covariate. Time-series analysis using ARIMA methods to understand and explore autocorrelation appears to be relatively uncommon in veterinary epidemiology. Some of the reasons might include limited availability of data of sufficient time unit length, lack of familiarity with analytical methods and available software, and how to best use the information generated. We recommend that wherever feasible, such time-series data be made available both for analysis and for methods development.

## Introduction

A time-series is any set of *N* time-ordered observations of a process (1). Within the discipline of epidemiology, our goal is often to understand the underlying processes that generate time-series of disease events. These processes can be explored as part of a time-series analysis, particularly when potential explanatory variables are included as covariates. This can provide insights into disease causation, and thus contribute to the formulation of disease prevention and control programs. However, time-series analysis can also be predictive, with or without covariates. This facilitates the development of forecasting systems to anticipate disease occurrence or detect changes in disease occurrence. Here, we focus on the former goal of understanding disease occurrence.

A key property of time-series is non-independence of values at consecutive time periods. This results in a statistical relationship between values at consecutive time periods and sometimes at different time lags, known as *autocorrelation*. Temporal autocorrelation is a fundamental characteristic of observations recorded over extended periods of time. We can appreciate that daily rainfall data, for example, recorded over a period of months will show autocorrelation: if it rains on a specific day, it is more likely to rain the following day. In addition, rainfall might be more common during certain months, or seasons. Perhaps less obvious is autocorrelation in time-series of disease occurrence. Diseases can be clustered in time due to causes that are autocorrelated (such as climate), due to the methods used to detect disease and the surveillance programs used (for example, certain diagnostic tests only being performed on Mondays, or inspectors at abattoirs working fixed 6-day shifts), and (for infectious diseases) because the number of infected individuals at one time period directly affects the number of infected individuals at a subsequent time period due to disease transmission. Rather than searching for evidence of temporal clustering (2), autocorrelation methods assumes it is present and seek it describe and understand it. Whilst temporal autocorrelation might be expected, often it is subtle.

Autocorrelation makes common statistical approaches inappropriate, and alternative techniques are needed. Time-series analysis invariably begins with descriptive analyses of the dataset under consideration. This consists of separating out (“decomposing”) the time-scale dependent characteristics which make up the observed temporal pattern of disease or event occurrence. Broadly, these patterns are the long term (secular), periodic cyclical (if time-independent), and seasonal trends. The aim of this analysis is to characterize temporal patterns. There are a variety of methods for decomposition, including decomposition based on locally-weighted scatterplot smoothing [“seasonal and trend decomposition using locally weight scatterplot smoothing (loess),” STL]; we demonstrate this method in the context of the CPV events. The process of decomposition, whilst attempting to remove autocorrelation from a time-series, also allows an understanding of the autocorrelation itself and its potential causes.

As part of the process of exploring a time-series, *autoregressive models* can be used to determine how much of the observed time-series can be explained by previous observations in the time-series itself. Characterization of temporal patterns—such as trend and seasonality—can be used to understand potential causes of disease. Autoregressive models to describe the occurrence of events based on prior observations include simple autoregressive (AR) models, autoregressive moving average (ARMA) and autoregressive integrative moving average (ARIMA) models, which differ in the way previous values in the time-series are used to describe future values. AR models are essentially linear regressive models for which each regression term is a time-lagged value (i.e., a value measured at a previous time point—the “lag”) of the same time-series. MA models instead use lagged values of forecast errors, and ARMA models combine both. ARIMA models can also include differencing (i.e., the value at one time point is subtracted from the value at another time point) of the series. Causation can be further investigated by multivariate models. For example, autoregressive models can be extended to include covariates, and in a further extension, information from more than one time-series can be used in vector autoregressive models to forecast future values of each time-series. We demonstrate the way in which visual exploration of autocorrelation function (ACF) and partial autocorrelation function (PACF) plots can provide insights into how to fit a model, and how to select the best model fit for ARMA and ARIMA models.

We begin our discussion of the analysis of time-series data in veterinary epidemiology from our perspective that ARIMA methods are not commonly applied within the discipline. In situations in which methods to analyse time-series data have been applied, we investigate the more commonly used methods and data sources reported via a scan of recent literature. This is motivated by an appraisal of current usage and gaps in the field, rather than a comprehensive, systematic review, to provide the reader with a range of literature in which methods for analysis of times-series data have been used. We then demonstrate the application of autoregressive models using ARIMA methods on a surveillance dataset, and make recommendations to increase the use of such methods in veterinary science.

## Literature Scan

CAB Abstracts Index via Web of Science was searched using TOPIC: (time-series) and TOPIC: (analysis) and TOPIC: (veterinary) during the timespan 1980 to present (31 August 2019), restricted to English language journal articles only. The titles of all articles returned by this search were screened for scope [time-series analysis methods applied to animal (including zoonotic) diseases]. Note that studies in which *time-series data* were reported, but which did not describe the application of *time-series analysis* methods, were excluded.

A template was developed—via discussion between the authors—to extract information from each article (see Supplementary Table 1). Full versions of the subset of articles were then obtained and randomly assigned to one of the three authors.

In total, 60 articles (see Supplementary Table 2) were identified. Of these, five were unavailable for review and 18 were out-of-scope. The latter included articles in which the primary event was a disease in humans only (for example, dengue fever, Crimean-Congo haemorrhagic fever, tick-borne encephalitis, Ross River fever), or the focus was on detection of aberrations within a time-series [for example, (3)]. We applied these exclusions because our aim is to introduce readers to autoregressive models and applications to animal diseases.

Of the remaining 37 articles, publication year ranged from 1990 to 2019 and studies were conducted in 19 different countries (Supplementary Table 2). One study was conducted at the global scale [highly pathogenic avian influenza; (4)]. Data used in these studies were derived from surveillance systems (including internet searches) (14); monitoring systems (11), for example slaughterhouse recording systems; clinical records (6); laboratory records (3); and bespoke research projects (3). These studies were focused mostly on livestock (26). The temporal unit of data collection was most commonly day (17) or month (16), and the median period (years) covered by the datasets analyzed was 10 (IQR 5–16).

The studies identified focused on a wide range of events, but mostly either specific infectious diseases (e.g., rabies) or defined syndromes (e.g., pleurisy and pneumonia).

The purpose of the time-series analysis performed was either analysis (18), description (12), or prediction (7). Studies were considered descriptive if they included only visualization of the time-series or descriptive statistics, whereas those that also included decomposition of the series, or developed models of the time-series, were considered analytical. Those that used the models to predict trends beyond the range of the time-series were considered predictive. Data analyzed was most commonly counts of events. Where data was manipulated before analysis, aggregation to a coarser temporal unit was most common.

Analysis of trends was performed in most (27) studies, mainly using regression models (13). Autoregression was analyzed in the majority of studies (23). In six of these, autocorrelation and partial autocorrelation functions (ACF and PACF; see section An Example of Time-Series Analysis Methods—Canine Parvovirus Reports for Definitions and Methods) were used, and in other studies (10) modeling approaches were used, including autoregressive models. ARMA or ARIMA models were described in 13 of the 23 studies in which autoregression was analyzed. Seasonality was analyzed in 28 studies, however the methods used varied greatly; for example, visual, ACF and PACF, seasonal autoregressive models, automated exponential smoothing state space models, periodograms, and seasonal and trend decomposition STL.

Forecasting was undertaken in 12 studies. The most common (18) software used to analyse time-series data was R.

We observed that the most often cited advantage of using time-series analysis methods was the ability to predict disease occurrence, contributing to early warning and therefore disease prevention. Some of the barriers discussed include the scarcity of long-term, computerized, automatically collected, and publicly available data; identifying outbreak or disease-free baselines; event data sparseness (excessive zeros); data aggregation (temporal scale); time gaps in the data; lack of constant population at-risk; and model validation.

In summary, in this literature scan, time-series analysis methods in veterinary science were mostly focused on infectious and parasitic diseases, analyzed by decomposing and modeling the time-series. This approach most often involves investigation of trends and seasonality, and analysis of autocorrelation, usually aided by the use of R software. Based on this, we next illustrate methods that can be used to investigate and analyse trends, seasonality and autocorrelation in veterinary science by presenting a step-by-step guide to analysis of a canine parvovirus time-series using R.

We focus on ARIMA methods because beyond a description of the trend and seasonality of time-series data, ARIMA models are an accessible method to describe autocorrelations within data and assess the influence of covariates such as climate variables. These methods can be considered a foundation in autoregressive methods for time-series analysis. Other methods—such as aberration detection algorithms, stochastic modeling approaches and machine-learning methods—can then be investigated for applications requiring long-term prediction (5–7).

## An Example of Time-Series Analysis Methods—Canine Parvovirus Reports

Prior to embarking on autoregressive modeling, we need to consider when it is appropriate to apply these methods—and when it is not. For such analysis, a dataset of sufficient length and completeness needs to be available. Without sufficient data, it is difficult to identify trends and patterns, to build models, and determine statistical significance. In veterinary science, data generated by monitoring and surveillance systems are often analyzed by autoregressive modeling (see section Literature Scan). However, missing data can be an issue (see section Results of Analyzing a Time-series of Canine Parvovirus Reports), as can data gaps in the time-series caused by temporary interruptions to data collection. Assuming a stable population at-risk simplifies analysis and interpretation of results, but such assumptions need to be plausible. Other more general epidemiological issues—such as selection, ascertainment and measurement bias—also are applicable to autoregressive modeling and need to be considered.

Here we describe an analysis using autoregressive methods as an example that readers can use to guide their own analyses (8). The data and R code used for the analysis are available at https://zenodo.org/record/3738684#.X1HOYNZuLIU (accessed 04/09/2020).

Our time-series analysis begins with a description of the data, including the source, results of initial data checking and any manipulation required to make it suitable for time-series analysis. The time-series is then plotted, and secular and seasonal trends are assessed using decomposition then linear regression. Before fitting an autoregressive model, the series is assessed for stationarity using graphical and statistical methods. Stationarity is a key requirement to fit models to time-series data. A series is considered stationary if it is not changing systematically over time. A method for inducing stationarity—differencing—is also explained and demonstrated. We then fit a number of ARIMA models and use these to forecast disease cases beyond the range of the dataset. Finally, we investigate the influence of a covariate (rainfall) on the time-series and give a brief example of how cross-correlation and vector autoregressive models can be used to investigate relationships in time-series. We present the analysis of the example dataset in a stepwise guide to assist the reader to replicate the approach on this or other, similar datasets.

We have used the R statistical environment (9) for all analysis described. For readers not familiar with this platform, introductory courses and tutorials are widely available online and we recommend spending some time familiarizing yourself with the program before attempting this analysis. The code provided in the Zenodo repository will work if you have R correctly installed and operating on your computer and have installed the packages listed below.

The following packages for data visualization, manipulation and analysis of time-series data used in this analysis: ggplot2 (10), plyr (11), dplyr (12), lubridate (13), tseries (14), vars (15, 16), and forecast (17, 18).

To align readers to the associated R code, the corresponding “chunk” (C) in the code (https://zenodo.org/record/3738684#.X1HOYNZuLIU, accessed 04/09/2020) is included in the methods below. Chunks C1-C3 initiate and load the required packages.

Here, we present a series of six steps to guide the reader in applying time-series analysis to the example dataset.

### Step 1: Describing the Data

This worked example uses data from the Disease Watchdog system, in operation since 2010 in Australia and initiated to collect information on infectious diseases of dogs and cats in Australia (19–22). By 2015, nearly 25,000 disease cases and 19,000 reports had been submitted. The system was deactivated in early 2017.

Veterinarians and veterinary clinic staff were the contributors of data within this system. Besides disease diagnoses and their date of occurrence and postcode of residence, a range of other patient data was also collected, including age, sex, neuter status, breed, diagnostic method, and vaccination status. To encourage timely reporting, data was used to produce near-real time disease maps which veterinarians accessed to educate their clients (19). In this example, canine parvovirus (CPV) is used as the event of interest. CPV is a highly contagious disease of dogs and an important cause of morbidity and mortality in young dogs (23). It has a worldwide distribution and occurs as endemic disease or as local outbreaks.

Records of all CPV cases reported Australia-wide between October 2009 and November 2015 were extracted from the Disease Watchdog database. For analysis, cases which were reported to have been vaccinated at any time were excluded. Furthermore, only those cases in which the diagnosis of parvovirus had been confirmed by diagnostic testing were included. To illustrate approaches to analyzing time-series data, we applied these methods to events only, where an event consists of one or more cases reported by the same veterinarian with the same date of occurrence. We also restricted analysis to events reported from the state of New South Wales.

The dataset was loaded (C4) and checked for duplicated or missing data (C5). The number of events, and minimum and maximum dates of occurrence were reported (C6). The number of parvovirus events were then aggregated by week and by month (based on the reported date of occurrence) to create two time-series datasets (weekly and monthly) for subsequent analyses (C7−9).

### Step 2: Visualization

**Figure 1:**
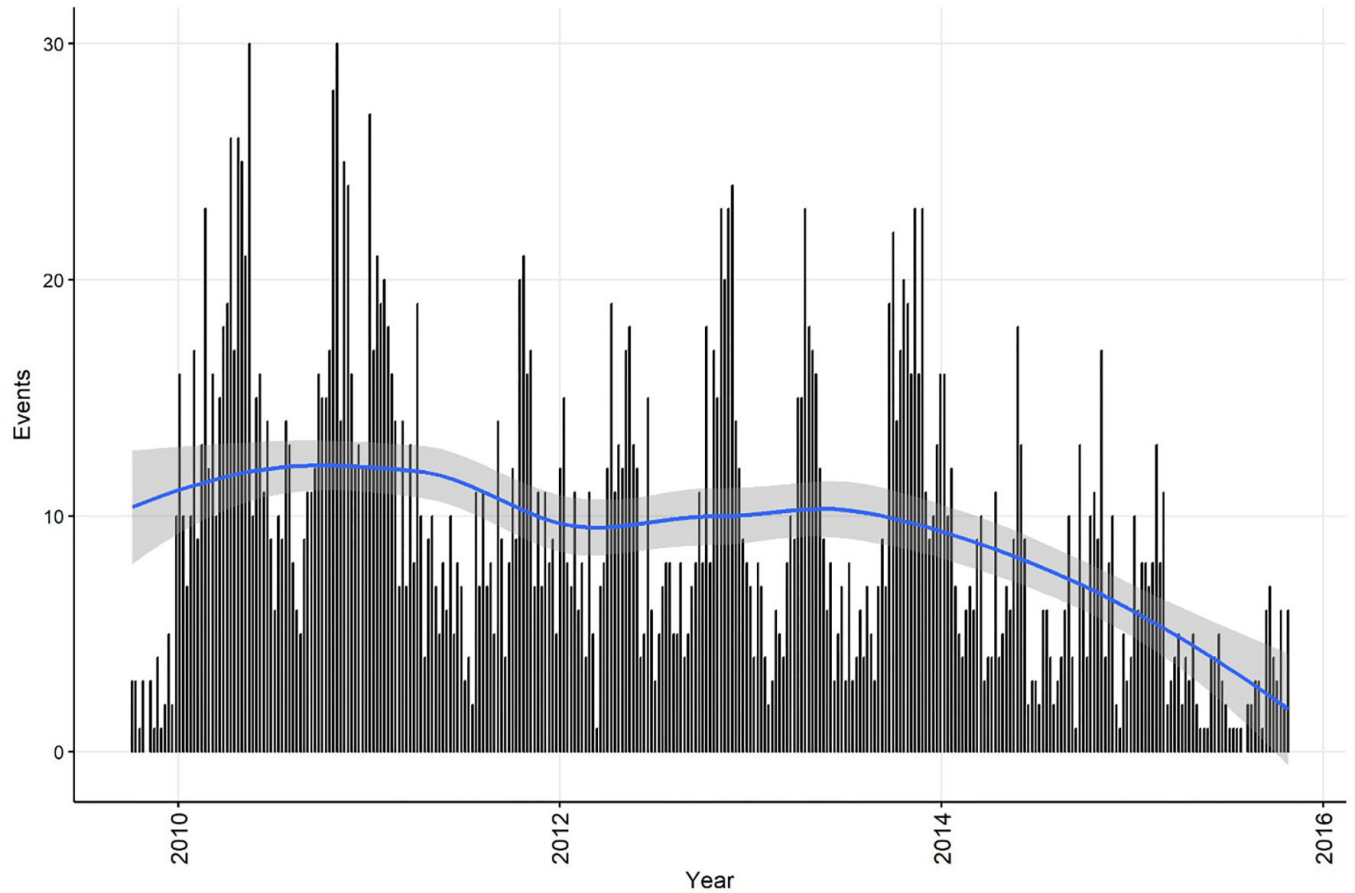
Confirmed canine parvovirus events/week reported from New South Wales in a surveillance system in Australia, 2009–2015. Blue line, loess smoothed curve of events/week with 95% CI (gray).

**Figure 2:**
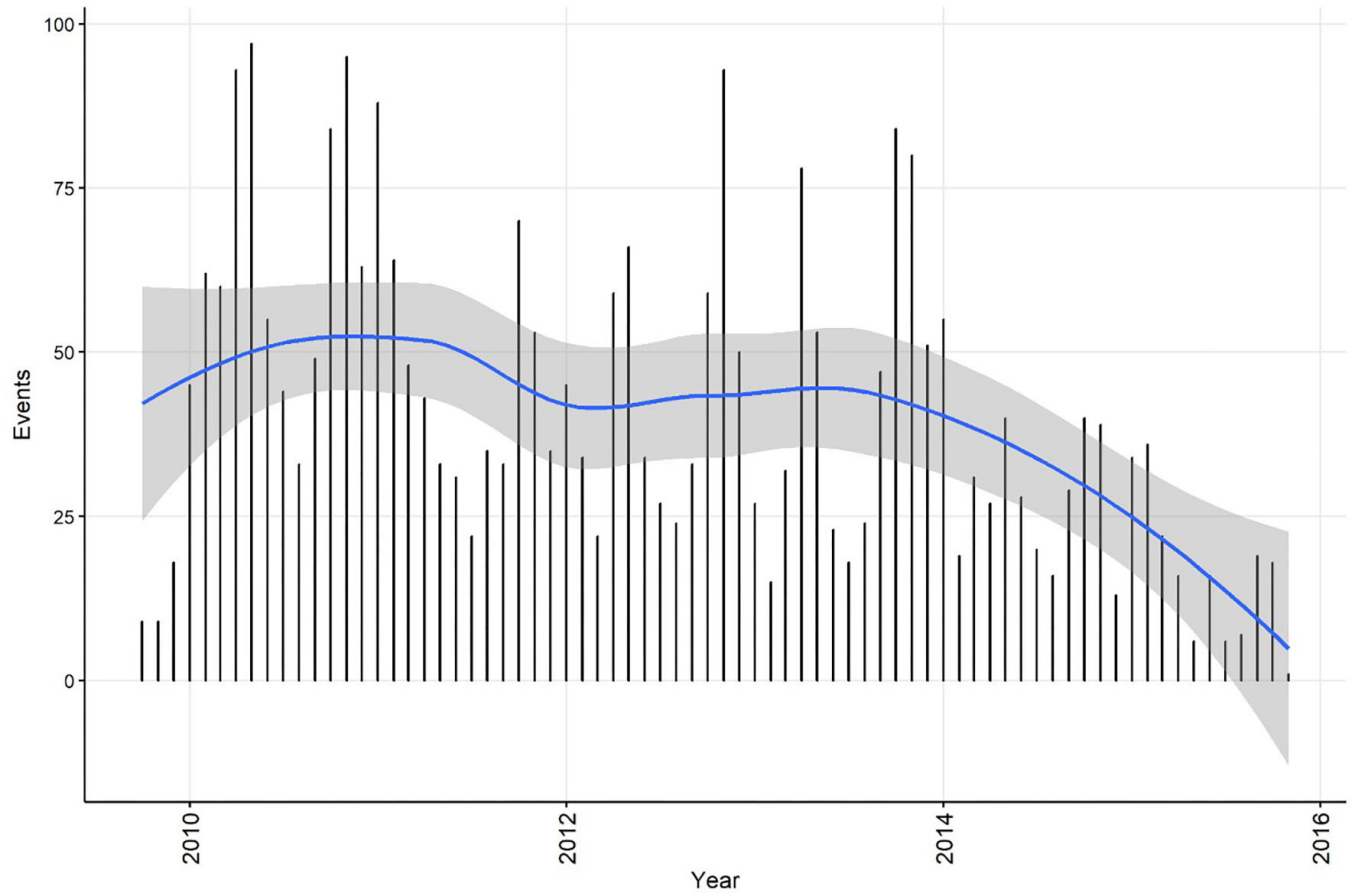
Confirmed canine parvovirus events/month reported from New South Wales in a surveillance system in Australia, 2009–2015. Blue line, loess smoothed curve of events/month with 95% CI (gray).

Summary information on CPV events was calculated for the time series at both the weekly and monthly aggregation, and each dataset was plotted with a smoothed curve of events overlaid to visually assess trend (C11). The smoothing process in R is achieved by loess regression (see section Step 4 for a technical explanation of this method). This is exploratory analysis that can be used to inform further analytical approaches. Smoothed curves for both events/week (Figure 1) and events/month (Figure 2) demonstrate a decreasing trend over time, with the frequency of events being relatively stable during the period 2010 to 2013. If the aim of the analysis was to investigate risk factors for the pattern of events observed, this might suggest that the time-series can be truncated to the period 2010 to 2013, inclusive. If changes in CPV surveillance are of interest, further analysis might include the entire time-series. In addition, these initial plots and smoothed curves can inform the temporal scale of analysis. Visual assessment of Figures 1 and 2 suggests that monthly aggregation of events is sufficient to preserve the patterns present in the data. However, if the aim of analysis is to identify covariates associated with these patterns, the temporal units used to collect covariate data would also need to be considered.

### Step 3: Linear Regression

After conversion of the events series to a computer-recognized “time-series object” (C12), linear regression analysis was used to further explore and quantify secular and seasonal trends (C13). The outcome was the number of events per week (or per month) and the predictors were time in weeks (or months) to assess trend, and week (or month) of the year to assess seasonality. Linear regression is used to confirm impressions from time-series plots and smoothed curves (step 2), to test the statistical significance and to quantify these trends. Identifying such trends is a major component of analysis of time-series data, and can lead to hypothesis-generation regarding potential causes of such patterns.

### Step 4: Decomposition

The time-series were then decomposed to separately visualize temporal components including trend and seasonality and the remainder component (also known as “random” or “white noise”). Again, such visualization facilitates the identification and characterization of patterns and potentially what might be causing such patterns. For example, the trend or seasonal pattern might dominate. Alternatively, removing trend and season might still result in the remainder time-series showing a discernable pattern. This suggests greater complexity in the time-series (or the incorrect choice of window size to calculate trend and seasonal components).

Two methods were used: moving averages and “seasonal and trend decomposition using loess” (STL; C14). Both are additive models of the form *Y[t]* = *T[t]* + *S[t]* + *e[t]* in which *Y[t]* is the model output at time *t, T[t]* is the trend component at time *t* (which includes cyclical and longer trend patterns, the “trend-cycle” component), *S[t]* is the seasonal component at time *t* and *e[t]* is the remainder (or residual i.e., what remains in the time-series after removing seasonal and trend components) at time *t*. If the variance of the trend or seasonal components of the time-series is not constant throughout the time-series, a multiplicative decomposition is likely to be more appropriate than an additive model.

In moving averages the trend component is determined using a moving average window of an appropriate width. This trend component is then subtracted from the original values, and the data grouped by the seasonal element and averaged for each season. The seasonal component is determined by subtracting the average of the seasonal averages from each seasonal average.

A challenge is the choice of an appropriate moving average width. A default width of three time units can be chosen, meaning that for every observation in the time-series, the observation immediately preceding and immediately following that observation is used to calculate an average value. If data within a time-series have been collected with a known periodicity (for example, observation of disease conditions at an abattoir collected every Monday and Tuesday), this could also be used to inform the moving average width.

The STL method is an iterative process that recalculates the seasonal and trend components by a loess smoothing procedure that initially fits a low-order polynomial to the data. A robustness weighting is calculated for each time point between each iteration, and incorporated into the smoothing procedure in the next iteration, which also uses the trend component from the previous iteration (24).

### Step 5: Fitting Autoregressive Models

Once the time-series has been explored using the methods above, we use the information gained from these analyses to select and fit an ARIMA model. For demonstration and due to the findings in these exploratory analyses, seasonal autoregressive models with an ARIMA structure were then fitted to the time-series. Autoregression is the relationship between values in a time-series and values in that same time-series measured previously in time (the lag). For example, an autoregressive model of lag 1 describes the relationship between observations and their value in the preceding time unit. The Auto Regressive (AR) terms refer to the number of lagged values in the model. In the non-seasonal part of the model, the order of lagged values is termed “*p*,” and in the seasonal part of the model the order of lagged values is termed “*P*.” Moving Average (MA) terms—not to be confused with the calculation of a moving average in series decomposition—refer to the number of lagged errors in the model. It is essentially the relationship between current and lagged errors in the time-series. In the non-seasonal part of the model, the order of lagged errors is termed “*q*,” and in the seasonal part of the model the order of lagged errors is termed “*Q*.” Integration (*I*) terms refer to the number of differences used to make the time-series stationary. In the non-seasonal part of the model, the order of differences is termed “*d*,” and in the seasonal part of the model the order of differences is termed “*D*.” The overall structure of the model can be written as *(p, d, q) (P, D, Q) m*, in which *m* refers to the number of time-series observations in a seasonal cycle.

The time-series (weekly and monthly reported CPV events) were assessed for stationarity to determine the orders for *d* and *D* to use in the ARIMA model. Initially, an automated function in R was used to determine if differencing was required for both the non-seasonal components (*d*) and seasonal components (*D*) of the ARIMA model using a sequence of unit root tests (KPSS test as default, C16). Stationarity was then further assessed using visualization of time-series plots, auto-correlation function (ACF) plots, and statistical tests (C17−18). Statistical tests included the Ljung-Box test, the Augmented-Dickey Fuller (ADF) test and the Kwiatowski-Phillips-Schmidt (KPSS) test. In the case of a non-stationary time-series, the time-series was first-differenced and assessed again for stationarity. The objective of applying this range of methods is to ensure that any need for differencing—either non-seasonal or seasonal—is identified. Some methods (particularly statistical tests) might not suggest the need for differencing in specific datasets, so a conservative approach is to apply several methods.

ACF and PACF plots were also used to assess the moving average (MA; q, Q) and autoregressive (AR; p, P) non-seasonal and seasonal components of the weekly and monthly ARIMA models following differencing (C19). The ACF plot allows us to visualize the correlation between values in the series and values lagged at a certain number of time points previously, whereas the PACF plot shows the correlation between values in the series and those at a given lag after removing the effect of values at intervening lags. ACF plots can indicate the moving average order *q* to include in an ARIMA model i.e., the lag at which autocorrelation becomes statistically non-significant. Similarly, the PACF plot can inform on the autoregressive order *p* to include. These functions can also be used to inform on seasonal moving average and autoregressive orders, respectively. We give a practical demonstration of how to interpret ACF and PACF for the purposes of ARIMA model parameterization in section An Example of Time-Series Analysis Methods—Canine Parvovirus Reports, using the time-series of CPV events.

Auto-fitting was used to select a starting model (C20−21). Further models were constructed that were simpler (lower parameter terms than the auto-fitted models) but still within the parameter terms for *(p, d, q) (P, D, Q)* that were estimated during exploratory analysis (C22). The models with the lowest Akaike Information Criterion (AIC) estimates were selected. Model fit was assessed by visualization of predicted time-series relative to observed time-series, and examination of residuals for stationarity (time-series plot, ACF plot, Ljung-Box test) and normality. Because of the auto-fitting algorithms used to identify candidate ARIMA models, it is important to also visualize model(s) selected to ensure these make logical sense and have a biological explanation. Once a final model has been selected, it can be used to predict events for a specified time period beyond the range of the time-series. A predictive model can form the basis of a forecasting system, in which timely anticipation of disease events allows response strategies to be implemented. There are examples of forecasting in veterinary science using time-series analysis (see section Literature Scan). We demonstrate the use and interpretation of these methods in the context of the CPV data below.

### Step 6: Multivariate Analysis

To illustrate multivariate time-series analysis methods, a corresponding time-series of rainfall was created. The center of the postcodes in NSW from which CPV was reported during the study period was identified. This was achieved by joining case and event data to a polygon shapefile of NSW postcodes (ArcGIS v. 10.5. ESRI). We then identified the central feature (Spatial Analyst. ESRI), postcode 2850. From this postcode, a Bureau of Meteorology weather recording station was identified [Mudgee (062021), 32.58°S, 149.58°E] and daily rainfall data during the period 1 January 2009 to 31 December 2015 was extracted ^1^. Any missing data in the time-series were supplemented by accessing data from the closest weather recording station [Mudgee Airport AWS (062101)]. The rainfall time-series was then aggregated to a monthly time unit to produce a time-series of total monthly rainfall. Dependent on the data, other metrics might be more appropriate, such as monthly median daily temperature or total monthly degree-days.

Covariate time-series datasets are often derived secondarily to the primary time-series of interest (often disease data in veterinary science). Besides climate (including rainfall, temperature and humidity), time-series data might be available on economic indicators, landscape and environmental variables and demographics. For analysis, data need to have the same temporal scale and duration (including time lags) as the primary time-series of interest, and should also broadly match the spatial extent (i.e., when covariates are used, they should be derived from the same area as the outcome of interest, rather than from a larger or a different area).

The presence of substantial data gaps in the series (other than randomly distributed missing data as in our CPV–rainfall example) can render such series unusable if it is not possible to impute data.

The rainfall data were prepared, described and decomposed to assess temporal trends (C23). Quantitative assessments further investigated the trend, seasonality and need for differencing (C24). An automated function was used to fit a dynamic model (ARIMA with rainfall as a predictor) to the CPV and rainfall time-series (C25). Model fit was assessed by visualization of the predicted time-series relative to the observed time-series, and examination of residuals for stationarity (time-series plot, ACF plot, Ljung-Box test) and normality.

Finally, a vector autoregessive model was fit to the CPV and rainfall time-series following examination of a cross-correlation plot between the CPV and rainfall time-series (C26−28). These models assume that a bi-directional relationship (“feedback”) between the variables is possible. Whilst this might be a useful premise in the context of time-series of disease in different populations (for example, “who infects whom?”), in the context of this dataset this is implausible (CPV events cannot cause rainfall). However, we include the code for demonstration purposes.

## Results of Analyzing a Time-Series of Canine Parvovirus Reports

### Step 1: Data Description

Between 2009 and 2015, a total of 24,602 cases and 19,048 events were reported in the Disease Watchdog system. Of these, 20,182 and 15,499 respectively were dog cases and events. During this time period, there were a total of 7,933 CPV cases and 5,837 CPV events reported.

Following application of selection criteria (diagnostic method, nil vaccination history), a total of 2,987 events (3,584 cases) remained for analysis (1.2 cases per event). The earliest and latest reporting dates were 6 October 2009 and 1 November 2015, respectively. The duration of the time-series dataset was 2,218 days, 315 complete weeks and 74 complete months. The median (range) number of cases reported per week was 9 (1–45), and the median (range) number of events reported per week was 8 (1–30).

### Step 2: Visualization

The temporal distributions of weekly and monthly events are shown in Figures 1,2, respectively. A decrease in reported events during the period was apparent in both time-series (indicated by the blue line generated by a loess smoothing function). There were no gaps in the time-series of events.

### Step 3: Linear Regression

Linear regression analysis indicated that the decrease in events/week was 1.22 each year (95% CI 0.87−1.57 events/week each year). Statistically significant (*P* ≤ 0.05) decreases in the number of events were observed in weeks 11, 24, 26−29, 31−35, and 38. Linear regression analysis indicated that the decrease was 5.76 monthly events/year (95% CI 3.02−8.50 events/year). Statistically significant (*P* ≤ 0.1) decreases in the number of events were observed in July and August (winter season).

### Step 4: Decomposition

**Figure 3:**
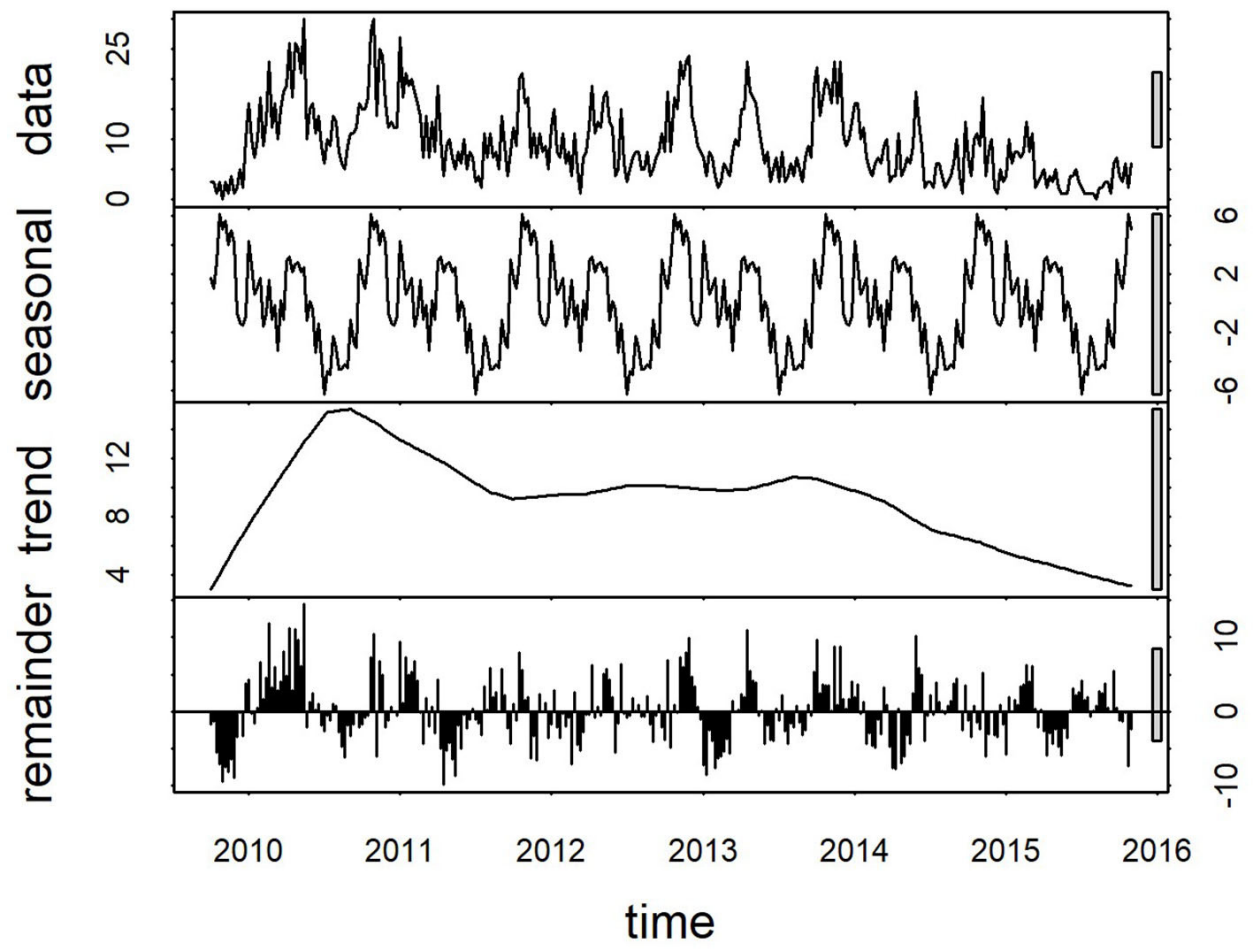
Components of a time-series of weekly confirmed canine parvovirus events reported from New South Wales in a surveillance system in Australia, 2009–2015. Y-axis units, parvovirus events/week. Gray bars on right y-axis indicate the equivalent magnitude of variation of each component (“trend,” “seasonal,” “remainder”) relative to the “data” series, which demonstrates that most variation in the series is in the “remainder” component.

**Figure 4:**
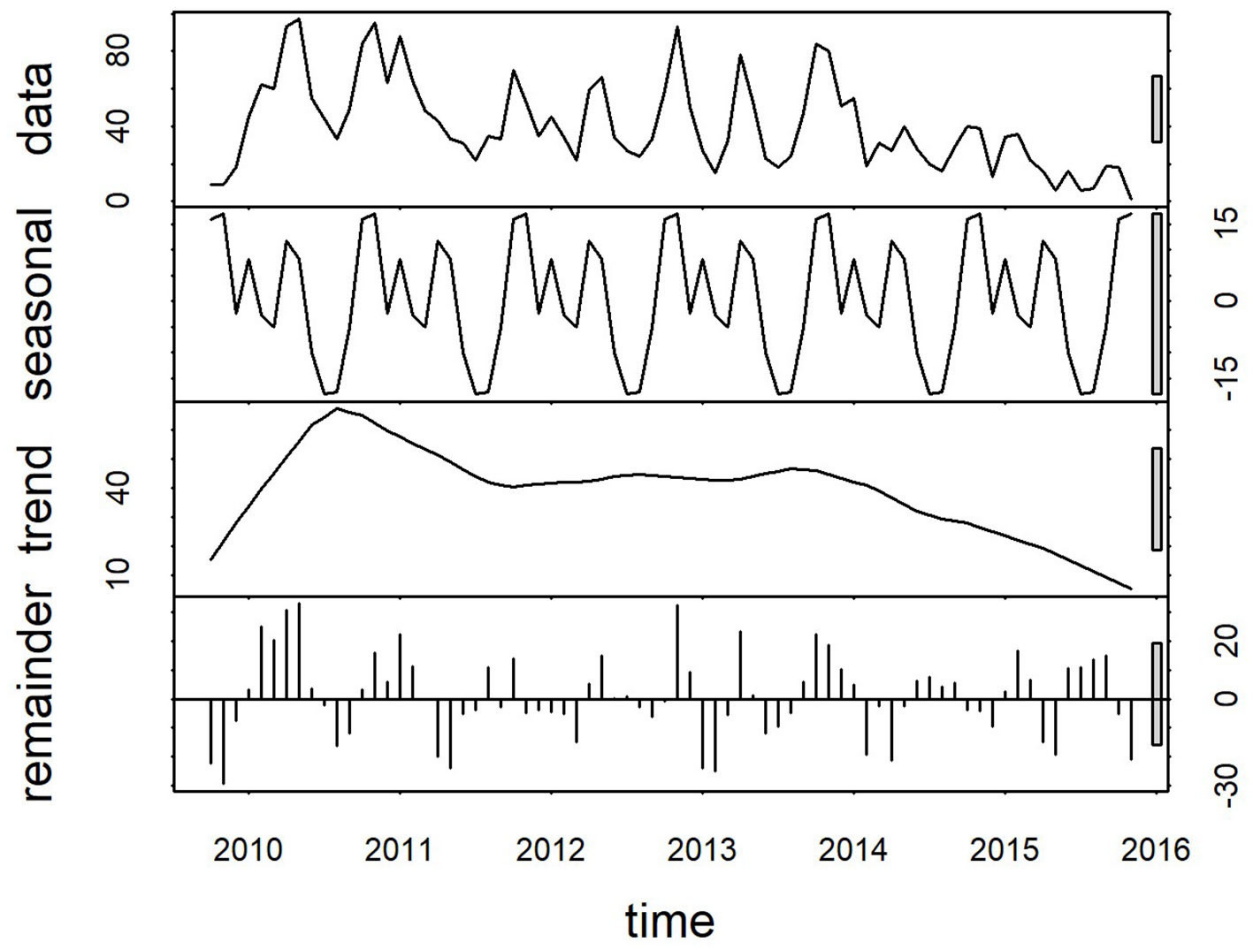
Components of a time-series of monthly confirmed canine parvovirus events reported from New South Wales in a surveillance system in Australia, 2009–2015. Y-axis units, parvovirus events/month. Gray bars on right y-axis indicate the equivalent magnitude of variation of each component (“trend,” “seasonal,” “remainder”) relative to the “data” series, which demonstrates that most variation in the series is in the “remainder”, and “trend” components.

**Figure 5:**
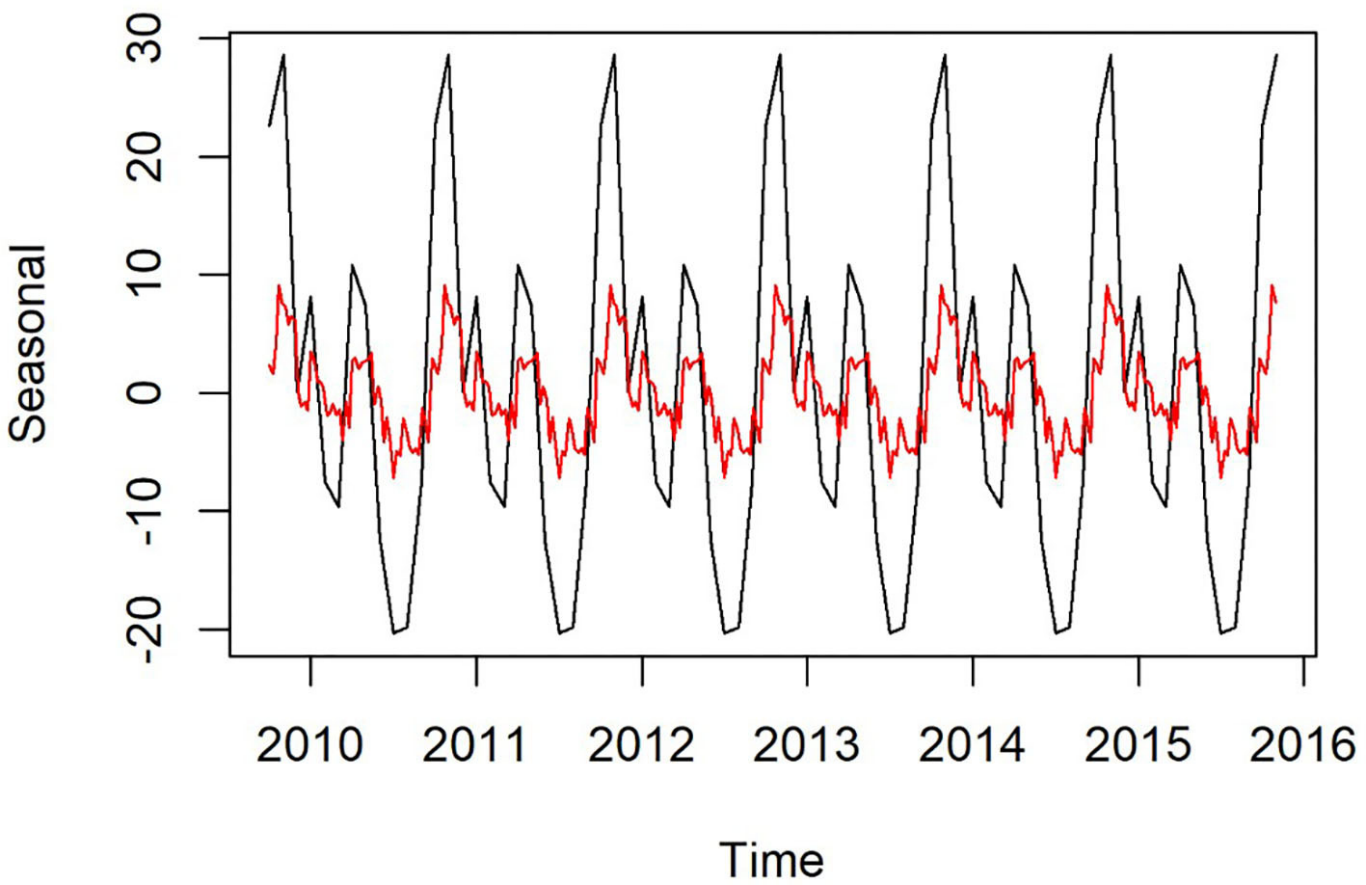
Seasonal component of time-series of weekly **(red)** and monthly **(black)** confirmed canine parvovirus events reported from New South Wales in a surveillance system in Australia, 2009–2015.

Plots of the decomposed time-series are shown in Figures 3,4. The trend lines were consistent with Figure 2, and seasonal cycles were apparent. The weekly and monthly seasonal cycles were overlaid in Figure 5 and illustrated that whilst the patterns were consistent with the regression analyses and with each other, monthly seasonality had a simpler, less variable pattern than weekly seasonality. Both weekly and monthly remainder components appeared to oscillate symmetrically around zero.

### Step 5: Autoregressive Models

**Figure 6:**
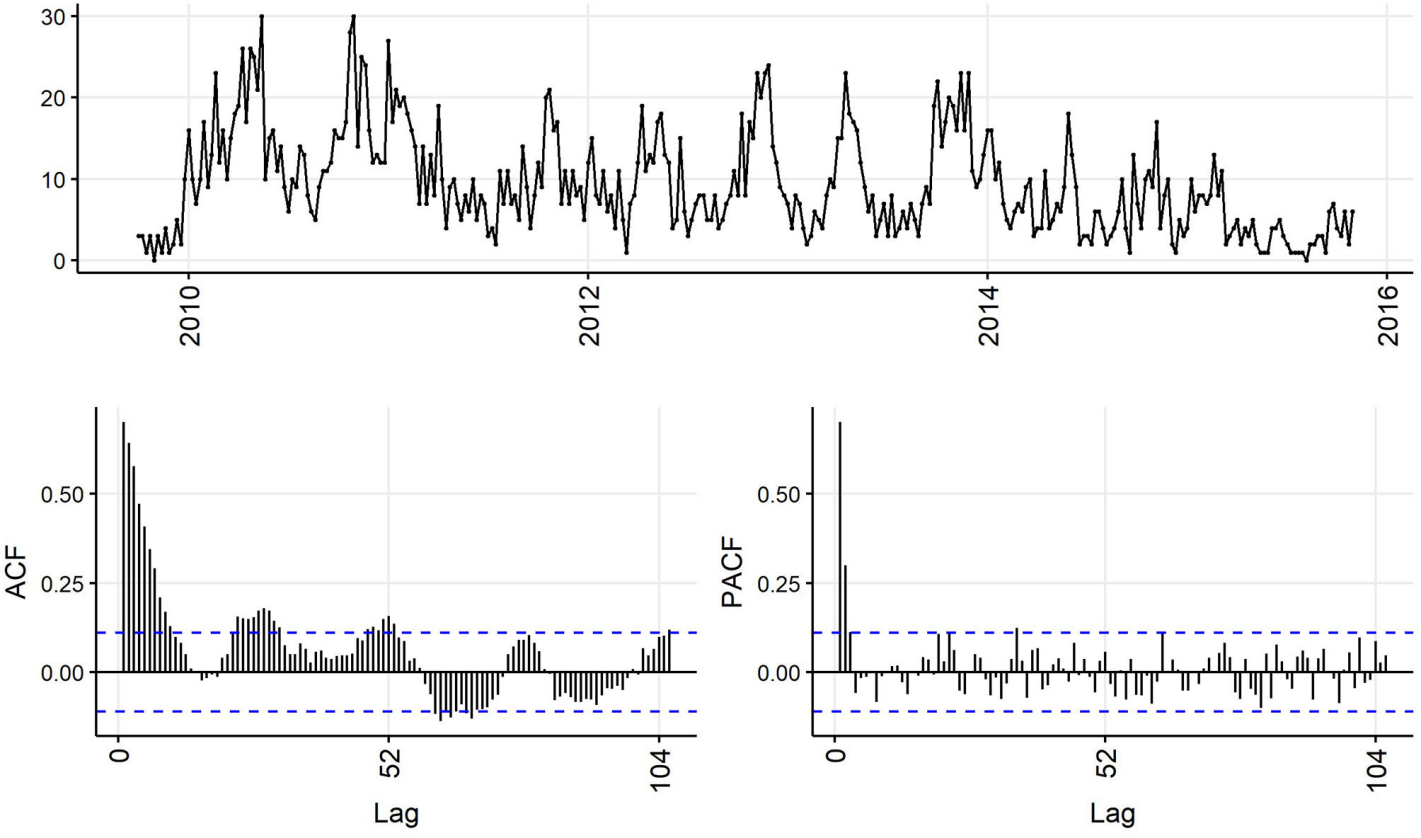
Time-series **(top)**, autocorrelation function **(left)**, and partial autocorrelation function **(right)** plots of weekly aggregated confirmed canine parvovirus events reported in a surveillance system in Australia, 2009–2015.

**Figure 7:**
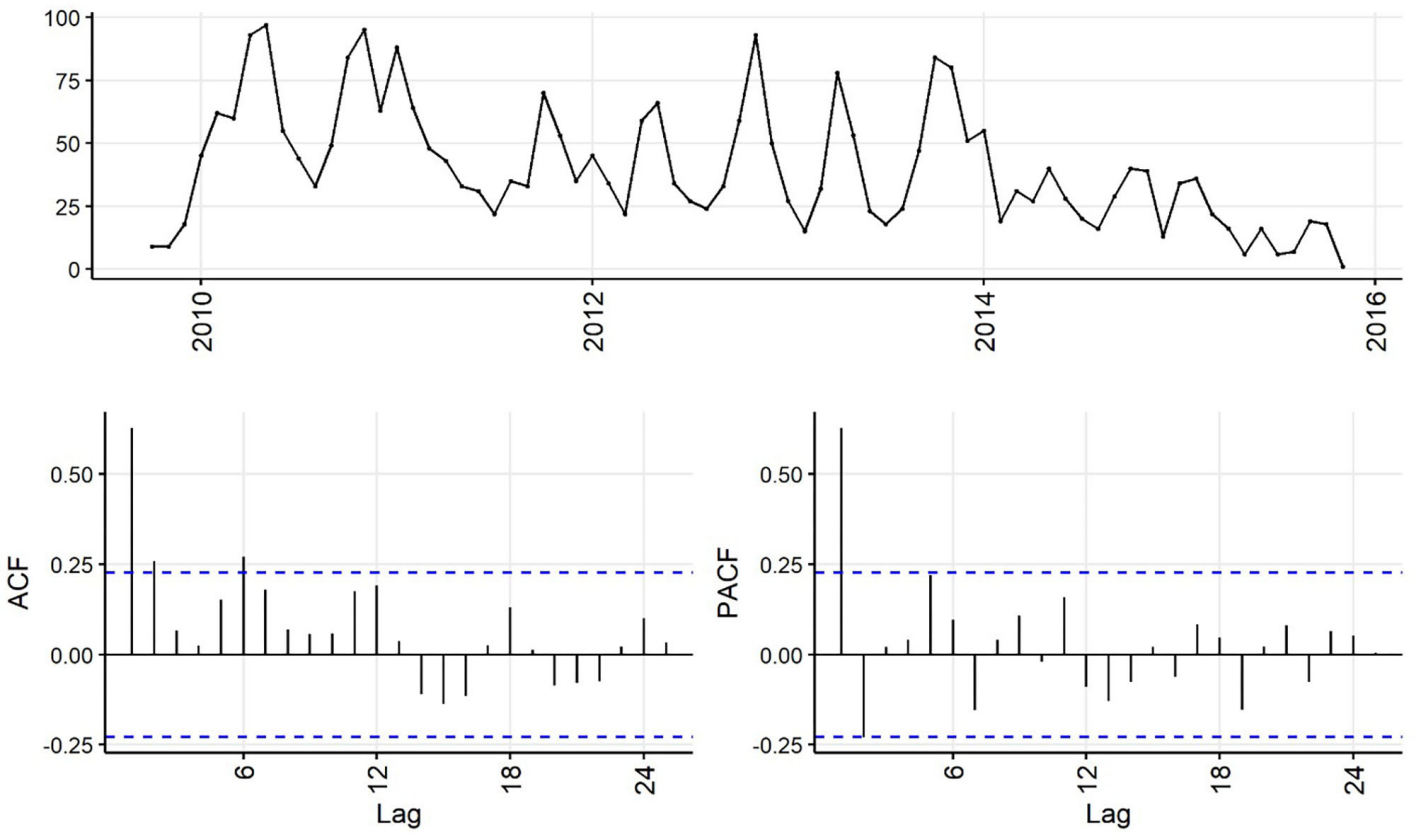
Time-series **(top)**, autocorrelation function **(left)**, and partial autocorrelation function **(right)** plots of monthly aggregated confirmed canine parvovirus events reported in a surveillance system in Australia, 2009–2015.

**Figure 8:**
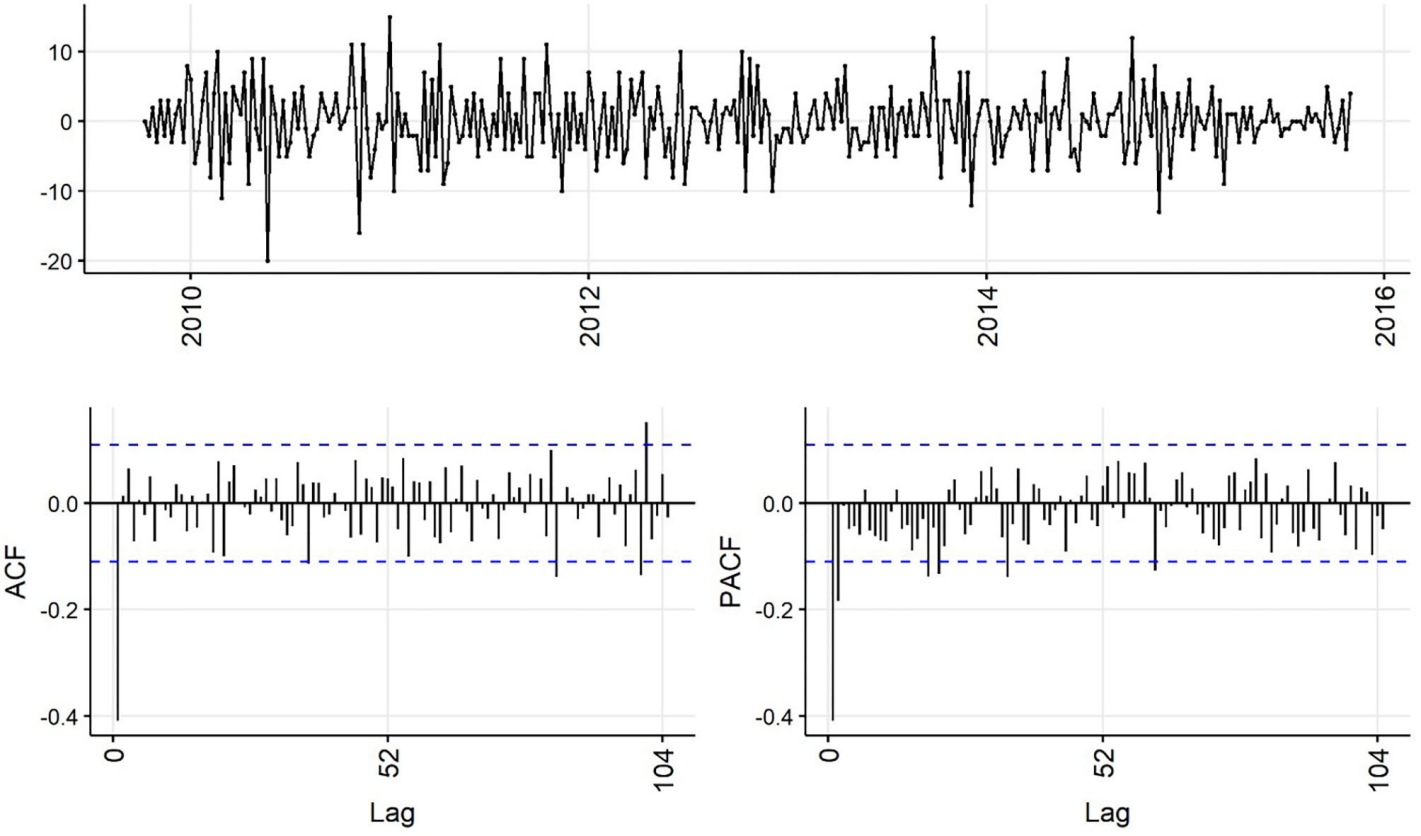
Time-series **(top)**, autocorrelation function **(left)**, and partial autocorrelation function **(right)** plots of differenced weekly aggregated confirmed canine parvovirus events reported in a surveillance system in Australia, 2009–2015.

**Figure 9:**
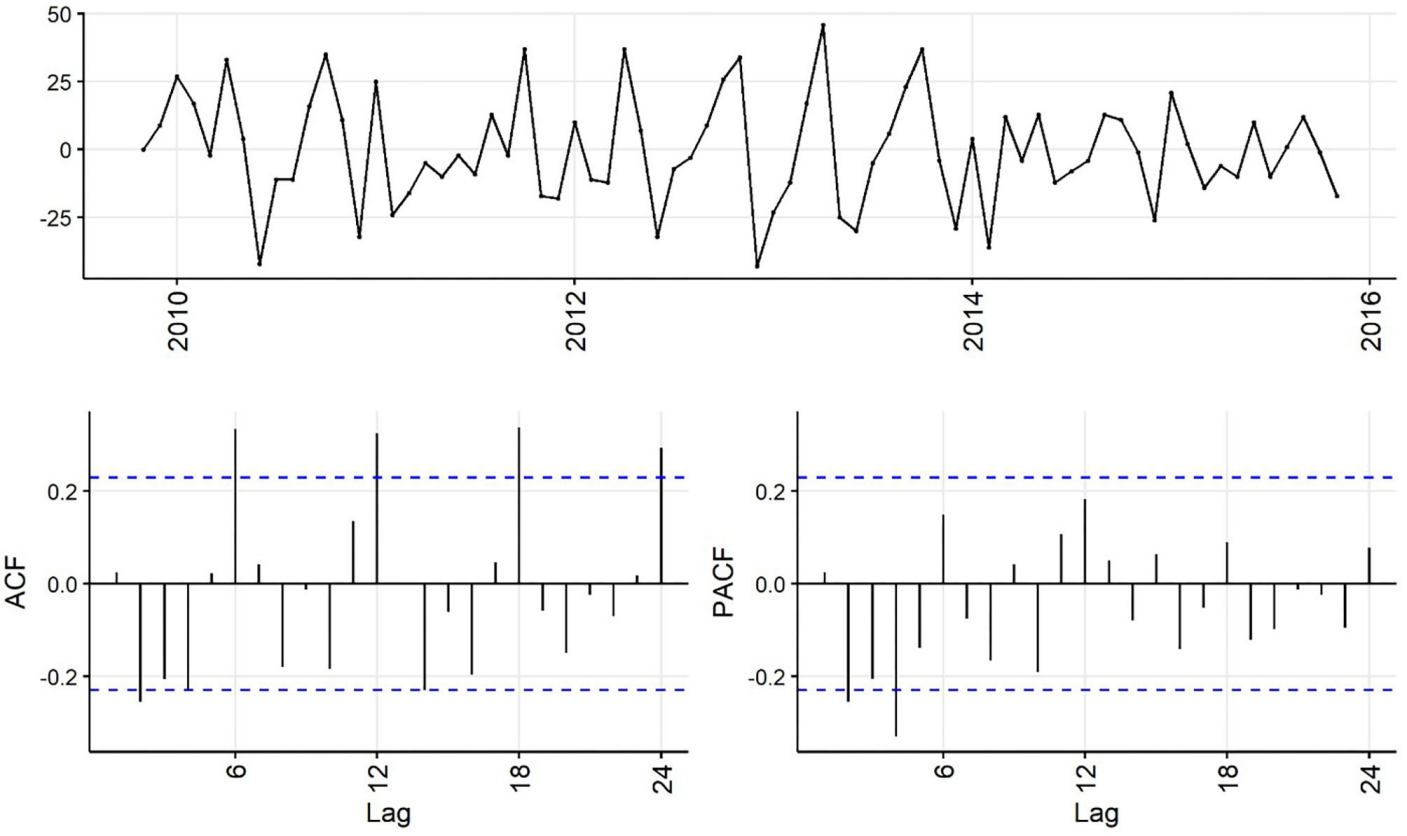
Time-series **(top)**, autocorrelation function **(left)**, and partial autocorrelation function **(right)** plots of differenced monthly aggregated confirmed canine parvovirus events reported in a surveillance system in Australia, 2009–2015.

Decreasing trend in the weekly and monthly time-series, as well as significant autocorrelation of 10 weeks and 2 months in weekly and monthly ACF plots, respectively, suggested non-stationarity (Figures 6,7). Automated testing of both weekly and monthly series with a sequence of KPSS tests suggested that first differencing of one order would make the series stationary for the non-seasonal component of subsequent ARIMA models, and that differencing was not necessary for the seasonal components of these models. The differenced time-series plots and ACF plots of the weekly and monthly time-series were plausibly stationary—trend was less apparent and there was only one lag of significant autocorrelation in the weekly ACF plot and no initially autocorrelated lags in the monthly ACF plot (Figures 8,9).

Statistical tests of the weekly and monthly raw time-series were consistent with these findings (Table 1). Ljung-Box tests of both time-series suggested non-independence (*P* < 0.05). This was expected due to the autocorrelation observed in the ACF plots. ADF tests of both series suggested stationarity around trend (*P* > 0.05). This was consistent with the time-series plots in which there was decreasing trend but symmetrical oscillation of the series around this trend. KPSS tests for trend-stationarity also suggested trend-stationarity for both time-series (*P* > 0.1), and lack of level-stationarity (trend was present) for both series (*P* = 0.01).

**Table 1:** *P*-values of statistical tests for stationarity on weekly and monthly time-series of confirmed canine parvovirus events reported in a surveillance system in Australia, 2009−2015.

	**Weekly**		**Monthly**	
**Test**	**Raw**	**Differenced**	**Raw**	**Differenced**
Ljung-Box	<0.001	<0.001	<0.001	<0.001
ADF	0.86	0.42	0.95	0.60
KPSS trend	>0.1	>0.1	>0.1	>0.1
KPSS level	0.01	>0.1	0.01	>0.1

*ADF, augmented Dickey Fuller; KPSS, Kwiatowski-Phillips-Schmidt-Shin. P > 0.05 indicates failure to reject the null hypothesis*.

Statistical test results for stationarity of the differenced time-series were similar, except that the KPSS test suggested level-stationarity (no trend) for both series. Overall, given the observed series, ACF and PACF plots and the findings of the statistical tests, both differenced time-series appeared more stationary than raw weekly and monthly time-series, suggesting *d* = *1* of the non-seasonal part of the weekly and monthly ARIMA models.

The ACF plot of the weekly time-series has a fast initial decay with only the first lag significant. This indicates *q* = *1* for the weekly ARIMA model. The ACF plot of the monthly time-series has limited autocorrelation at 2 lags. This could indicate *q* = *0*−*2* for the monthly ARIMA model. The PACF plot of the weekly data has a fast decay with significant partial autocorrelation in the first two lags. This suggests *p* = *2*. The PACF plot for the monthly data has limited partial autocorrelation significant. This suggests *p* = *0*−*2* for the monthly ARIMA model.

For seasonality, there are spikes in the weekly ACF at approximately 2 years, indicating *Q* = *1*−*2*. There are 3 spikes around 6 months in the PACF, indicating *P* = *3*. For seasonality in the monthly data, there are consistent spikes at 6 months, suggesting *Q* = *2*, and limited spikes in the PACF, suggesting *P* = *0*−*1*.

Auto-fitted ARIMA models for weekly and monthly time-series had (p, d, q) (P, D, Q) [m] structures (3,1,1) (1,0,0) [52] with drift to allow a decreasing trend over time (AICc = 1834.24) and (2,1,1) (2,0,0) [12] (AICc = 632.61). Other simpler structures were assessed with reduced orders for *(p, d, q) (P, D, Q)* that were still within the orders estimated in the exploratory analysis.

The final selected ARIMA models for the weekly and monthly time-series had the structures (2,1,1) (1,0,0) [52] with drift and (2,1,1) (2,0,0) [12] (auto-fitted model), respectively. Parameter values are shown in Tables 2,3. These models had the simplest structure and lowest AICc, plausible forecast plots, and reasonably normally distributed residuals that were time-independent (ACF plots of residuals and Ljung–Box test; *P* > 0.05).

**Table 2:** Coefficients and 95% confidence intervals for parameters in an ARIMA model fitted to a weekly time-series of confirmed canine parvovirus events reported in a surveillance system in Australia, 2009−2015.

**Parameter**	**Coefficient**	**95% range**
AR1	0.46	0.41 – 0.52
AR2	0.30	0.23 – 0.37
MA1	−1.00	−1.02 – −0.98
SAR1	0.11	−0.04 – 0.19
Drift	−0.02	−0.04 – −0.00

**Table 3:** Coefficients and 95% confidence intervals for parameters in an ARIMA model fitted to a monthly time-series of confirmed canine parvovirus events reported in a surveillance system in Australia, 2009−2015.

**Parameter**	**Coefficient**	**95% range**
AR1	0.80	0.51 – 1.08
AR2	−0.29	−0.56 – −0.03
MA1	−0.90	−1.12 – −0.68
SAR1	0.16	−0.09 – 0.39
SAR2	0.29	0.03 – 0.56

#### Step 6: Multivariate Analysis

**Figure 10:**
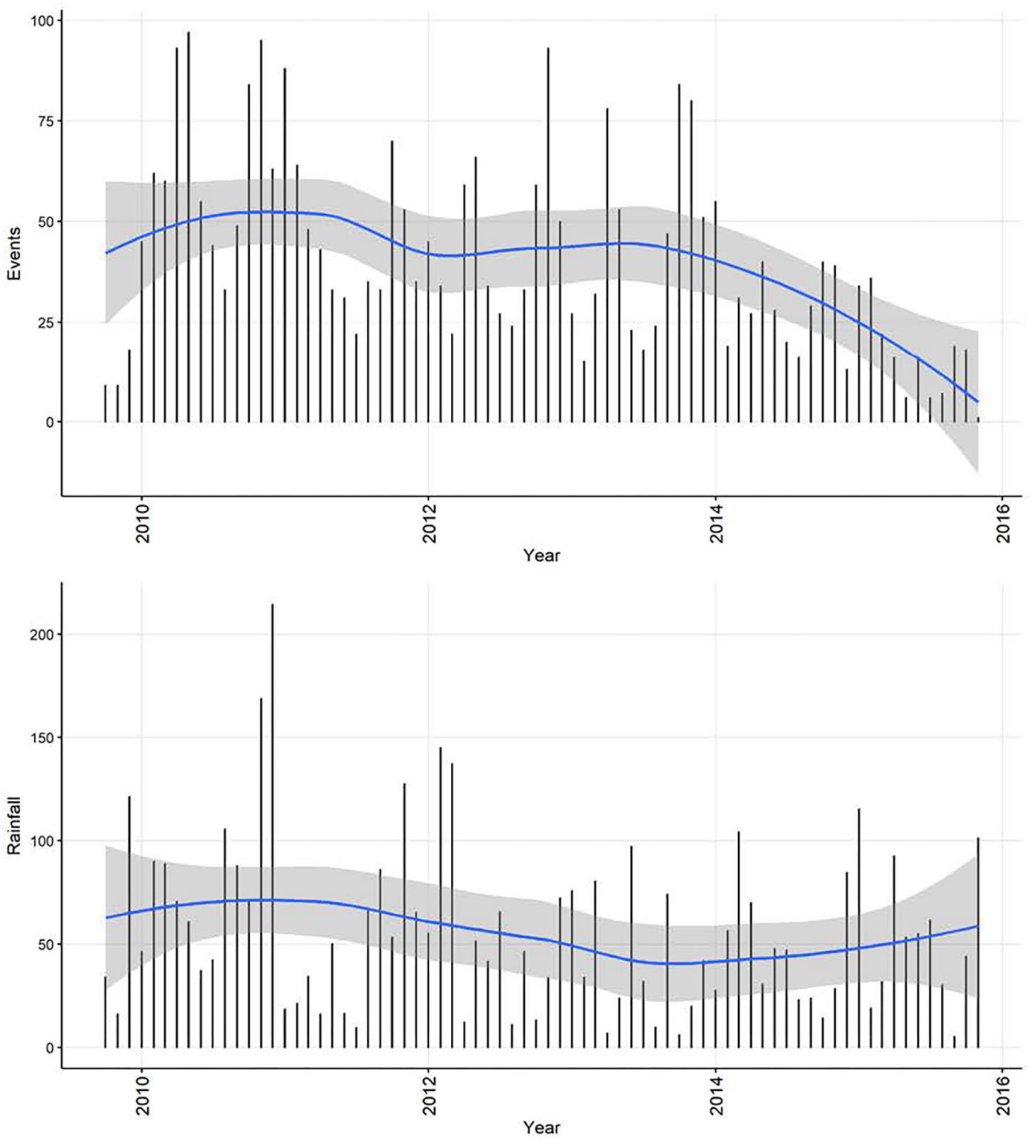
Time-series of confirmed canine parvovirus events reported in a surveillance system in Australia, 2009–2015 **(top)** and corresponding rainfall **(below)** recorded from Mudgee, NSW (32.58°S, 149.58°E). Blue line, loess smoothed curve of events/month with 95% CI (gray).

Although decomposition of the monthly rainfall time-series (Figure 10) suggested a decreasing trend and seasonality, neither were quantitatively significant. The rainfall time-series appeared stationary (visually as a time-series and with ACF and PACF plots, and also following statistical tests). Automated model fitting of an ARIMA model of the monthly CPV events time-series with rainfall as a predictor suggested that there was an association between the previous 3 months' rainfall and CPV events (Table 4). The coefficients indicate that current and prior rainfall are associated with an increase in parvovirus cases—whilst current and recent (1–2 month lags) rainfall are associated with an increase in cases currently, rainfall 3 months previously are associated with a reduction in cases reported. Although all but the 1 month rainfall lag coefficient are not statistically significant (95% CIs include 1; *P* > 0.05), all variables are required in this model to produce the best model fit.

**Table 4:** Coefficients and 95% confidence intervals for parameters in a Vector Auto-regressive model fitted to a monthly time-series of confirmed canine parvovirus events and rainfall reported in a surveillance system in Australia, 2009–2015.

**Parameter**	**Coefficient**	**95% range**
AR1	0.58	0.30 – 0.86
AR2	−0.32	−0.56 – −0.08
MA1	−0.79	−0.98 – −0.60
SAR1	0.17	−0.05 – 0.39
SAR2	0.43	0.19 – 0.67
Rainfall 0 m lag	0.07	−0.01 – 0.16
Rainfall 1 m lag	0.12	0.04 – 0.21
Rainfall 2 m lag	0.06	−0.03 – 0.15
Rainfall 3 m lag	−0.10	−0.18 – −0.01

### Interpretation

Without detailed interpretation of the epidemiology of CPV in NSW, general interpretation of the output from the above analysis and some observations are as follows. During the study period, most cases of CPV occurred as individual case reports rather than events, but focusing on events (in which cases are likely epidemiologically-linked) produces information that is more meaningful for disease control and prevention. The selection criteria applied mean that this event time-series is accurate, even though it might not represent the entire study population (owned dogs in NSW between 2009 and 2015) because of the voluntary nature of reporting within the surveillance system. The length of the time-series analyzed is 2,218 days. Although this is a large size (*N*), daily fluctuations in reporting necessitate aggregation to the week and month level to better understand trends and patterns. In addition, knowledge of daily patterns of occurrence and reporting are unlikely to match the temporal scale of disease causation investigations and management of the disease.

The apparent decrease in reported events during the period—especially toward the end of the series—likely reflects decreasing enthusiasm for reporting in the surveillance system and then an extended period of it being decommissioned. In addition to this long-term trend in the CPV event data, seasonality was apparent. This makes biological sense, since virus survival is affected by climatic factors (25), dog management and behavior can vary with the seasons and human activity, and breeding cycles might add additional seasonality to CPV transmission. Analysis also demonstrates how monthly aggregated data is better than weekly aggregated data (and by implication, daily reported data) for highlighting the seasonal patterns. Removing trend and seasonal components, the remainder of this series had a regularly repeating pattern, indicating that this series of CPV events can be described using an ARMA or ARIMA model. Through a series of documented procedures, ARIMA models fit to the weekly and monthly CPV event series had (generally positive) seasonal and non-seasonal autoregression parameters of order 1 or 2 and a negative non-seasonal moving average of order 1. This indicates that the occurrence of CPV depends on preceding CPV in the relatively short term (prior 1 or 2 weeks or months, or season), modulated negatively by short term variation. This can be interpreted as a disease that responds quickly to recent conditions, consistent with the dynamic transmission of CPV within domestic dog populations. Inclusion of rainfall as a predictor of monthly CPV events did not change the model structure or modify parameter estimates substantially, but indicated that increased rainfall in the previous 2 months and lower rainfall in the month before this is associated with increased number of CPV events reported in the current month. In addition, model fit (AICc) to the data is improved by the inclusion of rainfall, suggesting that rainfall might play a role in the pattern of CPV occurrence. Again, this association might be explained via virus survival or dog behavior (25, 26).

## Conclusions and Recommendations

To increase the application of methods to analyse time-series data in veterinary epidemiology we recommend that wherever feasible, such time-series data be made available both for analysis and for methods development. We recommend that time-series data be made available, because of those studies identified in our literature scan and reviewed, about one-third described time-series data but failed to use time-series analysis methods; rather, the data were summarized without exploring temporal trends and patterns and autocorrelation. Application of time-series analysis methods has the potential to generate further insight into the occurrence and distribution of animal diseases, disease causation and how it can be used to facilitate surveillance and disease control.

In addition, we recommend that further efforts are made to make analysis of time-series data (whether in R or other software platforms) more user-friendly and accessible. Although lack of availability of data of sufficient length can preclude time-series analysis, lack of familiarity with analytical methods and available software might also limit the information generated by such analyses. In addition, we also recommend that epidemiologic assumptions underlying the analysis of time-series data—particularly a constant population at-risk, non-sparse data, and sources of bias—be thoroughly investigated in veterinary studies. We have not described such investigations here, because they are common to all epidemiologic analyses using observational data.

With developments in monitoring and surveillance systems, and some systems being in existence for extended periods of time, we expect more time-series data to become available together with more software options. However, time-series applications require further promotion to increase adoption and use in veterinary epidemiology. Given that the most often cited advantage of using time-series analyses is the ability to predict disease occurrence, contributing to early warning and therefore disease prevention, application of this analytical method in veterinary epidemiology and preventive medicine is warranted.

## Data Availability Statement

All datasets generated for this study are included in the article, see (8).

## Author Contributions

MW conceived the idea and analyzed data from the literature scan. VB developed the R code. MW, RI, and VB reviewed the literature and drafted the manuscript. All authors contributed to the article and approved the submitted version.

## Conflict of Interest

The authors declare that the research was conducted in the absence of any commercial or financial relationships that could be construed as a potential conflict of interest.

## Footnotes

^1^http://www.bom.gov.au/climate/data/ accessed 30 September 2019.
