# Supplementary material for: Autoregressive Models Applied to Time-Series Data in Veterinary Science
Source: Front Vet Sci. 2020 Sep 17;7:604. doi: 10.3389/fvets.2020.00604 (PMC7527444; doi:10.3389/fvets.2020.00604)
Supplement: Supplementary file 1 [file Table_1.DOCX]

Supplementary Material

# Supplementary Table 1

Template use to extract data from a scan of the literature using the search terms TOPIC: (time-series) and TOPIC: (analysis) and TOPIC: (veterinary) during the timespan 1980 to present (31 August 2019), in CAB Abstracts Index via Web of Science

| **Is this paper within scope?**  Yes No Don’t know  For the time-series described – prior to any data editing or modification – extract the following (if more than one):   \| **A. Time series** \| \| --- \| \| 1. Location (country(ies), state/province/other second-level administrative unit): \| \|  \| \| 1. Data source of primary event (e.g. surveillance system, primary research): \| \| 1. Study population (species, inclusion/exclusion criteria): \| \| 1. Time period: \| \|  \| \| 1. Time unit of raw data: \| \|  \| \| 1. Size (number of units): \| \|  \| \| 1. Primary event (“outcome”) of interest: \| \|  \| \| 1. Measurement of primary event: \| \|  \| \| 1. Secondary events/outcomes: \| \|  \| \| 1. Covariate information, list individually and time units: \| \|  \| \| 1. Purpose of study: Analysis/Description/Prediction \| \| **B. Data analysis** \| \| 1. Describe any data editing or manipulation of the primary outcome event \| \|  \| \| 1. Was trend analysis performed, and how? \| \|  \| \| 1. Was autoregression analysis performed, and how? \| \|  \| \| 1. Was seasonality analysis performed, and how? \| \|  \| \| 1. Was differencing used, and why? \| \|  \| \| 1. Methods used to check stationarity? \| \| 1. Were ARMA or ARIMA models fit, and how? \| \|  \| \| 1. Were other models fit? Describe. \| \|  \| \| 1. Was forecasting undertaken, and how? \| \|  \| \| 1. What software was used? \| \|  \| \| 1. How were outputs displayed? Time-series plots, ACF plots, PACF plots, other \| \|  \| \| **C. Discussion** \| \| 1. Stated advantages of time-series analysis \| \|  \| \| 1. Stated barriers to time-series analysis \| \|  \| \| **D. Definitions** \| \| 1. List any time-series terms that are defined \| \|  \| \|  \| \|  \| \|  \| |
| --- | --- | --- | --- | --- | --- | --- | --- | --- | --- | --- | --- | --- | --- | --- | --- | --- | --- | --- | --- | --- | --- | --- | --- | --- | --- | --- | --- | --- | --- | --- | --- | --- | --- | --- | --- | --- | --- | --- | --- | --- | --- | --- | --- | --- | --- | --- | --- | --- | --- | --- | --- | --- | --- |

# Supplementary Table 2

Published articles identified on a scan of the literature using the topic terms (time-series) and (analysis) and (veterinary), 1980 to 31 August 2019, restricted to journal articles only. Of the 60 articles identified, 5 were unavailable and 18 were deemed out-of-scope for the current study.

| **ID** | **Year** | **Scope** | **Country** | **Data source** | **Study population** | **Time period** | **Time unit** | **Size** | **Primary event** | **Measure-ment** | **Study purpose** | **Trend analysis** | **Auto-regression** | **Season-ality** | **Station-arity** | **ARIMA** |
| --- | --- | --- | --- | --- | --- | --- | --- | --- | --- | --- | --- | --- | --- | --- | --- | --- |
| **1** | 2019 | No | − | − | − | − | − | − | − | − | − | − | − | − | − | − |
| **2** | 2019 | Yes | Russia | Monitoring | *Ixodes persulcatus* | 1982–2017 | Season | 23 | Number of ticks | Observation | Analysis | Yes | Yes | No | Yes | No |
| **3** | 2019 | No | − | − | − | − | − | − | − | − | − | − | − | − | − | − |
| **4** | 2018 | No | − | − | − | − | − | − | − | − | − | − | − | − | − | − |
| **5** | 2018 | Yes | Global | Surveillance | Poultry | 2006–2016 | Month | 132 | HPAI outbreaks | Observation | Descriptive | Yes | No | Yes | No | No |
| **6** | 2018 | Yes | Costa Rica | Surveillance | Cattle | 1985–2016 | Month | 372 | Rabies outbreaks | Laboratory | Analysis | No | Yes | Yes | No | No |
| **7** | 2018 | No | − | − | − | − | − | − | − | − | − | − | − | − | − | − |
| **8** | 2018 | − | − | − | − | − | − | − | − | − | − | − | − | − | − | − |
| **9** | 2018 | No | − | − | − | − | − | − | − | − | − | − | − | − | − | − |
| **10** | 2018 | Yes | France | Surveillance | Cattle, swine, poultry | 2002–2015 | Month | 156 | Resistant *E.coli* strains | Laboratory | Prediction | Yes | No | Yes | No | Yes |
| **11** | 2018 | Yes | The Netherlands | Monitoring | Pigs | 2011–2014 | Day | 1290 | Cases of pleurisy, pneumonia | Laboratory | Analysis | Yes | Yes | Yes | No | No |
| **12** | 2017 | No | − | − | − | − | − | − | − | − | − | − | − | − | − | − |
| **13** | 2017 | Yes | Japan | Surveillance | Pigs | 2013–2015 | Day | 4380 | Number of hydatid infected livers | Observation | Prediction | No | Yes | No | No | Yes |
| **14** | 2017 | Yes | Spain | Monitoring | Dairy cattle | 2006–2013 | Month | 416 | Number of carcasses collected | Observation | Analysis | Yes | Yes | Yes | Yes | Yes |
| **15** | 2017 | Yes | Brazil | Monitoring | Dairy farms | 2011–2016 | Month | 72 | Bulk tank somatic cell count | Laboratory | Analysis | No | Yes | Yes | No | Yes |
| **16** | 2017 | Yes | Spain | Surveillance | Beef, dairy cattle | 2007–2011 | Day | 1460 | Cattle carcases collection | Observation | Descriptive | Yes | Yes | Yes | No | No |
| **17** | 2017 | Yes | China | Surveillance | Poultry | 2006–2015 | Month | 120 | Avian cholera cases | Laboratory | Analysis | Yes | Yes | Yes | Yes | No |
| **18** | 2017 | Yes | USA, Canada | Clinical records | Equine | 2006–2012 | Day | 2555 | Antimicrobial prescriptions | Observation | Descriptive | Yes | No | Yes | No | No |
| **19** | 2017 | No | − | − | − | − | − | − | − | − | − | − | − | − | − | − |
| **20** | 2016 | − | − | − | − | − | − | − | − | − | − | − | − | − | − | − |
| **21** | 2016 | Yes | Zambia | Surveillance | Poultry | 1999–2014 | Month | 192 | Cases of Newcastle Disease | Observation | Prediction | No | No | No | No | Yes |
| **22** | 2016 | Yes | Australia | Surveillance | Dogs, cats | 2011–2013 | Month | 24 | Tick paralysis reports | Observation | Analysis | No | No | No | No | No |
| **23** | 2016 | Yes | United Kingdom | Surveillance | Cattle herds | 2008–2014 | Month | 84 | TB herd tests | Laboratory | Analysis | Yes | Yes | Yes | Yes | No |
| **24** | 2015 | Yes | Japan | Monitoring | Pigs | 2003–2013 | Day | 1922 | Mycobacteria condemns | Observation | Analysis | No | Yes | Yes | Yes | Yes |
| **25** | 2015 | Yes | Switzerland | Monitoring | Cattle | 2009–2011 | Day | 730 | On-farm deaths, stillbirths | Observation | Descriptive | Yes | Yes | Yes | Yes | Yes |
| **26** | 2015 | Yes | Canada | Clinical records | Companion animals | 2007–2010 | Day | 1460 | Cases of enteric syndrome | Observation | Descriptive | Yes | No | Yes | No | No |
| **27** | 2015 | − | − | − | − | − | − | − | − | − | − | − | − | − | − | − |
| **28** | 2015 | No | − | − | − | − | − | − | − | − | − | − | − | − | − | − |
| **29** | 2015 | Yes | Spain | Monitoring | Cattle | 2005–2010 | Day | 2190 | Dead cattle collections | Observation | Prediction | Yes | No | Yes | No | No |
| **30** | 2015 | No | − | − | − | − | − | − | − | − | − | − | − | − | − | − |
| **31** | 2014 | No | − | − | − | − | − | − | − | − | − | − | − | − | − | − |
| **32** | 2014 | No | − | − | − | − | − | − | − | − | − | − | − | − | − | − |
| **33** | 2014 | Yes | USA | Laboratory records | Dogs | 2000–2010 | Day | 4015 | Leptospirosis seropositivity | Laboratory | Descriptive | Yes | No | Yes | No | No |
| **34** | 2014 | Yes | Indonesia | Laboratory records | Humans and livestock | 1897–1917 | Month | 240 | Human, livestock rabies cases | Observation | Analysis | Yes | Yes | No | No | Yes |
| **35** | 2013 | No | − | − | − | − | − | − | − | − | − | − | − | − | − | − |
| **36** | 2013 | Yes | United Kingdom | Surveillance | Dairy cattle | 1997–2007 | Day | 4015 | Left displaced abomasum, uterine torsion cases | Observation | Analysis | Yes | Yes | Yes | No | Yes |
| **37** | 2013 | No | − | − | − | − | − | − | − | − | − | − | − | − | − | − |
| **38** | 2013 | Yes | Canada | Laboratory records | Cattle herds | 2008–2010 | Day | 782 | Herd syndromes | Observation | Descriptive | Yes | No | Yes | No | No |
| **39** | 2012 | Yes | United Kingdom | Monitoring | Pigs | 2005–2011 | Day | 2555 | Enzootic pneumonia | Observation | Descriptive | Yes | No | Yes | No | No |
| **40** | 2011 | Yes | New Zealand | Clinical records | Dairy cattle | 1995–2007 | Day | 1945 | Cases of lameness | Observation | Descriptive | Yes | Yes | Yes | No | No |
| **41** | 2010 | − | − | − | − | − | − | − | − | − | − | − | − | − | − | − |
| **42** | 2010 | Yes | Australia | Research | Beef cattle | 2002–2007 | Month | 72 | CCUO cases | Observation | Analysis | Yes | Yes | Yes | No | No |
| **43** | 2010 | Yes | Brazil | Monitoring | Poultry | 1996–2005 | Day | 3495 | Carcass condemns | Observation | Descriptive | Yes | No | Yes | No | No |
| **44** | 2008 | No | − | − | − | − | − | − | − | − | − | − | − | − | − | − |
| **45** | 2008 | Yes | USA | Surveillance | Humans, wild birds, mosquitoes | 2005–2006 | Week | 44 | WNV seroprevalence | Laboratory | Analysis | No | No | No | No | No |
| **46** | 2008 | Yes | USA | Surveillance | Mosquitoes | 2002–2005 | Week | 182 | Mosquitoes in light traps | Observation | Analysis | Yes | Yes | Yes | Yes | Yes |
| **47** | 2007 | Yes | Kazakhstan | Research | Great gerbils | 1949–1996 | Biannual | 94 | Plague test positive | Laboratory | Analysis | Yes | Yes | Yes | No | No |
| **48** | 2007 | No | − | − | − | − | − | − | − | − | − | − | − | − | − | − |
| **49** | 2006 | No | − | − | − | − | − | − | − | − | − | − | − | − | − | − |
| **50** | 2004 | No | − | − | − | − | − | − | − | − | − | − | − | − | − | − |
| **51** | 2004 | Yes | Denmark | Clinical records | Pig herds | 2001 | Month | 12 | Systemic, respiratory, diarrhoea syndromes | Observation | Prediction | No | Yes | No | No | No. |
| **52** | 2004 | Yes | Israel | Surveillance | Sheep, cattle | 1968–2002 | Month | 408 | Bluetongueoutbreaks | Observation | Analysis | Yes | Yes | Yes | No | No |
| **53** | 2004 | Yes | Israel | Surveillance | Cattle, goats, sheep | 1968–2002 | Month | 240 | Bluetongue outbreaks | Observation | Analysis | Yes | No | Yes | No | No |
| **54** | 2002 | Yes | UK | Research | *Lucilia sericata* | 1990-2000 | Day | 2354 | Blowfly density | Observation | Analysis | Yes | No | No | No | No |
| **55** | 2002 | Yes | USA, Canada | Clinical records | Dogs | 1983–1998 | Day | 5475 | Leptospirosis cases | Observation | Descriptive | Yes | Yes | Yes | Yes | Yes |
| **56** | 1995 | Yes | USA | Clinical records | Cats, dogs | 1984–1991 | Month | 96 | Endoparasitic infections | Laboratory | Descriptive | Yes | Yes | Yes | No | No |
| **57** | 1992 | − | − | − | − | − | − | − | − | − | − | − | − | − | − | − |
| **58** | 1991 | Yes | Northern Ireland | Monitoring | Sheep | 1970–1986 | Month | 192 | Abattoir cases of fascioliasis | Observation | Predictive | No | Yes | Yes | Yes | Yes |
| **59** | 1990 | No | − | − | − | − | − | − | − | − | − | − | − | − | − | − |
| **60** | 1990 | Yes | Northern Ireland | Monitoring | Sheep | 1969–1987 | Day | 6840 | Liver condemns due to fascioliasis | Observation | Predictive | No | Yes | No | No | Yes |

1. Kapitany-Foveny, M., Ferenci, T., Sulyok, Z., Kegele, J., Richter, H., Valyi-Nagy, I., Sulyok, M. Can Google Trends data improve forecasting of Lyme disease incidence? Zoonoses and Public Health 66(1), 101-107; 2019, 10.1111/zph.12539.
2. Bugmyrin, S. V., Bespyatova, L. A., Korotkov, Yu. S. Long-term dynamics of Ixodes persulcatus (Acari: Ixodidae) abundance in the north-west of its range (Karelia, Russia). Experimental and Applied Acarology 77(2), 229-240, 2019, 10.1007/s10493-019-00342-y.
3. Santos, C. A. G., Guerra-Gomes, I. C., Gois, B. M., Peixoto, R. F., Keesen, T. S. L., da Silva, R. M. Correlation of dengue incidence and rainfall occurrence using wavelet transform for Joao Pessoa city. Science of the Total Environment 647, 794-805; 2019, 10.1016/j.scitotenv.2018.08.019
4. Jaenson, T. G. T., Petersson, E. H., Jaenson, D. G. E., Kindberg, J., Pettersson, J. H. O., Hjertqvist, M., Medlock, J. M., Bengtsson, H. The importance of wildlife in the ecology and epidemiology of the TBE virus in Sweden: incidence of human TBE correlates with abundance of deer and hares. Parasites and Vectors 11(477); 2018, 10.1186/s13071-018-3057-4
5. Awada, L., Tizzani, P., Noh, S. M., Ducrot, C., Ntsama, F., Caceres, P., Mapitse, N., Chalvet-Monfray, K. Global dynamics of highly pathogenic avian influenza outbreaks in poultry between 2005 and 2016 - focus on distance and rate of spread. Transboundary and Emerging Diseases 65(6), 2006-2016; 2018.
6. Hutter, S. E., Kasbohrer, A., Gonzalez, S. L. F., Leon, B., Brugger, K., Baldi, M., Romero, L. M., Gao Yan, Chaves, L. F. Assessing changing weather and the El Nino Southern Oscillation impacts on cattle rabies outbreaks and mortality in Costa Rica (1985-2016). BMC Veterinary Research 14(285); 2018; 10.1186/s12917-018-1588-8
7. Lord, J. S., Hargrove, J. W., Torr, S. J., Vale, G. A. Climate change and African trypanosomiasis vector populations in Zimbabwe's Zambezi Valley: a mathematical modelling study. PLoS Medicine 15(10), e1002675; 2018; 10.1371/journal.pmed.1002675
8. Gao Xiang, Xiao JianHua, Wang HongBin. Prediction of avian pasteurellosis based on ZINB model and meteorological factors. Transactions of the Chinese Society of Agricultural Engineering, 34(15), 176-182; 2018.
9. Brugger, K., Walter, M., Chitimia-Dobler, L., Dobler, G., Rubel, F. Forecasting next season's Ixodes ricinus nymphal density: the example of southern Germany 2018. Experimental and Applied Acarology 75(3), 281-288; 2018; 10.1007/s10493-018-0267-6
10. Boireau, C., Morignat, E., Cazeau, G., Jarrige, N., Jouy, E., Haenni, M., Madec, J. Y., Leblond, A., Gay, E. Antimicrobial resistance trends in Escherichia coli isolated from diseased food-producing animals in France: a 14-year period time-series study. Zoonoses and Public Health 65(1), e86-e94; 2018; 10.1111/zph.12412
11. Hulsegge, B., de Greef, K. H. A time-series approach for clustering farms based on slaughterhouse health aberration data. Preventive Veterinary Medicine, 153, 64-70; 2018; 10.1016/j.prevetmed.2018.03.003
12. Brugger, K., Walter, M., Chitimia-Dobler, L., Dobler, G., Rubel, F. Seasonal cycles of the TBE and lyme borreliosis vector Ixodes Ricinus modelled with time-lagged and interval-averaged predictors. Experimental and Applied Acarology 73(3/4), 439-450; 2017; 10.1007/s10493-017-0197-8
13. Adachi, Y., Makita, K. Time series analysis based on two-part models for excessive zero count data to detect farm-level outbreaks of swine echinococcosis during meat inspections. Preventive Veterinary Medicine 148, 49-57, 2017; 10.1016/j.prevetmed.2017.10.001
14. Fernandez-Fontelo, A., Puig, P., Caceres, G., Romero, L., Revie, C., Sanchez, J., Dorea, F. C., Alba, A. Enhancing syndromic surveillance for fallen dairy cattle: modelling and detecting mortality peaks at different administrative levels. Epidemiologie et Sante Animale 72, 15-26; 2017
15. Busanello, M., de Freitas, L. N., Winckler, J. P. P., Farias, H. P., Dias, C. T. dos S., Cassoli, L. D., Machado, P. F. Month-wise variation and prediction of bulk tank somatic cell count in Brazilian dairy herds and its impact on payment based on milk quality. Irish Veterinary Journal 70, 26; 2017; 10.1186/s13620-017-0103-z
16. Fernandez-Fontelo, A., Fontdecaba, S., Alba, A., Puig, P. Integer-valued AR processes with Hermite innovations and time-varying parameters: an application to bovine fallen stock surveillance at a local scale. Statistical Modelling 17(3), 172-195; 2017; 10.1177/1471082X16683113
17. Qin, H. Y., Xiao, J. H., Li, J. X., Gao, X., Wang, H. B. Climate variability and avian cholera transmission in Guangxi, China. Brazilian Journal of Poultry Science 19(2), 211-219; 2017
18. Welsh, C. E., Parkin, T. D. H., Marshall, J. F. Use of large-scale veterinary data for the investigation of antimicrobial prescribing practices in equine medicine. Equine Veterinary Journal, 49(4), 425-432; 2017; 10.1111/evj.12638
19. Liu, K. K., Wang, T., Huang, X. D., Wang, G. L., Xia, Y., Zhang, Y. T., Jing, Q. L., Huang, J. W., Liu, X. X., Lu, J. H., Hu, W. B. Risk assessment of dengue fever in Zhongshan, China: a time-series regression tree analysis. Epidemiology and Infection 145(3), 451-461; 2017
20. Da Silva, S. R., Lourenco, E. C., Tassinari, W. de S., Famadas, K. M. Descriptive study of the historical series of tick occurrence in dogs assisted in two animal health units in the western zone of the city of Rio de Janeiro. [Estudo descritivo da serie historica de ocorrencia de carrapatos em caes assistidos em duas unidades privadas de saude animal na zona oeste da cidade do Rio de Janeiro]. Revista Brasileira de Medicina Veterinaria. 38(Suppl. 3), 249-259; 2016
21. Mubamba, C., Ramsay, G., Abolnik, C., Dautu, G., Gummow, B. A retrospective study and predictive modelling of Newcastle Disease trends among rural poultry of eastern Zambia. Preventive Veterinary Medicine 133, 97-107; 2016; 10.1016/j.prevetmed.2016.09.017
22. Guernier, V., Milinovich, G. J., Santos, M. A. B., Haworth, M., Coleman, G., Magalhaes, R. J. S. Use of big data in the surveillance of veterinary diseases: early detection of tick paralysis in companion animals. Parasites and Vectors 9, 303; 2016
23. Moustakas, A., Evans, M. R. Regional and temporal characteristics of bovine tuberculosis of cattle in Great Britain. Stochastic Environmental Research and Risk Assessment 30(3), 989-1003; 2016; 10.1007/s00477-015-1140-3
24. Adachi, Y., Makita, K. Real time detection of farm-level swine mycobacteriosis outbreak using time series modeling of the number of condemned intestines in abattoirs. Journal of Veterinary Medical Science 77(9), 1129-1136; 2015; 10.1292/jvms.14-0675
25. Struchen, R., Reist, M., Zinsstag, J., Vial, F. Investigating the potential of reported cattle mortality data in Switzerland for syndromic surveillance. Preventive Veterinary Medicine 121(1/2), 1-7; 2015
26. Anholt, R. M., Berezowski, J., Robertson, C., Stephen, C. Spatial-temporal clustering of companion animal enteric syndrome: detection and investigation through the use of electronic medical records from participating private practices. Epidemiology and Infection 143(12), 2547-2558; 2015
27. Alba-Casals, A., Fernandez-Fontelo, A., Revie, C. W., Dorea, F. C., Sanchez, J., Romero, L., Caceres, G., Perez, A., Puig, P., BE Dufour, B. Development of new strategies to model bovine fallen stock data from large and small subpopulations for syndromic surveillance use. Epidemiologie et Sante Animale 67, 67-76; 2015
28. Vial, F., Berezowski, J. A practical approach to designing syndromic surveillance systems for livestock and poultry. Preventive Veterinary Medicine 120(1), 27-38; 2015; 10.1016/j.prevetmed.2014.11.015
29. Torres, G., Ciaravino, V., Ascaso, S., Flores, V., Romero, L., Simon, F. Syndromic surveillance system based on near real-time cattle mortality monitoring. Preventive Veterinary Medicine 119(3/4), 216-221; 2015
30. Ansari, H., Mansournia, M. A., Izadi, S., Zeinali, M., Mahmoodi, M., Holakouie-Naieni, K. Predicting CCHF incidence and its related factors using time-series analysis in the southeast of Iran: comparison of SARIMA and Markov switching models. Epidemiology and Infection 143(4), 839-850; 2015
31. Geoghegan, J. L., Walker, P. J., Duchemin, J. B., Jeanne, I., Holmes, E. C. Seasonal drivers of the epidemiology of arthropod-borne viruses in Australia. PLoS Neglected Tropical Diseases 8(11), e3325; 2014; 10.1371/journal.pntd.0003325
32. Palo, R. T. Tick-borne encephalitis transmission risk: its dependence on host population dynamics and climate effects. Vector Borne and Zoonotic Diseases 14(5), 346-352; 2014; 10.1089/vbz.2013.1386
33. Lee, H. S., Levine, M., Guptill-Yoran, C., Johnson, A. J., Von Kamecke, P., Moore, G. E. Regional and temporal variations of Leptospira seropositivity in dogs in the United States, 2000-2010. Journal of Veterinary Internal Medicine 28(3), 779-788; 2014; 10.1111/jvim.12335
34. Ward, M. P. Rabies in the Dutch East Indies a century ago - a spatio-temporal case study in disease emergence. Preventive Veterinary Medicine 114(1), 11-20; 2014; 10.1016/j.prevetmed.2014.01.009
35. Dorea, F. C., McEwen, B. J., McNab, W. B., Sanchez, J., Revie, C. W. Syndromic surveillance using veterinary laboratory data: algorithm combination and customization of alerts. PLoS ONE 8(12), e82183; 2013; 10.1371/journal.pone.0082183
36. Lawrence, K., Tulley, W., Laven, R. Observations on the incidence and seasonality of uterine torsion and left displaced abomasum following the 2001 outbreak of foot-and-mouth disease in the UK. Veterinary Journal 196(3), 332-338; 2013; 10.1016/j.tvjl.2012.10.034
37. Lee, H. S., Her Moon, Levine, M., Moore, G. E. Time series analysis of human and bovine brucellosis in South Korea from 2005 to 2010. Preventive Veterinary Medicine 110(2), 190-197; 2013; 10.1016/j.prevetmed.2012.12.003
38. Dorea, F. C., Revie, C. W., McEwen, B. J., McNab, W. B., Kelton, D., Sanchez, J. Retrospective time series analysis of veterinary laboratory data: preparing a historical baseline for cluster detection in syndromic surveillance. Preventive Veterinary Medicine 109(3/4), 219-227; 2013
39. Sanchez-Vazquez, M. J., Nielen, M., Gunn, G. J., Lewis, F. I. Using seasonal-trend decomposition based on loess (STL) to explore temporal patterns of pneumonic lesions in finishing pigs slaughtered in England, 2005-2011. Preventive Veterinary Medicine 104(1/2), 65-73; 2012; 10.1016/j.prevetmed.2011.11.003
40. Lawrence, K. E., Chesterton, R. N., Laven, R. A. Further investigation of lameness in cows at pasture: an analysis of the lesions found in, and some possible risk factors associated with, lame New Zealand dairy cattle requiring veterinary treatment. Journal of Dairy Science 94(6), 2794-2805; 2011; 10.3168/jds.2010-3643
41. Mesquita, M., Pellegrini, D. C. P., Pires Neto, J. A. S., Reis, G. R., Medeiros, C., Corbellini, L. G. Time-series analysis for evaluating serological pattern of bovine leptospirosis in the State of Rio Grande do Sul from 1996 to 2006. [Analise de serie temporal para avaliacao do perfil sorologico da leptospirose bovina no Estado do Rio Grande do Sul de 1996 a 2006]. Arquivos do Instituto Biologico (Sao Paulo) 77(3), 381-387; 2010
42. White, P. J., Ward, M. P., Toribio, J. A. L. M. L., Windsor, P. A. The association between congenital chondrodystrophy of unknown origin (CCUO) in beef cattle and drought in south-eastern Australia. Preventive Veterinary Medicine 94(3/4), 178-184; 2010; 10.1016/j.prevetmed.2010.02.002
43. Moretti, L. d'A., Dias, R. A., Telles, E. O., Balian, S. de C. Time series evaluation of traumatic lesions and airsacculitis at one poultry abattoir in the state of Sao Paulo, Brazil (1996-2005). Preventive Veterinary Medicine 94(3/4), 231-239; 2010; 10.1016/j.prevetmed.2010.02.013
44. Norberg, E., Korsgaard, I. R., Sloth, K. H. M. N., Lovendahl, P. Time-series models on somatic cell score improve detection of mastitis. Acta Agricultura Scandinavica. Section A, Animal Science, 58(4), 165-169; 2008; 10.1080/09064700802621143
45. Hamer, G. L., Walker, E. D., Brawn, J. D., Loss, S. R., Ruiz, M. O., Goldberg, T. L., Schotthoefer, A. M., Brown, W. M., Wheeler, E., Kitron, U. D. Rapid amplification of West Nile virus: the role of hatch-year birds. Vector Borne and Zoonotic Diseases 8(1), 57-68; 2008; 10.1089/vbz.2007.0123
46. Trawinski, P. R., Mackay, D. S. Meteorologically conditioned time-series predictions of West Nile virus vector mosquitoes. Vector Borne and Zoonotic Diseases 8(4), 505-522; 2008; 10.1089/vbz.2007.0202
47. Samia, N. I., Chan, K. S., Stenseth, N. C. A generalized threshold mixed model for analyzing nonnormal nonlinear time series, with application to plague in Kazakhstan. Biometrika 94(1), 101-118; 2007; 10.1093/biomet/asm006
48. de la Rocque, S., Tran, A. L., Etter, E., Vial, L., Hendrickx, G. Environmental changes, disease ecology and geographic information system-based tools for risk assessment. Veterinaria Italiana 43(3), 381-391; 2007
49. Hu WenBiao, Tong ShiLu, Mengersen, K., Oldenburg, B. Rainfall, mosquito density and the transmission of Ross River virus: a time-series forecasting model. Ecological Modelling 196(3/4), 505-514; 2006; 10.1016/j.ecolmodel.2006.02.028
50. Gordon, E. R., Curns, A. T., Krebs, J. W., Rupprecht, C. E., Real, L. A., Childs, J. E. Temporal dynamics of rabies in a wxildlife host and the risk of cross-species transmission. Epidemiology and Infection 132(3), 515-524; 2004; 10.1017/S0950268804002067
51. Baadsgaard, N. P., Hojsgaard, S., Grohn, Y. T., Schukken, Y. H. Forecasting clinical disease in pigs: comparing a naive and a Bayesian approach. Preventive Veterinary Medicine 64(2/4), 85-100; 2004; 10.1016/j.prevetmed.2004.06.001
52. Purse, B. V., Baylis, M., Tatem, A. J., Rogers, D. J., Mellor, P. S., Ham, M. van Chizov-Ginzburg, A., Braverman, Y. Predicting the risk of bluetongue through time: climate models of temporal patterns of outbreaks in Israel. Revue Scientifique et Technique - Office International des Epizooties 23(3), 761-775; 2004; 10.20506/rst.23.3.1515
53. Braverman, Y., Baylis, M., Tatem, A. J., Rogers, D. J., Mellor, P. S., Purse, B. V. What factors determine when epidemics occur in the Mediterranean? Prediction of disease risk through time by climate-driven models of the temporal distribution of outbreaks in Israel. Veterinaria Italiana 40(3), 235-242; 2004
54. Cruickshank, I., Wall, R. Population dynamics of the sheep blowfly Lucilia sericata: seasonal patterns and implications for control. Journal of Applied Ecology 39(3), 493-501; 2002; 10.1046/j.1365-2664.2002.00731.x
55. Ward, M. P. Seasonality of canine leptospirosis in the United States and Canada and its association with rainfall. Preventive Veterinary Medicine 56(3), 203-213; 2002; 10.1016/S0167-5877(02)00183-6
56. Nolan, T. J., Smith, G. Time series analysis of the prevalence of endoparasitic infections in cats and dogs presented to a veterinary teaching hospital. Veterinary Parasitology 59(2), 87-96; 1995; 10.1016/0304-4017(94)00742-U
57. Ernst, S., Cid, L., Martin, R., Thibaut, J. Temporal distribution of clinical parvovirosis in a canine hospital population of Valdivia, Chile (1981-1990). [Distribucion, temporal de la parvovirosis clinica en una población canina hospitalaria de Valdivia, Chile (1981-1990)]. Archivos de Medicina Veterinaria 24(2), 157-162; 1992
58. Goodall, E. A., McIlroy, S. G., McCracken, R. M., McLoughlin, E. M., Taylor, S. M. A mathematical forecasting model for the annual prevalence of fasciolosis. Agricultural Systems 36(2), 231-240; 1991; 10.1016/0308-521X(91)90026-7
59. McIlroy, S. G., Goodall, E. A., Stewart, D. A., McCracken, R. M. Investigating the epidemiology of disease using a computerised system for slaughterhouse condemnation data. Irish Veterinary Journal 43(2), 36-39; 1990
60. McIlroy, S. G., Goodall, E. A., Stewart, D. A., Taylor, S. M., McCracken, R. M. A computerised system for the accurate forecasting of the annual prevalence of fasciolosis. Preventive Veterinary Medicine 9(1), 27-35; 1990; 10.1016/0167-5877(90)90039-K

# Supplementary Figure 1

Demonstration code: Parvovirus time-series analysis in R

V Brookes, M Ward, R Iglesias

24 October 2019

# Time-series analysis of the parvovirus dataset

First, load the required packages (libraries).

To manipulate and plot the data:

library(ggplot2)
library(plyr)
library(dplyr)

##
## Attaching package: 'dplyr'

## The following objects are masked from 'package:plyr':
##
## arrange, count, desc, failwith, id, mutate, rename, summarise,
## summarize

## The following objects are masked from 'package:stats':
##
## filter, lag

## The following objects are masked from 'package:base':
##
## intersect, setdiff, setequal, union

library(lubridate)

##
## Attaching package: 'lubridate'

## The following object is masked from 'package:plyr':
##
## here

## The following object is masked from 'package:base':
##
## date

To conduct time-series analyses:

library(tseries)
library(vars)

## Loading required package: MASS

##
## Attaching package: 'MASS'

## The following object is masked from 'package:dplyr':
##
## select

## Loading required package: strucchange

## Loading required package: zoo

##
## Attaching package: 'zoo'

## The following objects are masked from 'package:base':
##
## as.Date, as.Date.numeric

## Loading required package: sandwich

## Loading required package: urca

## Loading required package: lmtest

library(forecast)

Now load the parvo dataset, and inspect it using the summary, str, head and tail functions:

ParvoD = read.csv('D:/Users/vbrookes/Dropbox (Sydney Uni)/Parvo_timeseries/Parvo_TS_clean.csv') # Note that you need to insert the location of the data in your computer system.
summary(ParvoD)

## Case.ID Case.Date Day Week
## Min. : 1 26/11/2012: 10 Min. : 1.0 Min. : 1.0
## 1st Qu.: 2882 17/01/2011: 9 1st Qu.: 418.0 1st Qu.: 59.0
## Median : 6990 5/11/2010 : 9 Median : 920.0 Median : 131.0
## Mean : 8220 12/05/2010: 8 Mean : 949.7 Mean : 135.3
## 3rd Qu.: 13372 19/05/2010: 8 3rd Qu.: 1459.0 3rd Qu.: 208.0
## Max. : 20245 25/11/2013: 8 Max. : 2218.0 Max. : 316.0
## (Other) :2935
## Cases Events
## Min. : 1.0 Min. : 1
## 1st Qu.: 1.0 1st Qu.: 1
## Median : 1.0 Median : 1
## Mean : 1.2 Mean : 1
## 3rd Qu.: 1.0 3rd Qu.: 1
## Max. : 13.0 Max. : 1
##

str(ParvoD)

## 'data.frame': 2987 obs. of 6 variables:
## $ Case.ID : int 1 693 695 697 46 632 3 4 701 5 ...
## $ Case.Date: Factor w/ 1423 levels "1/02/2010","1/02/2011",..: 1261 1261 1410 174 396 448 818 865 865 40 ...
## $ Day : int 1 1 4 7 12 13 20 21 21 27 ...
## $ Week : int 1 1 1 1 2 2 3 3 3 4 ...
## $ Cases : int 1 1 1 1 1 1 1 1 1 1 ...
## $ Events : int 1 1 1 1 1 1 1 1 1 1 ...

head(ParvoD)

## Case.ID Case.Date Day Week Cases Events
## 1 1 6/10/2009 1 1 1 1
## 2 693 6/10/2009 1 1 1 1
## 3 695 9/10/2009 4 1 1 1
## 4 697 12/10/2009 7 1 1 1
## 5 46 17/10/2009 12 2 1 1
## 6 632 18/10/2009 13 2 1 1

tail(ParvoD)

## Case.ID Case.Date Day Week Cases Events
## 2982 20244 26/10/2015 2212 315 1 1
## 2983 20150 26/10/2015 2212 315 1 1
## 2984 20243 27/10/2015 2213 316 1 1
## 2985 20183 28/10/2015 2214 316 1 1
## 2986 20234 30/10/2015 2216 316 1 1
## 2987 20245 1/11/2015 2218 316 2 1

Check for duplicated or missing data:

## Duplication
which(duplicated(ParvoD)) # anyDuplicated() does the same thing

## integer(0)

AllCompleteData = unique(ParvoD) # Can check that the dataset is the same length

#missing values in entire data set
ParvoD$complete<-complete.cases(ParvoD) # shows you if there are missing values in the row. If row is complete=TRUE
length(ParvoD$complete)

## [1] 2987

missingData <- ParvoD[which(ParvoD$complete == "FALSE"),]
missingData

## [1] Case.ID Case.Date Day Week Cases Events complete
## <0 rows> (or 0-length row.names)

Re-check your data, for example:

- How many observations are there?
- What are the data types of each column?
- Are they suitable for analysis?

Note that the column ‘Case.Date’ is a factor.

Convert it to date format, and find the minimum and maximum dates in the dataset. Also, add columns for week and month for aggregation purposes later in analysis:

length(ParvoD$complete) # total events

## [1] 2987

str(ParvoD)

## 'data.frame': 2987 obs. of 7 variables:
## $ Case.ID : int 1 693 695 697 46 632 3 4 701 5 ...
## $ Case.Date: Factor w/ 1423 levels "1/02/2010","1/02/2011",..: 1261 1261 1410 174 396 448 818 865 865 40 ...
## $ Day : int 1 1 4 7 12 13 20 21 21 27 ...
## $ Week : int 1 1 1 1 2 2 3 3 3 4 ...
## $ Cases : int 1 1 1 1 1 1 1 1 1 1 ...
## $ Events : int 1 1 1 1 1 1 1 1 1 1 ...
## $ complete : logi TRUE TRUE TRUE TRUE TRUE TRUE ...

sum(ParvoD$Cases) # total cases

## [1] 3584

sum(ParvoD$Events) # total events

## [1] 2987

## Save Case.Date to Date in a new column, with as.Date format
ParvoD$Date = as.Date(ParvoD$Case.Date, "%d/%m/%Y")

## Remove rows with no date (this should also have been detected in the previous chunk of code).
ParvoD <- subset(ParvoD,!(is.na(ParvoD["Date"]) ))

## Add columns that are by week or month
ParvoD$byWeek = cut(ParvoD$Date, breaks="1 week")
ParvoD$byMonth = cut(ParvoD$Date, breaks="1 month")

## find the minimum and maximum dates of observations
min(ParvoD$Date, na.rm = T)

## [1] "2009-10-06"

max(ParvoD$Date, na.rm = T)

## [1] "2015-11-01"

Summarise dataset by week and month, using ddply from the plyr package:

#### Aggregate data by week
ParvoW = ddply(ParvoD, c("byWeek"), summarise,
 Cases = sum(Cases),
 Events = sum(Events))
ParvoW$byWeek <- as.Date(ParvoW$byWeek, format = "%Y-%m-%d")
str(ParvoW)

## 'data.frame': 315 obs. of 3 variables:
## $ byWeek: Date, format: "2009-10-05" "2009-10-12" ...
## $ Cases : int 3 3 1 3 3 1 4 1 2 8 ...
## $ Events: int 3 3 1 3 3 1 4 1 2 5 ...

summary(ParvoW)

## byWeek Cases Events
## Min. : 2009-10-05 Min.: 1.00 Min. : 1.000
## 1st Qu.: 2011-04-14 1st Qu.: 5.00 1st Qu.: 5.000
## Median : 2012-10-15 Median: 9.00 Median :8.000
## Mean : 2012-10-15 Mean : 11.38 Mean : 9.483
## 3rd Qu.: 2014-04-17 3rd Qu.: 16.00 3rd Qu.: 13.000
## Max. : 2015-10-26 Max. : 45.00 Max. : 30.000

ParvoM = ddply(ParvoD, c("byMonth"), summarise,
 Cases = sum(Cases),
 Events = sum(Events))
ParvoM$byMonth <- as.Date(ParvoM$byMonth, format = "%Y-%m-%d")
str(ParvoM)

## 'data.frame': 74 obs. of 3 variables:
## $ byMonth: Date, format: "2009-10-01" "2009-11-01" ...
## $ Cases : int 9 9 21 49 66 66 104 114 62 51 ...
## $ Events : int 9 9 18 45 62 60 93 97 55 44 ...

summary(ParvoM)

## byMonth Cases Events
## Min. : 2009-10-01 Min. : 2.00 Min. : 1.00
## 1st Qu.: 2011-04-08 1st Qu.: 29.25 1st Qu.: 22.00
## Median : 2012-10-16 Median : 43.00 Median : 34.00
## Mean : 2012-10-15 Mean : 48.43 Mean :40.36
## 3rd Qu.: 2014-04-23 3rd Qu.: 66.00 3rd Qu.:54.50
## Max. : 2015-11-01 Max. :115.00 Max. : 97.00

Now create a dummy time series of weeks based on these time periods and merge with the time series. This identifies weeks (or months) with no cases. Note that some time series have many zero values at the time points.

# Create time sequence with 1 week intervals
data.length <- length(ParvoW$byWeek)
min.date = min(ParvoW$byWeek)
max.date = max(ParvoW$byWeek)

# Check length
length(ParvoW$Events)

## [1] 315

all.dates <- seq(min.date, max.date, by="week")
all.dates.frame <- data.frame(list(byWeek=all.dates)) # Make it into a data frame so that we can merge it

# Merge data with weekly Parvo data
ParvoW <- merge(all.dates.frame, ParvoW, all = T)
# Change NAs to 0
ParvoW$Cases[is.na(ParvoW$Cases)] <- 0
ParvoW$Events[is.na(ParvoW$Events)] <- 0

# summary
summary(ParvoW)

## byWeek Cases Events
## Min. : 2009-10-05 Min. : 0.00 Min. : 0.000
## 1st Qu.: 2011-04-11 1st Qu.: 5.00 1st Qu.: 4.000
## Median : 2012-10-15 Median : 9.00 Median : 8.000
## Mean : 2012-10-15 Mean : 11.31 Mean : 9.423
## 3rd Qu.: 2014-04-21 3rd Qu.: 16.00 3rd Qu.: 13.000
## Max. : 2015-10-26 Max. : 45.00 Max. : 30.000

# check structure
str(ParvoW)

## 'data.frame': 317 obs. of 3 variables:
## $ byWeek: Date, format: "2009-10-05" "2009-10-12" ...
## $ Cases : num 3 3 1 3 0 3 1 4 1 2 ...
## $ Events: num 3 3 1 3 0 3 1 4 1 2 ...

# Check length
length(ParvoW$Events) # Note that in this dataset, there must be 2 weeks with 0 cases (because this is two weeks longer).

## [1] 317

We can also create a monthly times series:

# Create time sequence with 1 month intervals
data.lengthM <- length(ParvoM$byMonth)
min.dateM = min(ParvoM$byMonth)
max.dateM = max(ParvoM$byMonth)

# Check length
length(ParvoM$Cases)

## [1] 74

all.dates <- seq(min.dateM, max.dateM, by="month")
all.dates.frame <- data.frame(list(byMonth=all.dates)) # Make it into a data frame so that we can merge it

# Merge data with monthly Parvo data
ParvoM <- merge(all.dates.frame, ParvoM, all = T)
# Change NAs to 0
ParvoM$Cases[which(is.na(ParvoM$Cases))] <- 0
ParvoM$Events[which(is.na(ParvoM$Events))] <- 0

# summary
summary(ParvoM)

## byMonth Cases Events
## Min. : 2009-10-01 Min. : 2.00 Min. : 1.00
## 1st Qu.: 2011-04-08 1st Qu.: 29.25 1st Qu.: 22.00
## Median : 2012-10-16 Median : 43.00 Median : 34.00
## Mean : 2012-10-15 Mean : 48.43 Mean : 40.36
## 3rd Qu.: 2014-04-23 3rd Qu.: 66.00 3rd Qu.: 54.50
## Max. : 2015-11-01 Max. : 115.00 Max. : 97.00

# check structure
str(ParvoM)

## 'data.frame': 74 obs. of 3 variables:
## $ byMonth: Date, format: "2009-10-01" "2009-11-01" ...
## $ Cases : num 9 9 21 49 66 66 104 114 62 51 ...
## $ Events : num 9 9 18 45 62 60 93 97 55 44 ...

write.csv(ParvoM, 'D:/Users/vbrookes/Dropbox (Sydney Uni)/Parvo_timeseries/Parvo_Month.csv')
# Check length
length(ParvoM$Events)

## [1] 74

## Exploratory analysis

### Plot the weekly time-series

Here, we use ggplot. The following code is used to make the plots attractive and is a useful ‘publication-ready’ theme:

themeVB = theme(axis.text.x = element_text(colour = "black", angle = 90, hjust=1,vjust=0.5, size = 11),
 axis.line = element_line(colour = "black"),
 axis.text.y = element_text(colour = "black"),
 axis.ticks = element_line(colour = "black"),
 axis.title.y = element_text(vjust=1.5),
 panel.grid.major = element_line(colour = "grey93"),
 panel.grid.minor = element_line(colour = "white"),
 panel.background = element_blank(),
 legend.background = element_rect(colour = NA),
 legend.key = element_rect(fill = 'transparent'))

The blue line is the smoothed number of cases with a shaded 95% confidence interval. You can change the smoothing method (here, we have used ‘auto’).

There seems to be an overall decreasing trend in the number of weekly reported cases. Seasonality is also possible but difficult to determine using this plot.

ggplot(ParvoW, aes(x = byWeek, y = Events, group = 1)) +
 geom_bar(stat="identity", width = 0.5, colour = "black") +
 stat_smooth(aes(y = Events), method='auto', level=0.95) +
 themeVB +
 scale_x_date() +
 xlab("Year") +
 ylab("Cases")

## `geom_smooth()` using method = 'loess' and formula 'y ~ x'


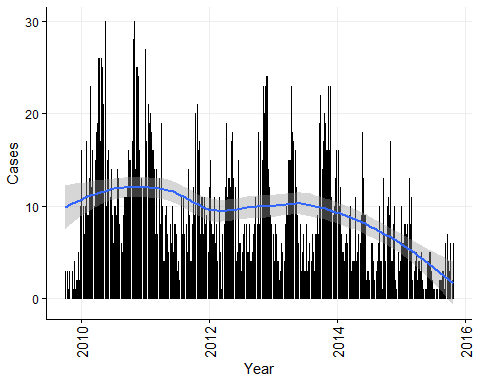


ggplot(ParvoM, aes(x = byMonth, y = Events, group = 1)) +
 geom_bar(stat="identity", width = 0.5, colour = "black") +
 stat_smooth(aes(y = Events), method='auto', level=0.95) +
 themeVB +
 scale_x_date() +
 xlab("Year") +
 ylab("Cases")

## `geom_smooth()` using method = 'loess' and formula 'y ~ x'


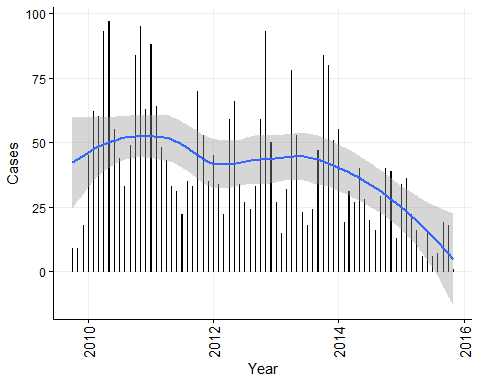


### Convert the data to an R recognised time series

Here, we use the ts function from the stats package.

frequency = 52 is specified because there are 52 weeks (sort of) in a year.

Parvo_ts_data <- ts(ParvoW$Events, start = c(2009, 40), frequency =52)

Parvo_ts_dataM <- ts(ParvoM$Events, start = c(2009, 10), frequency =12)

There are a decreasing number of events over time.

We can quantify this trend using linear regression with predictor variables for trend (by week or month per year) and seasonality (week or month).

The weekly change in the number of cases is -1.22 events/week/year (95% CI -1.57 - -0.87), confirming that the long-term trend is decreasing reported cases.

# Weekly ts dataset
fit_Parvo_data = ts_all_model <- lm(Parvo_ts_data ~ time(Parvo_ts_data) + factor(cycle(Parvo_ts_data)))
summary(fit_Parvo_data)

##
## Call:
## lm(formula = Parvo_ts_data ~ time(Parvo_ts_data) + factor(cycle(Parvo_ts_data)))
##
## Residuals:
## Min 1Q Median 3Q Max
## -17.2400 -2.8324 -0.1095 2.7762 14.9429
##
## Coefficients:
## Estimate Std. Error t value Pr(>|t|)
## (Intercept) 2474.6667 360.1895 6.870 4.59e-11 ***
## time(Parvo_ts_data) -1.2229 0.1790 -6.833 5.74e-11 ***
## factor(cycle(Parvo_ts_data))2 -1.6432 3.2057 -0.513 0.60868
## factor(cycle(Parvo_ts_data))3 -3.6196 3.2057 -1.129 0.25988
## factor(cycle(Parvo_ts_data))4 -3.0961 3.2057 -0.966 0.33503
## factor(cycle(Parvo_ts_data))5 -2.4059 3.2058 -0.751 0.45362
## factor(cycle(Parvo_ts_data))6 -5.7158 3.2058 -1.783 0.07574 .
## factor(cycle(Parvo_ts_data))7 -4.8589 3.2058 -1.516 0.13080
## factor(cycle(Parvo_ts_data))8 -2.5021 3.2058 -0.780 0.43581
## factor(cycle(Parvo_ts_data))9 -5.1452 3.2058 -1.605 0.10970
## factor(cycle(Parvo_ts_data))10 -4.1217 3.2059 -1.286 0.19969
## factor(cycle(Parvo_ts_data))11 -7.2648 3.2059 -2.266 0.02426 *
## factor(cycle(Parvo_ts_data))12 -3.7413 3.2059 -1.167 0.24427
## factor(cycle(Parvo_ts_data))13 -5.0511 3.2060 -1.576 0.11633
## factor(cycle(Parvo_ts_data))14 -1.0276 3.2060 -0.321 0.74882
## factor(cycle(Parvo_ts_data))15 -0.6708 3.2061 -0.209 0.83444
## factor(cycle(Parvo_ts_data))16 -1.6473 3.2061 -0.514 0.60784
## factor(cycle(Parvo_ts_data))17 -1.1237 3.2062 -0.350 0.72625
## factor(cycle(Parvo_ts_data))18 -0.9336 3.2063 -0.291 0.77115
## factor(cycle(Parvo_ts_data))19 -1.5767 3.2063 -0.492 0.62331
## factor(cycle(Parvo_ts_data))20 -1.2199 3.2064 -0.380 0.70392
## factor(cycle(Parvo_ts_data))21 -4.8630 3.2065 -1.517 0.13056
## factor(cycle(Parvo_ts_data))22 -3.5062 3.2065 -1.093 0.27520
## factor(cycle(Parvo_ts_data))23 -3.9826 3.2066 -1.242 0.21534
## factor(cycle(Parvo_ts_data))24 -6.9591 3.2067 -2.170 0.03088 *
## factor(cycle(Parvo_ts_data))25 -4.9356 3.2068 -1.539 0.12497
## factor(cycle(Parvo_ts_data))26 -7.2454 3.2069 -2.259 0.02468 *
## factor(cycle(Parvo_ts_data))27 -9.7219 3.2070 -3.031 0.00268 **
## factor(cycle(Parvo_ts_data))28 -8.0317 3.2071 -2.504 0.01287 *
## factor(cycle(Parvo_ts_data))29 -8.3415 3.2072 -2.601 0.00982 **
## factor(cycle(Parvo_ts_data))30 -5.6514 3.2073 -1.762 0.07922 .
## factor(cycle(Parvo_ts_data))31 -6.4612 3.2074 -2.014 0.04497 *
## factor(cycle(Parvo_ts_data))32 -7.9377 3.2075 -2.475 0.01396 *
## factor(cycle(Parvo_ts_data))33 -7.9141 3.2076 -2.467 0.01425 *
## factor(cycle(Parvo_ts_data))34 -7.5573 3.2077 -2.356 0.01921 *
## factor(cycle(Parvo_ts_data))35 -7.8671 3.2079 -2.452 0.01484 *
## factor(cycle(Parvo_ts_data))36 -4.5103 3.2080 -1.406 0.16092
## factor(cycle(Parvo_ts_data))37 -5.9867 3.2081 -1.866 0.06313 .
## factor(cycle(Parvo_ts_data))38 -6.4632 3.2083 -2.015 0.04496 *
## factor(cycle(Parvo_ts_data))39 -0.4397 3.2084 -0.137 0.89110
## factor(cycle(Parvo_ts_data))40 -3.2181 3.0894 -1.042 0.29853
## factor(cycle(Parvo_ts_data))41 -3.9089 3.0895 -1.265 0.20691
## factor(cycle(Parvo_ts_data))42 -2.0282 3.0895 -0.656 0.51209
## factor(cycle(Parvo_ts_data))43 1.2810 3.0896 0.415 0.67875
## factor(cycle(Parvo_ts_data))44 0.3045 3.0897 0.099 0.92156
## factor(cycle(Parvo_ts_data))45 1.1452 3.2058 0.357 0.72121
## factor(cycle(Parvo_ts_data))46 -0.4979 3.2058 -0.155 0.87668
## factor(cycle(Parvo_ts_data))47 0.5256 3.2058 0.164 0.86990
## factor(cycle(Parvo_ts_data))48 -0.2842 3.2058 -0.089 0.92941
## factor(cycle(Parvo_ts_data))49 -5.0941 3.2058 -1.589 0.11325
## factor(cycle(Parvo_ts_data))50 -5.7372 3.2057 -1.790 0.07465 .
## factor(cycle(Parvo_ts_data))51 -5.8804 3.2057 -1.834 0.06773 .
## factor(cycle(Parvo_ts_data))52 -5.1902 3.2057 -1.619 0.10663
## ---
## Signif. codes: 0 '***' 0.001 '**' 0.01 '*' 0.05 '.' 0.1 ' ' 1
##
## Residual standard error: 5.552 on 264 degrees of freedom
## Multiple R-squared: 0.3429, Adjusted R-squared: 0.2135
## F-statistic: 2.65 on 52 and 264 DF, p-value: 2.098e-07

confint(fit_Parvo_data)

## 2.5 % 97.5 %
## (Intercept) 1765.456986 3183.8763478
## time(Parvo_ts_data) -1.575252 -0.8704618
## factor(cycle(Parvo_ts_data))2 -7.955196 4.6688959
## factor(cycle(Parvo_ts_data))3 -9.931691 2.6924233
## factor(cycle(Parvo_ts_data))4 -9.408192 3.2159579
## factor(cycle(Parvo_ts_data))5 -8.718035 3.9061665
## factor(cycle(Parvo_ts_data))6 -12.027884 0.5963824
## factor(cycle(Parvo_ts_data))7 -11.171074 1.4532723
## factor(cycle(Parvo_ts_data))8 -8.814272 3.8101694
## factor(cycle(Parvo_ts_data))9 -11.457477 1.1670738
## factor(cycle(Parvo_ts_data))10 -10.434022 2.1906521
## factor(cycle(Parvo_ts_data))11 -13.577241 -0.9524290
## factor(cycle(Parvo_ts_data))12 -10.053801 2.5711639
## factor(cycle(Parvo_ts_data))13 -11.363702 1.2614307
## factor(cycle(Parvo_ts_data))14 -7.340276 5.2850381
## factor(cycle(Parvo_ts_data))15 -6.983525 5.6419862
## factor(cycle(Parvo_ts_data))16 -7.960114 4.6656081
## factor(cycle(Parvo_ts_data))17 -7.436710 5.1892374
## factor(cycle(Parvo_ts_data))18 -7.246647 5.3795406
## factor(cycle(Parvo_ts_data))19 -7.889924 4.7365177
## factor(cycle(Parvo_ts_data))20 -7.533209 5.0935021
## factor(cycle(Parvo_ts_data))21 -11.176501 1.4504937
## factor(cycle(Parvo_ts_data))22 -9.819800 2.8074927
## factor(cycle(Parvo_ts_data))23 -10.296440 2.3311655
## factor(cycle(Parvo_ts_data))24 -13.273087 -0.6451543
## factor(cycle(Parvo_ts_data))25 -11.249742 1.3785331
## factor(cycle(Parvo_ts_data))26 -13.559737 -0.9311056
## factor(cycle(Parvo_ts_data))27 -16.036406 -3.4074036
## factor(cycle(Parvo_ts_data))28 -14.346416 -1.7170277
## factor(cycle(Parvo_ts_data))29 -14.656432 -2.0266446
## factor(cycle(Parvo_ts_data))30 -11.966456 0.6637458
## factor(cycle(Parvo_ts_data))31 -12.776488 -0.1458565
## factor(cycle(Parvo_ts_data))32 -14.253193 -1.6221182
## factor(cycle(Parvo_ts_data))33 -14.229906 -1.5983727
## factor(cycle(Parvo_ts_data))34 -13.873292 -1.2412865
## factor(cycle(Parvo_ts_data))35 -14.183353 -1.5508598
## factor(cycle(Parvo_ts_data))36 -10.826754 1.8062409
## factor(cycle(Parvo_ts_data))37 -12.303495 0.3300155
## factor(cycle(Parvo_ts_data))38 -12.780244 -0.1462027
## factor(cycle(Parvo_ts_data))39 -6.757000 5.8775864
## factor(cycle(Parvo_ts_data))40 -9.301169 2.8649790
## factor(cycle(Parvo_ts_data))41 -9.992041 2.1743117
## factor(cycle(Parvo_ts_data))42 -8.111491 4.0550805
## factor(cycle(Parvo_ts_data))43 -4.802377 7.3644283
## factor(cycle(Parvo_ts_data))44 -5.778985 6.3880693
## factor(cycle(Parvo_ts_data))45 -5.167074 7.4574767
## factor(cycle(Parvo_ts_data))46 -6.810169 5.8142719
## factor(cycle(Parvo_ts_data))47 -5.786606 6.8377411
## factor(cycle(Parvo_ts_data))48 -6.596382 6.0278843
## factor(cycle(Parvo_ts_data))49 -11.406167 1.2180347
## factor(cycle(Parvo_ts_data))50 -12.049291 0.5748590
## factor(cycle(Parvo_ts_data))51 -12.192423 0.4316907
## factor(cycle(Parvo_ts_data))52 -11.502229 1.1218629

# Monthly ts dataset
fit_Parvo_dataM = ts_all_model <- lm(Parvo_ts_dataM ~ time(Parvo_ts_dataM) + factor(cycle(Parvo_ts_dataM)))
summary(fit_Parvo_dataM)

##
## Call:
## lm(formula = Parvo_ts_dataM ~ time(Parvo_ts_dataM) + factor(cycle(Parvo_ts_dataM)))
##
## Residuals:
## Min 1Q Median 3Q Max
## -61.136 -8.408 -0.440 9.684 40.143
##
## Coefficients:
## Estimate Std. Error t value Pr(>|t|)
## (Intercept) 11640.477 2758.508 4.220 8.25e-05 ***
## time(Parvo_ts_dataM) -5.760 1.371 -4.202 8.77e-05 ***
## factor(cycle(Parvo_ts_dataM))2 -10.187 12.028 -0.847 0.4004
## factor(cycle(Parvo_ts_dataM))3 -12.207 12.030 -1.015 0.3143
## factor(cycle(Parvo_ts_dataM))4 5.107 12.033 0.424 0.6728
## factor(cycle(Parvo_ts_dataM))5 2.087 12.036 0.173 0.8629
## factor(cycle(Parvo_ts_dataM))6 -15.433 12.041 -1.282 0.2048
## factor(cycle(Parvo_ts_dataM))7 -23.287 12.047 -1.933 0.0579 .
## factor(cycle(Parvo_ts_dataM))8 -22.473 12.054 -1.864 0.0671 .
## factor(cycle(Parvo_ts_dataM))9 -10.160 12.062 -0.842 0.4029
## factor(cycle(Parvo_ts_dataM))10 4.440 11.595 0.383 0.7031
## factor(cycle(Parvo_ts_dataM))11 5.777 11.599 0.498 0.6202
## factor(cycle(Parvo_ts_dataM))12 -11.147 12.028 -0.927 0.3577
## ---
## Signif. codes: 0 '***' 0.001 '**' 0.01 '*' 0.05 '.' 0.1 ' ' 1
##
## Residual standard error: 20.83 on 61 degrees of freedom
## Multiple R-squared: 0.3766, Adjusted R-squared: 0.254
## F-statistic: 3.071 on 12 and 61 DF, p-value: 0.00193

confint(fit_Parvo_dataM)

## 2.5 % 97.5 %
## (Intercept) 6124.499204 17156.4553418
## time(Parvo_ts_dataM) -8.500586 -3.0188946
## factor(cycle(Parvo_ts_dataM))2 -34.238596 13.8652191
## factor(cycle(Parvo_ts_dataM))3 -36.261871 11.8484507
## factor(cycle(Parvo_ts_dataM))4 -18.953980 29.1671835
## factor(cycle(Parvo_ts_dataM))5 -21.981589 26.1547494
## factor(cycle(Parvo_ts_dataM))6 -39.511363 8.6444796
## factor(cycle(Parvo_ts_dataM))7 -47.376631 0.8030383
## factor(cycle(Parvo_ts_dataM))8 -46.577392 1.6304221
## factor(cycle(Parvo_ts_dataM))9 -34.280307 13.9599606
## factor(cycle(Parvo_ts_dataM))10 -18.746145 27.6260150
## factor(cycle(Parvo_ts_dataM))11 -17.416897 28.9710099
## factor(cycle(Parvo_ts_dataM))12 -35.198552 12.9052624

### Decompose the time-series

The observed data is composed of trend, cycle, seasonal and random variation.

The function decompose is from the base package stats, and uses moving averages to smooth the time-series. We also demonstrate the function ‘stl’ from the stats package which uses loess smoothing.

The data are illustrated as follows:

Observed − the actual data.

Trend − the overall upward or downward movement of the data points. It is not necessarily linear.

Seasonal − any monthly/yearly pattern of the data points.

Random (remainder) − unexplainable part of the data.

# Weekly ts dataset
Decompose_ts <- decompose(Parvo_ts_data)
summary(Decompose_ts)

## Length Class Mode
## x 317 ts numeric
## seasonal 317 ts numeric
## trend 317 ts numeric
## random 317 ts numeric
## figure 52 -none- numeric
## type 1 -none- character

plot(Decompose_ts)


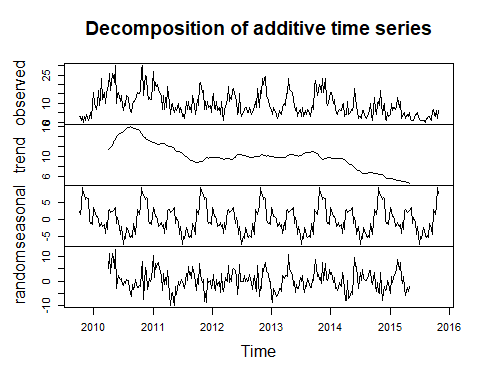


plot(Decompose_ts$trend + Decompose_ts$random, ylab = "trend + random components") # can also plot combinations


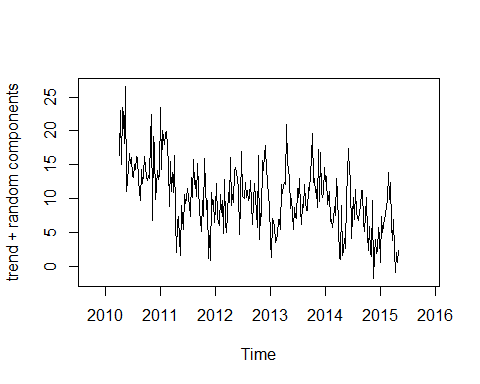


Decompose_ts_loess <- stl(Parvo_ts_data, s.window="periodic")
plot(Decompose_ts_loess)


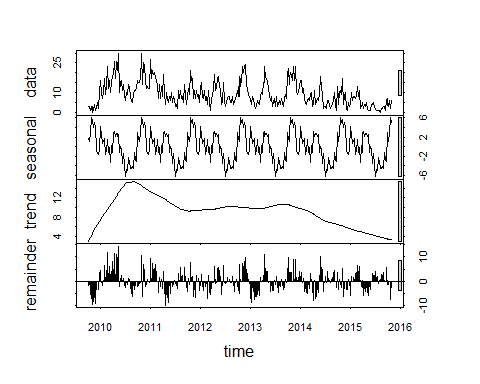


# Monthly ts dataset
Decompose_tsM <- decompose(Parvo_ts_dataM)
summary(Decompose_tsM)

## Length Class Mode
## x 74 ts numeric
## seasonal 74 ts numeric
## trend 74 ts numeric
## random 74 ts numeric
## figure 12 -none- numeric
## type 1 -none- character

plot(Decompose_tsM)


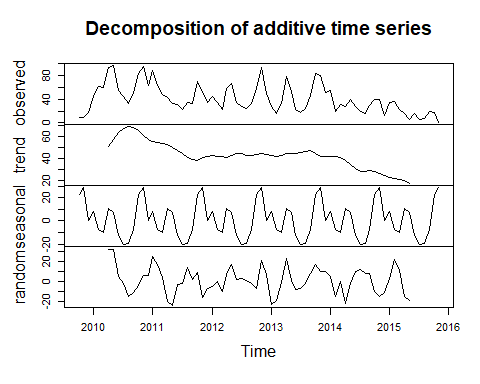


Decompose_ts_loessM <- stl(Parvo_ts_dataM, s.window="periodic")
plot(Decompose_ts_loessM)


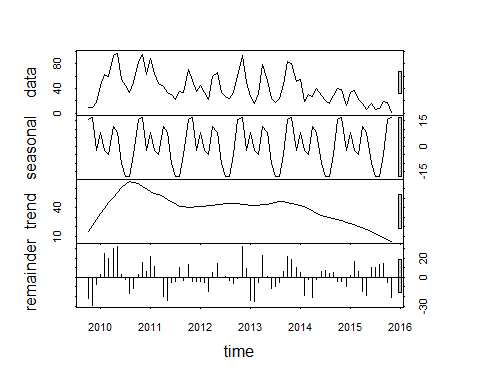


## There looks like seasonality. Add a week and month column to ParvoW to investigate further
ParvoW$Year = year(ParvoW$byWeek)
ParvoW$Week = week(ParvoW$byWeek)
ParvoW$Month = month(ParvoW$byWeek)

ggplot(ParvoW, aes(y=Events, x = Week)) + geom_boxplot(aes(group=Week), fill = 'grey90') + themeVB


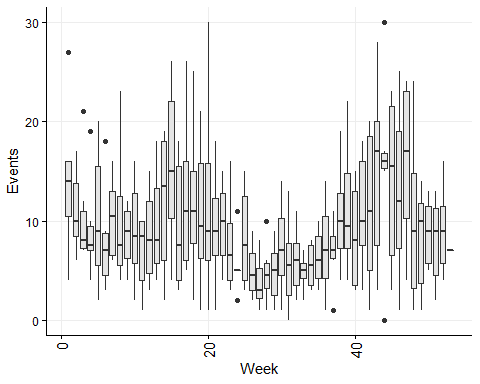


ggplot(ParvoW, aes(y=Events, x = Month)) + geom_boxplot(aes(group=Month), fill = 'grey90') + themeVB


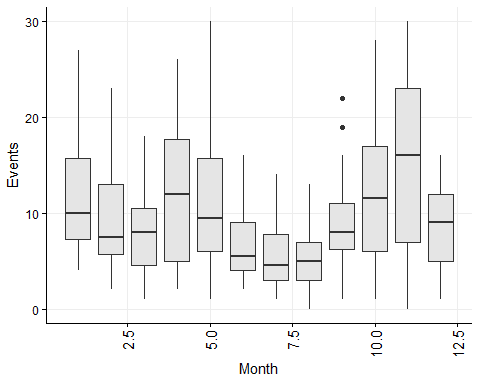


ggplot(ParvoW, aes(y=Events, x = Year)) + geom_boxplot(aes(group=Year), fill = 'grey90') + themeVB


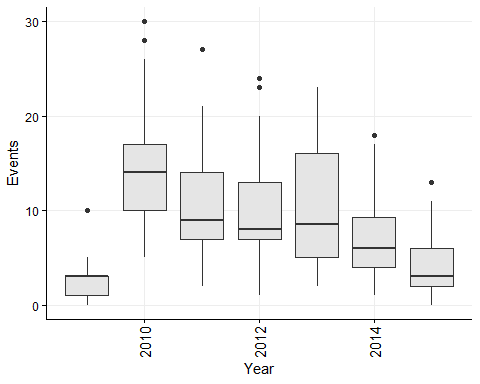


Given the apparent seasonality in both the weekly and monthly time-series, it is interesting to visually compare the two seasonal plots from the ‘decomposed’ data.

### Compare the seasonality plots

plot(Decompose_tsM$seasonal)
lines(Decompose_ts$seasonal, col = 'red')


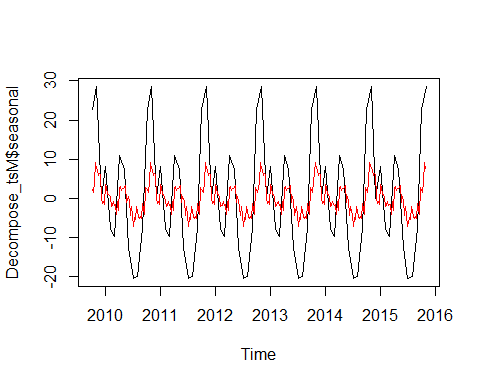


# Time-series analysis using autoregressive models

In this example, we fit an ARIMA model. ARIMA is an abbreviation for ‘AutoRegressive Integrated Moving Average’.

Auto Regressive (AR) terms refer to the number of lagged values in the model. In the non-seasonal part of the model, the order of lagged values is termed ‘p’, and in the seasonal part of the model the order of lagged values is termed ‘P’.

Moving Average (MA) terms refer to the number of lagged errors in the model. In the non-seasonal part of the model, the order of lagged errors is termed ‘q’, and in the seasonal part of the model the order of lagged errors is termed ‘Q’.

Integration (I) terms refer to the number of differences used to make the time series stationary. In the non-seasonal part of the model, the order of differences is termed ‘d’, and in the seasonal part of the model the order of differences is termed ‘D’.

Overall, the model includes the following variables:

ARIMA: (p, d, q) (P, D, Q)m

where m refers to the number of observations in a seasonal cycle.

#### Assumptions of ARIMA models

Data should be stationary − this means that the properties of the series do not depend on the time when it is captured, i.e. trend and seasonality are removed to leave ‘white-noise’. Note that a series with cyclic behaviour can also be considered stationary because cyclic behaviours have unpredictable wavelengths. A stationary series will have constant variance (see below).

#### Steps to be followed for ARIMA modeling:

1. Exploratory analysis − to determine values for (p, d, q) (P, D, Q)m
2. Fit the model
3. Diagnostic measures to assess model fit

## Exploratory analysis for ARIMA modelling

We start by assessing the need for differencing to determine values for d and D.

### Tests for stationarity (is there a need to ‘first difference’ the data?)

It is important that the data to which the ARIMA model is fitted are stationary. Observations in a non-stationary time series demonstrate structure that is dependent on the time index. Inducing stationarity means removing trend, seasonality and possibly cyclicity (if it is predictable).

There are several methods to check for stationarity:

1. View plots of time-series for obvious trend, or examine the autocorrelation function (ACF) plots. There should be little autocorrelation in stationary data, so an ACF plot should decrease to zero rapidly, and stay at zero.
2. Summary statistics. Check for significant differences in mean and variance between sections of data. We do not demonstrate this in the current study because these are standard statistical tests.
3. Statistical tests. These tests determine whether the expectations of stationarity are met or violated.

In our example dataset, we initially re-examine the time series and examine an ACF plot of the weekly and monthly events. We observe decreasing trend and the ACF plot demonstrates that there is autocorrelation for 10 and 2 lags in the weekly and monthly series, respectively. These time series are not stationary.

We then run ndiffs from the forecast package to assess how many first differences are needed to induce stationarity. ndiffs suggests one first order difference for each time series. After differencing the data, the time-series plots appears level (no trend) and the ACF plots show limited autocorrelation (one lag) in the first few lags.

## Tests for stationarity
# Weekly data
Parvo_ts_data %>% ggtsdisplay(theme = themeVB)


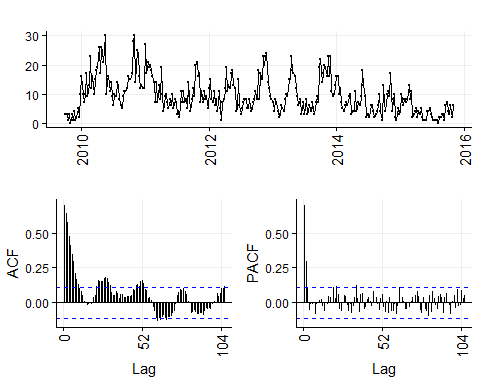


ndiffs(Parvo_ts_data) # 1 difference estimated to induce stationarity

## [1] 1

nsdiffs(Parvo_ts_data) # No differencing required to induce stationarity

## [1] 0

Parvo_ts_data %>% diff() %>% ggtsdisplay(theme = themeVB) # Assess differenced ts and ACF and PACF plots.


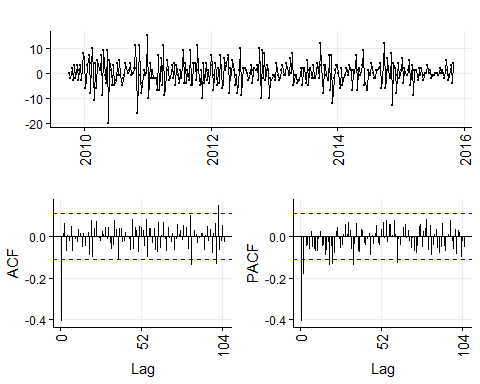


# Monthly data
Parvo_ts_dataM %>% ggtsdisplay(theme = themeVB)


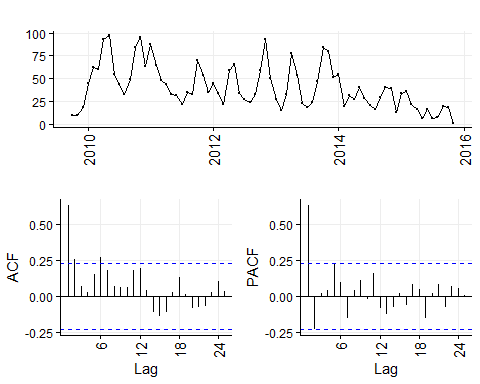


ndiffs(Parvo_ts_dataM) # 1 difference estimated to induce stationarity

## [1] 1

nsdiffs(Parvo_ts_dataM) # No differencing required to induce stationarity

## [1] 0

Parvo_ts_dataM %>% diff() %>% ggtsdisplay(theme = themeVB) # Assess differenced ts and ACF and PACF plots.


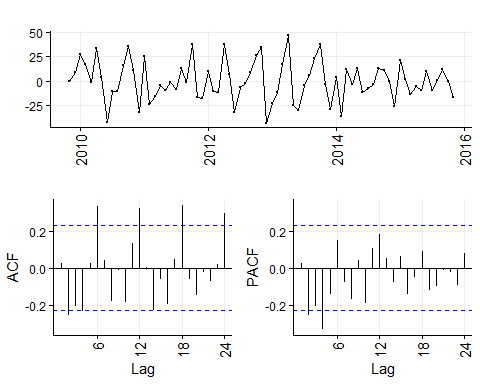


We further check for stationarity in the time series using statistical tests. [This website](https://rpubs.com/richkt/269797) is a useful resource.

Frequently used tests include:

1. Ljung-Box test for independence (Null hypothesis is of time independence in a given period of lags. A low P value suggests that the data are not consistent with independence);
2. Augmented Dickey-Fuller (ADF) t-statistic test for unit root (note that a series with a trend line will have a unit root and result in a large P value, i.e. the null hypothesis is that there is a unit root present); and
3. Kwiatkowski-Phillips-Schmidt-Shin (KPSS) for level or trend stationarity (null hypothesis is that the time-series is stationary).

## Weekly time-series tests for stationarity
# 1.
Box.test(Parvo_ts_data, lag = 52, type="Ljung-Box")

##
## Box-Ljung test
##
## data: Parvo_ts_data
## X-squared = 764.84, df = 52, p-value < 2.2e-16

# 2.
adf.test(Parvo_ts_data, alternative = "stationary", k = 104)

##
## Augmented Dickey-Fuller Test
##
## data: Parvo_ts_data
## Dickey-Fuller = -1.3184, Lag order = 104, p-value = 0.8638
## alternative hypothesis: stationary

# 3.
kpss.test(Parvo_ts_data, null = 'Trend') # The null hypothesis is a time trend with stationary error.

## Warning in kpss.test(Parvo_ts_data, null = "Trend"): p-value greater than
## printed p-value

##
## KPSS Test for Trend Stationarity
##
## data: Parvo_ts_data
## KPSS Trend = 0.10897, Truncation lag parameter = 5, p-value = 0.1

kpss.test(Parvo_ts_data, null = 'Level') # The null hypothesis is that the series is white noise.

## Warning in kpss.test(Parvo_ts_data, null = "Level"): p-value smaller than
## printed p-value

##
## KPSS Test for Level Stationarity
##
## data: Parvo_ts_data
## KPSS Level = 1.1935, Truncation lag parameter = 5, p-value = 0.01

## Monthly time-series tests for stationarity
# 1.
Box.test(Parvo_ts_dataM, lag = 12, type="Ljung-Box")

##
## Box-Ljung test
##
## data: Parvo_ts_dataM
## X-squared = 53.87, df = 12, p-value = 2.883e-07

# 2.
adf.test(Parvo_ts_dataM, alternative = "stationary", k = 24)

##
## Augmented Dickey-Fuller Test
##
## data: Parvo_ts_dataM
## Dickey-Fuller = -0.84262, Lag order = 24, p-value = 0.9536
## alternative hypothesis: stationary

# 3.
kpss.test(Parvo_ts_dataM, null = 'Trend') # The null hypothesis is a time trend with stationary error.

## Warning in kpss.test(Parvo_ts_dataM, null = "Trend"): p-value greater than
## printed p-value

##
## KPSS Test for Trend Stationarity
##
## data: Parvo_ts_dataM
## KPSS Trend = 0.086265, Truncation lag parameter = 3, p-value = 0.1

kpss.test(Parvo_ts_dataM, null = 'Level') # The null hypothesis is that the series is white noise.

##
## KPSS Test for Level Stationarity
##
## data: Parvo_ts_dataM
## KPSS Level = 0.71758, Truncation lag parameter = 3, p-value =
## 0.01195

We difference the time-series, then test for stationarity again:

## Weekly time-series tests for stationarity
# 1.
Box.test(diff(Parvo_ts_data), lag=52, type="Ljung-Box")

##
## Box-Ljung test
##
## data: diff(Parvo_ts_data)
## X-squared = 96.191, df = 52, p-value = 0.0001887

# 2.
adf.test(diff(Parvo_ts_data), alternative = "stationary", k = 104)

##
## Augmented Dickey-Fuller Test
##
## data: diff(Parvo_ts_data)
## Dickey-Fuller = -2.352, Lag order = 104, p-value = 0.428
## alternative hypothesis: stationary

# 3.
kpss.test(diff(Parvo_ts_data), null = 'Trend') # The null hypothesis is a time trend with stationary error.

## Warning in kpss.test(diff(Parvo_ts_data), null = "Trend"): p-value greater
## than printed p-value

##
## KPSS Test for Trend Stationarity
##
## data: diff(Parvo_ts_data)
## KPSS Trend = 0.016653, Truncation lag parameter = 5, p-value = 0.1

kpss.test(diff(Parvo_ts_data), null = 'Level') # The null hypothesis is that the series is white noise.

## Warning in kpss.test(diff(Parvo_ts_data), null = "Level"): p-value greater
## than printed p-value

##
## KPSS Test for Level Stationarity
##
## data: diff(Parvo_ts_data)
## KPSS Level = 0.025954, Truncation lag parameter = 5, p-value = 0.1

## Monthly time-series tests for stationarity
# 1.
Box.test(diff(Parvo_ts_dataM), lag=12, type="Ljung-Box")

##
## Box-Ljung test
##
## data: diff(Parvo_ts_dataM)
## X-squared = 38.561, df = 12, p-value = 0.0001243

# 2.
adf.test(diff(Parvo_ts_dataM), alternative = "stationary", k = 24)

##
## Augmented Dickey-Fuller Test
##
## data: diff(Parvo_ts_dataM)
## Dickey-Fuller = -1.9387, Lag order = 24, p-value = 0.6003
## alternative hypothesis: stationary

# 3.
kpss.test(diff(Parvo_ts_dataM), null = 'Trend') # The null hypothesis is a time trend with stationary error.

## Warning in kpss.test(diff(Parvo_ts_dataM), null = "Trend"): p-value greater
## than printed p-value

##
## KPSS Test for Trend Stationarity
##
## data: diff(Parvo_ts_dataM)
## KPSS Trend = 0.032465, Truncation lag parameter = 3, p-value = 0.1

kpss.test(diff(Parvo_ts_dataM), null = 'Level') # The null hypothesis is that the series is white noise.

## Warning in kpss.test(diff(Parvo_ts_dataM), null = "Level"): p-value greater
## than printed p-value

##
## KPSS Test for Level Stationarity
##
## data: diff(Parvo_ts_dataM)
## KPSS Level = 0.091647, Truncation lag parameter = 3, p-value = 0.1

### Interpreting ACF and PACF plots to determine p, d, P and D

From our tests of stationarity, we know that ‘d’ in the non-seasonal part of the ARIMA model is likely to be 1, and ‘D’ in the seasonal part of the model is likely to be zero.

We now use the ACF and PACF plots of the differenced data to estimate the p and q values in the non-seasonal section of the ARIMA model:

## Examine ACF and PACF plots of differenced time-series again to estimate p, P, q and Q.
# Weekly data
Parvo_ts_data %>% diff() %>% ggtsdisplay(theme = themeVB) # Assess differenced ts and ACF and PACF plots.


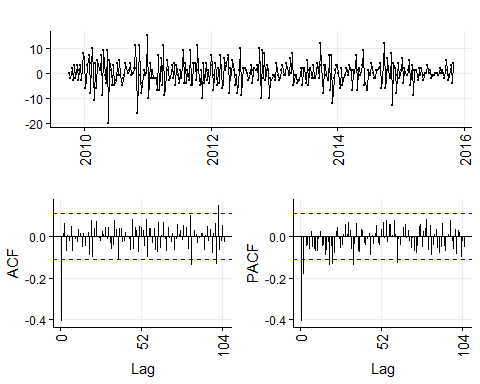


# Monthly data
Parvo_ts_dataM %>% diff() %>% ggtsdisplay(theme = themeVB) # Assess differenced ts and ACF and PACF plots.


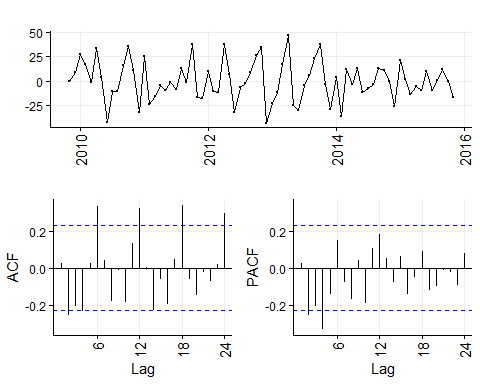


The ACF plot of the weekly time series has a fast initial decay with only the first lag significant. This indicates MA(1) for the weekly ARIMA model. The ACF plot of the monthly time series has limited autocorrelation at 2 lags. This could indicate AR(0−2) for the monthly ARIMA model.

The PACF plot for the weekly data has a fast decay with partial autocorrelation in the first two lags significant. This suggests AR(2). The PACF plot for the monthly data has limited partial autocorrelations that are significant. This suggests AR(0−2) for the monthly ARIMA model.

[Click here for more information about selected parameters for ARIMA models…](https://rpubs.com/riazakhan94/arima_with_example)

[…and here.](https://support.minitab.com/en-us/minitab/18/help-and-how-to/modeling-statistics/time-series/how-to/partial-autocorrelation/interpret-the-results/partial-autocorrelation-function-pacf/)

For seasonality, there are spikes in the weekly ACF at approximately 2 years, indicating MA(1−3). There are 3 spikes around 6 months in the PACF, indicating AR(1−3).

For seasonality in the monthly data, there are consistent spikes at 6 months, suggesting MA(2); and limited spikes in the PACF, suggesting AR(0−1).

## Fit an ARIMA model

An ARIMA model with a seasonal component can be called a SARIMA model.

Based on exploratory analysis, we expect the structure to be:

Weekly ARIMA: (2, 1, 1) (1−3, 0, 1−3) [52]

Monthly ARIMA: (0−2, 1, 0−2) (0−1, 0, 2) [12]

Initially we use an automated function auto.arima to determine the model structure. This will run very slowly if all models are tested. In the current study, the models were initially run with stepwise = T, and the same models were defined as when auto.arima was run without this argument. Therefore, we exclude stepwise = T from this code simply to increase computational speed.

We then fit other plausible models based on the exploratory analysis. We select the final model based on lowest AIC.

Auto_Arima = auto.arima(Parvo_ts_data)
summary(Auto_Arima)

## Series: Parvo_ts_data
## ARIMA(3,1,1)(1,0,0)[52] with drift
##
## Coefficients:

## Warning in sqrt(diag(x$var.coef)): NaNs produced

## ar1 ar2 ar3 ma1 sar1 drift
## -0.2209 -0.0671 -0.0155 -0.28 0.1117 -0.0926
## s.e. 0.0015 NaN 0.0008 NaN NaN 0.1475
##
## sigma^2 estimated as 18.9: log likelihood=-909.94
## AIC=1833.87 AICc=1834.24 BIC=1860.16
##
## Training set error measures:
## ME RMSE MAE MPE MAPE MASE ACF1
## Training set 0.1599227 4.299481 3.303407 -Inf Inf 0.5512612 -0.004982498

plot(forecast(Auto_Arima, 100))


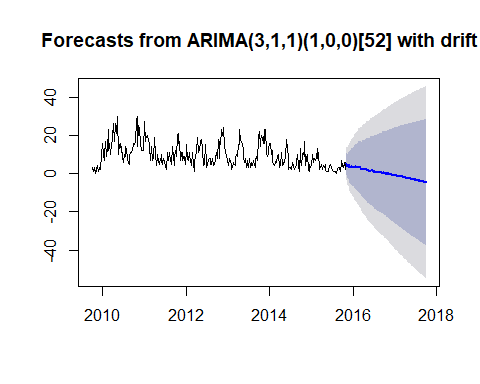


confint(Auto_Arima)

## Warning in sqrt(diag(vcov(object))): NaNs produced

## 2.5 % 97.5 %
## ar1 -0.22377041 -0.21806631
## ar2 NaN NaN
## ar3 -0.01703369 -0.01401343
## ma1 NaN NaN
## sar1 NaN NaN
## drift -0.38169498 0.19639837

checkresiduals(Auto_Arima)


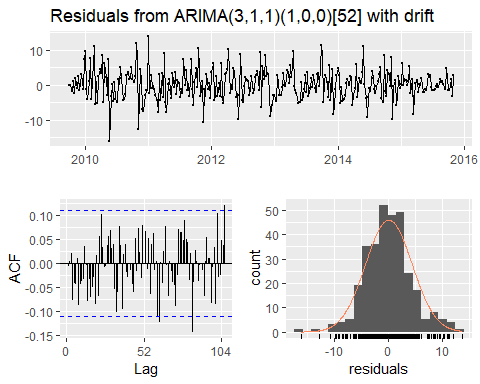


##
## Ljung-Box test
##
## data: Residuals from ARIMA(3,1,1)(1,0,0)[52] with drift
## Q* = 65.322, df = 57, p-value = 0.2101
##
## Model df: 6. Total lags used: 63

The automated function has selected a model with structure ARIMA(3,1,1)(1,0,0)[52] with drift. The drift term creates a trend in the forecast time-series.

Auto_ArimaM = auto.arima(Parvo_ts_dataM)
summary(Auto_ArimaM)

## Series: Parvo_ts_dataM
## ARIMA(2,1,1)(2,0,0)[12]
##
## Coefficients:
## ar1 ar2 ma1 sar1 sar2
## 0.7976 -0.2931 -0.9028 0.1562 0.2939
## s.e. 0.1445 0.1349 0.1120 0.1241 0.1361
##
## sigma^2 estimated as 289.2: log likelihood=-309.67
## AIC=631.33 AICc=632.61 BIC=645.08
##
## Training set error measures:
## ME RMSE MAE MPE MAPE MASE
## Training set -0.8649256 16.30082 13.35613 -43.75883 66.48852 0.6088825
## ACF1
## Training set -0.016731

plot(forecast(Auto_ArimaM,24))


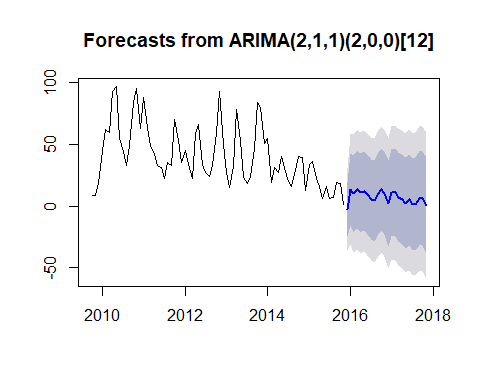


confint(Auto_ArimaM)

## 2.5 % 97.5 %
## ar1 0.51445880 1.08072576
## ar2 -0.55744923 -0.02881166
## ma1 -1.12243907 -0.68326091
## sar1 -0.08702874 0.39948777
## sar2 0.02719195 0.56070413

checkresiduals(Auto_ArimaM)


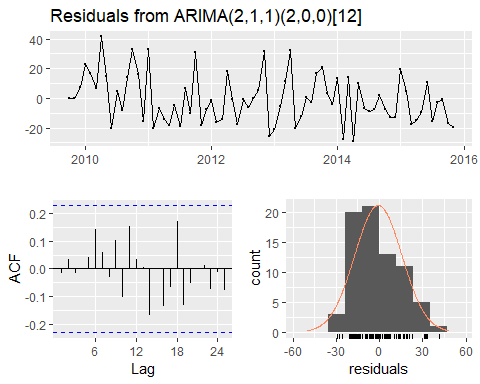


##
## Ljung-Box test
##
## data: Residuals from ARIMA(2,1,1)(2,0,0)[12]
## Q* = 9.0685, df = 10, p-value = 0.5256
##
## Model df: 5. Total lags used: 15

The automated functions suggest:

Weekly ARIMA (3,1,1)(1,0,0)[52] with drift, AICc=1834.24

Monthly ARIMA (2,1,1)(2,0,0)[12], AICc=632.61

We test other, simpler model structures which are still within the estimated parameters from time series exploration:

### Weekly time series models
## Weekly ARIMA: (2, 1, 1) (1-3, 0, 1-3) [52]
## fitted: (3,1,1)(1,0,0)[52] with drift, AICc=1834.24

fit1 = Arima(Parvo_ts_data, order = c(2, 1, 1), seasonal = c(1, 0, 0))
summary(fit1)

## Series: Parvo_ts_data
## ARIMA(2,1,1)(1,0,0)[52]
##
## Coefficients:
## ar1 ar2 ma1 sar1
## 0.4620 0.3052 -0.9855 0.1169
## s.e. 0.0559 0.0558 0.0133 0.0603
##
## sigma^2 estimated as 18.08: log likelihood=-904.79
## AIC=1819.58 AICc=1819.77 BIC=1838.36
##
## Training set error measures:
## ME RMSE MAE MPE MAPE MASE ACF1
## Training set -0.07217848 4.217939 3.29608 -Inf Inf 0.5500386 -0.03497213

plot(forecast(fit1,100))


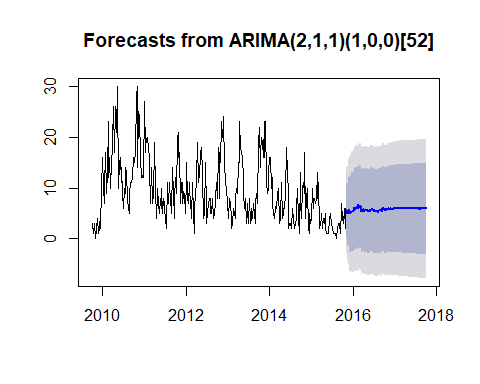


confint(fit1)

## 2.5 % 97.5 %
## ar1 0.352490442 0.5715407
## ar2 0.195782287 0.4145399
## ma1 -1.011501759 -0.9594912
## sar1 -0.001165745 0.2350279

checkresiduals(fit1)


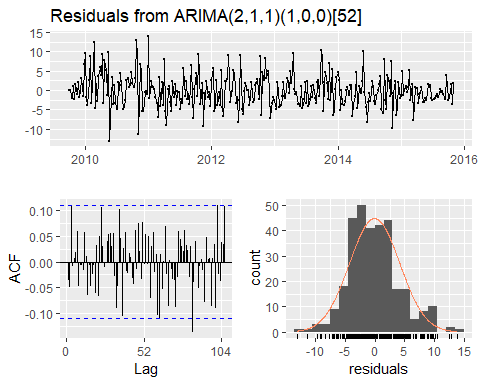


##
## Ljung-Box test
##
## data: Residuals from ARIMA(2,1,1)(1,0,0)[52]
## Q* = 60.566, df = 59, p-value = 0.4191
##
## Model df: 4. Total lags used: 63

fit2 = Arima(Parvo_ts_data, order = c(2, 1, 1), seasonal = c(0, 0, 1))
summary(fit2)

## Series: Parvo_ts_data
## ARIMA(2,1,1)(0,0,1)[52]
##
## Coefficients:
## ar1 ar2 ma1 sma1
## -0.3206 -0.1059 -0.1822 0.1082
## s.e. 0.6803 0.3097 0.6928 0.0586
##
## sigma^2 estimated as 18.78: log likelihood=-910.18
## AIC=1830.36 AICc=1830.55 BIC=1849.14
##
## Training set error measures:
## ME RMSE MAE MPE MAPE MASE ACF1
## Training set 0.00654623 4.299126 3.302973 -Inf Inf 0.5511889 -0.000116437

plot(forecast(fit2,100))


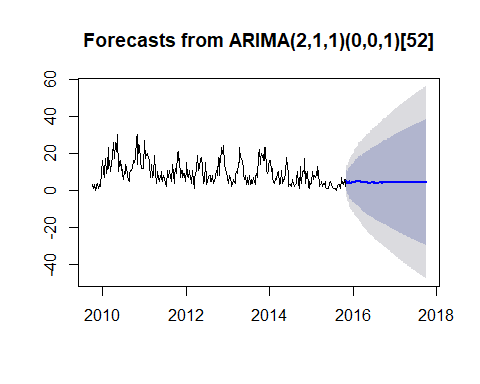


confint(fit2)

## 2.5 % 97.5 %
## ar1 -1.654048780 1.0127577
## ar2 -0.712904310 0.5010499
## ma1 -1.540119325 1.1757347
## sma1 -0.006698454 0.2230720

checkresiduals(fit2)


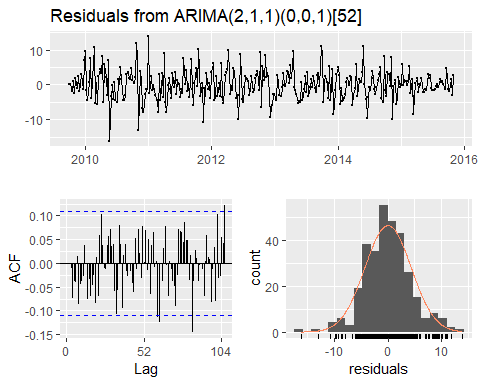


##
## Ljung-Box test
##
## data: Residuals from ARIMA(2,1,1)(0,0,1)[52]
## Q* = 65.577, df = 59, p-value = 0.2594
##
## Model df: 4. Total lags used: 63

fit3 = Arima(Parvo_ts_data, order = c(2, 1, 1), seasonal = c(1, 0, 0), include.drift = T) # *** This model is the best weekly model
summary(fit3)

## Series: Parvo_ts_data
## ARIMA(2,1,1)(1,0,0)[52] with drift
##
## Coefficients:
## ar1 ar2 ma1 sar1 drift
## 0.4620 0.3023 -1.0000 0.1123 -0.0228
## s.e. 0.0271 0.0347 0.0095 0.0380 0.0111
##
## sigma^2 estimated as 17.89: log likelihood=-903.6
## AIC=1819.2 AICc=1819.47 BIC=1841.73
##
## Training set error measures:
## ME RMSE MAE MPE MAPE MASE ACF1
## Training set 0.239633 4.188925 3.252542 -Inf Inf 0.542773 -0.03663276

plot(forecast(fit3,100))


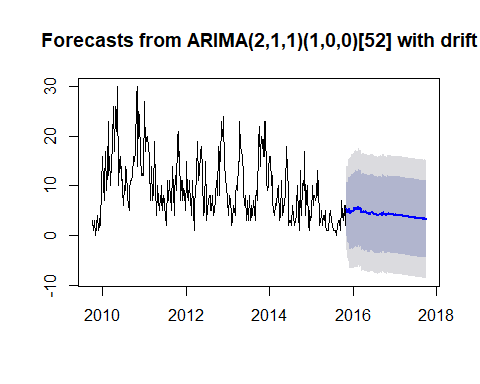


confint(fit3)

## 2.5 % 97.5 %
## ar1 0.40892020 0.5151448697
## ar2 0.23420620 0.3704037654
## ma1 -1.01863686 -0.9813614681
## sar1 0.03777440 0.1869119301
## drift -0.04465446 -0.0009709036

checkresiduals(fit3)


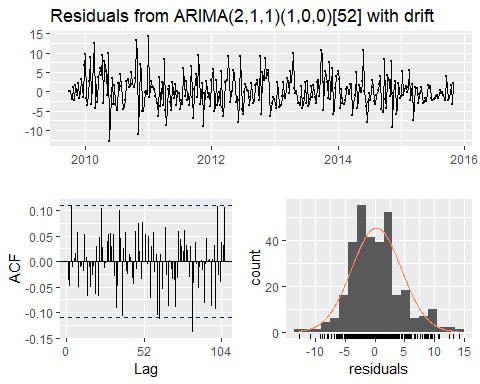


##
## Ljung-Box test
##
## data: Residuals from ARIMA(2,1,1)(1,0,0)[52] with drift
## Q* = 60.48, df = 58, p-value = 0.3864
##
## Model df: 5. Total lags used: 63

### monthly time series models
## Monthly ARIMA: (0-2, 1, 0-2) (0-1, 0, 2) [12]
## Auto fitted: (2,1,1)(2,0,0)[12], AICc=632.61

fit4 = Arima(Parvo_ts_dataM, order = c(1, 1, 1), seasonal = c(1, 0, 0))
summary(fit4)

## Series: Parvo_ts_dataM
## ARIMA(1,1,1)(1,0,0)[12]
##
## Coefficients:
## ar1 ma1 sar1
## 0.6992 -0.9741 0.3425
## s.e. 0.1237 0.0648 0.1195
##
## sigma^2 estimated as 315.6: log likelihood=-313.24
## AIC=634.47 AICc=635.06 BIC=643.63
##
## Training set error measures:
## ME RMSE MAE MPE MAPE MASE
## Training set -0.478025 17.27936 14.34423 -48.01381 72.26332 0.6539282
## ACF1
## Training set 0.10414

plot(forecast(fit4, 24))


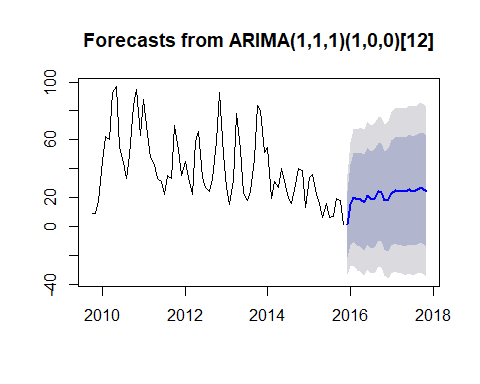


confint(fit4)

## 2.5 % 97.5 %
## ar1 0.4567312 0.9417214
## ma1 -1.1012459 -0.8470436
## sar1 0.1083085 0.5766908

checkresiduals(fit4)


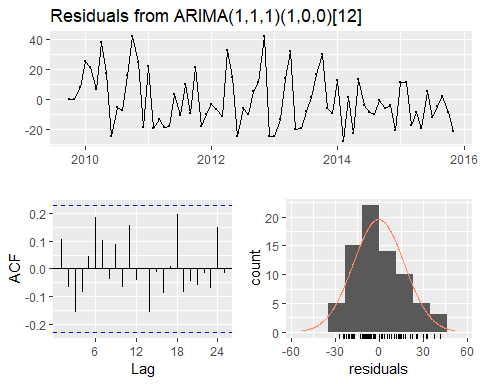


##
## Ljung-Box test
##
## data: Residuals from ARIMA(1,1,1)(1,0,0)[12]
## Q* = 13.434, df = 12, p-value = 0.3383
##
## Model df: 3. Total lags used: 15

fit5 = Arima(Parvo_ts_dataM, order = c(2, 1, 1), seasonal = c(0, 0, 1))
summary(fit5)

## Series: Parvo_ts_dataM
## ARIMA(2,1,1)(0,0,1)[12]
##
## Coefficients:
## ar1 ar2 ma1 sma1
## 0.7398 -0.2685 -0.9041 0.1761
## s.e. 0.1297 0.1299 0.0853 0.1156
##
## sigma^2 estimated as 317.3: log likelihood=-312.43
## AIC=634.87 AICc=635.77 BIC=646.32
##
## Training set error measures:
## ME RMSE MAE MPE MAPE MASE
## Training set -0.6784402 17.20082 13.94435 -46.66232 69.09401 0.6356984
## ACF1
## Training set -0.01497955

plot(forecast(fit5, 24))


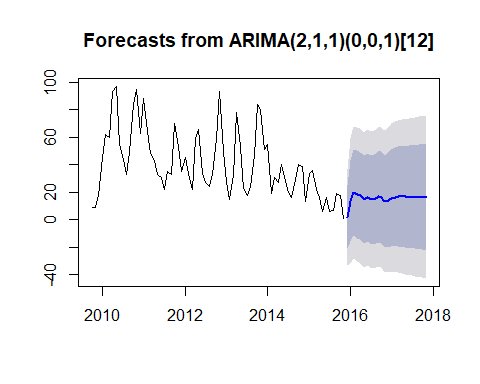


confint(fit5)

## 2.5 % 97.5 %
## ar1 0.48560490 0.99392851
## ar2 -0.52298029 -0.01392542
## ma1 -1.07138134 -0.73683305
## sma1 -0.05048666 0.40260207

checkresiduals(fit5)


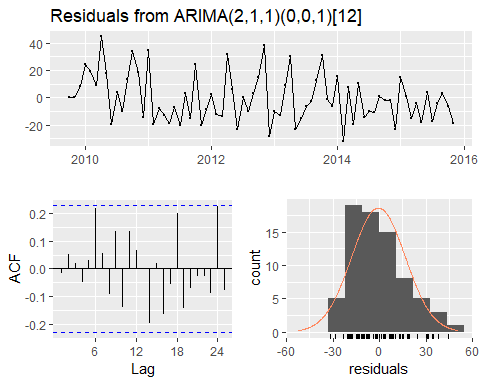


##
## Ljung-Box test
##
## data: Residuals from ARIMA(2,1,1)(0,0,1)[12]
## Q* = 14.416, df = 11, p-value = 0.2108
##
## Model df: 4. Total lags used: 15

fit6 = Arima(Parvo_ts_dataM, order = c(2, 1, 1), seasonal = c(2, 0, 0), include.drift = T)
summary(fit6)

## Series: Parvo_ts_dataM
## ARIMA(2,1,1)(2,0,0)[12] with drift
##
## Coefficients:
## ar1 ar2 ma1 sar1 sar2 drift
## 0.8313 -0.2963 -1.0000 0.1516 0.2841 -0.5061
## s.e. 0.1186 0.1278 0.0381 0.1222 0.1353 0.2061
##
## sigma^2 estimated as 279.2: log likelihood=-308.64
## AIC=631.29 AICc=633.01 BIC=647.32
##
## Training set error measures:
## ME RMSE MAE MPE MAPE MASE
## Training set 1.927427 15.89803 12.60622 -36.89273 64.13716 0.5746953
## ACF1
## Training set -0.02974478

plot(forecast(fit6, 24))


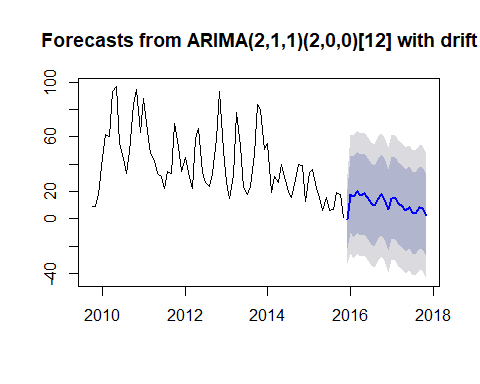


confint(fit6)

## 2.5 % 97.5 %
## ar1 0.59891581 1.06376762
## ar2 -0.54670833 -0.04585074
## ma1 -1.07463500 -0.92535175
## sar1 -0.08791284 0.39101991
## sar2 0.01888095 0.54933999
## drift -0.91008622 -0.10210976

checkresiduals(fit6)


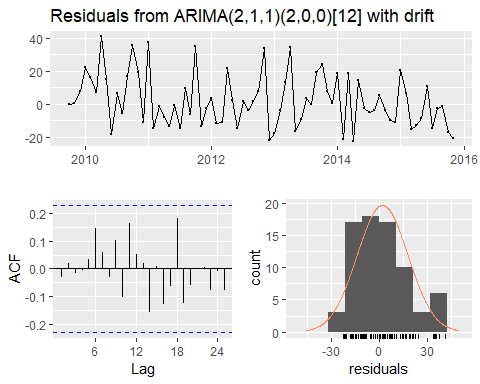


##
## Ljung-Box test
##
## data: Residuals from ARIMA(2,1,1)(2,0,0)[12] with drift
## Q* = 9.2198, df = 9, p-value = 0.4172
##
## Model df: 6. Total lags used: 15

fit7 = Arima(Parvo_ts_dataM, order = c(1, 1, 0), seasonal = c(1, 0, 0))
summary(fit7)

## Series: Parvo_ts_dataM
## ARIMA(1,1,0)(1,0,0)[12]
##
## Coefficients:
## ar1 sar1
## -0.0276 0.3670
## s.e. 0.1184 0.1136
##
## sigma^2 estimated as 350.6: log likelihood=-317.31
## AIC=640.63 AICc=640.97 BIC=647.5
##
## Training set error measures:
## ME RMSE MAE MPE MAPE MASE
## Training set -0.3311234 18.34056 14.86254 -33.96177 64.98723 0.677557
## ACF1
## Training set -0.003681255

plot(forecast(fit7, 24))


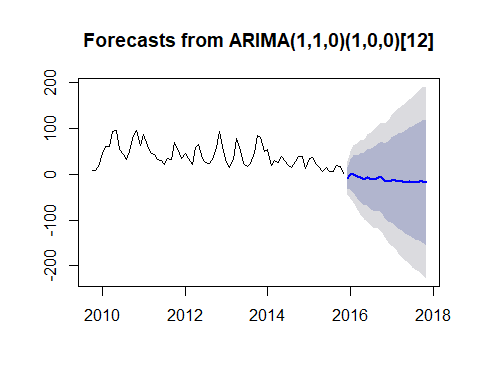


confint(fit7)

## 2.5 % 97.5 %
## ar1 -0.2596615 0.2044482
## sar1 0.1443652 0.5895377

checkresiduals(fit7)


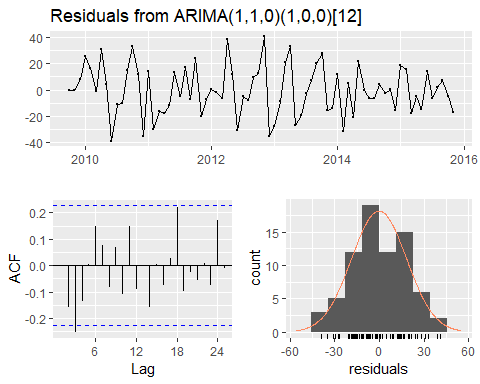


##
## Ljung-Box test
##
## data: Residuals from ARIMA(1,1,0)(1,0,0)[12]
## Q* = 17.913, df = 13, p-value = 0.1609
##
## Model df: 2. Total lags used: 15

The final models are ‘fit3’ for the weekly time-series and the auto-fitted model for the monthly time series. Each has the lowest AICc of tested models, and produces a plausible forecast of the original time series. The residuals are reasonably Normally distributed and the ACF plot of the residuals and Ljung-Box tests suggest that the residuals are time independent.

# Multivariate forecasting with time-series

In this section, we first consider how to modify an ARIMA model to include a predictor (other than the univariate time-series). We use the example of rainfall as a predictor of parvovirus events, and select monthly rainfall at Mudgee, NSW (mean centre of parvovirus event data).

1. Prepare, describe and decompose the rainfall series

RainfallM = read.csv('D:/Users/vbrookes/Dropbox (Sydney Uni)/Parvo_timeseries/Mudgee rainfall/Mudgee_Monthly_rainfall.csv')
summary(RainfallM)

## Date Rain_mm
## 1/01/2010: 1 Min. : 5.40
## 1/01/2011: 1 1st Qu.: 24.80
## 1/01/2012: 1 Median : 47.50
## 1/01/2013: 1 Mean : 56.45
## 1/01/2014: 1 3rd Qu.: 75.40
## 1/01/2015: 1 Max. : 214.40
## (Other) :68

str(RainfallM)

## 'data.frame': 74 obs. of 2 variables:
## $ Date : Factor w/ 74 levels "1/01/2010","1/01/2011",..: 55 62 69 1 7 13 19 25 31 37 ...
## $ Rain_mm: num 34 16 121.4 46.4 90 ...

head(RainfallM)

## Date Rain_mm
## 1 1/10/2009 34.0
## 2 1/11/2009 16.0
## 3 1/12/2009 121.4
## 4 1/01/2010 46.4
## 5 1/02/2010 90.0
## 6 1/03/2010 89.0

tail(RainfallM)

## Date Rain_mm
## 69 1/06/2015 55.20
## 70 1/07/2015 61.60
## 71 1/08/2015 30.40
## 72 1/09/2015 5.40
## 73 1/10/2015 44.00
## 74 1/11/2015 101.45

RainfallM$Date = as.character(RainfallM$Date, format = "%d/%m/%Y")
RainfallM$Date = as.Date(RainfallM$Date, "%d/%m/%Y")

RainfallM$byMonth = cut(RainfallM$Date, breaks="1 month")
RainfallM$byMonth <- as.Date(RainfallM$byMonth, format = "%Y-%m-%d")

# summary
summary(RainfallM)

## Date Rain_mm byMonth
## Min. : 2009-10-01 Min. : 5.40 Min. : 2009-10-01
## 1st Qu.: 2011-04-08 1st Qu.: 24.80 1st Qu.: 2011-04-08
## Median : 2012-10-16 Median : 47.50 Median : 2012-10-16
## Mean : 2012-10-15 Mean : 56.45 Mean : 2012-10-15
## 3rd Qu.: 2014-04-23 3rd Qu.: 75.40 3rd Qu.: 2014-04-23
## Max. : 2015-11-01 Max. : 214.40 Max. : 2015-11-01

# check structure
str(RainfallM)

## 'data.frame': 74 obs. of 3 variables:
## $ Date : Date, format: "2009-10-01" "2009-11-01" ...
## $ Rain_mm: num 34 16 121.4 46.4 90 ...
## $ byMonth: Date, format: "2009-10-01" "2009-11-01" ...

# Check length
length(RainfallM$Dates)

## [1] 0

ggplot(RainfallM, aes(x = byMonth, y = Rain_mm, group = 1)) +
 geom_bar(stat="identity", width = 0.5, colour = "black") +
 stat_smooth(aes(y = Rain_mm), method='auto', level=0.95) +
 themeVB +
 scale_x_date() +
 xlab("Year") +
 ylab("Rainfall")

## `geom_smooth()` using method = 'loess' and formula 'y ~ x'


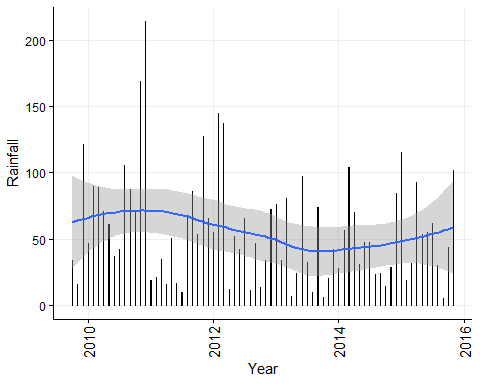


Rainfall_ts_data <- ts(RainfallM$Rain_mm, start = c(2009, 10), frequency =12)

Decompose_tsRainM <- decompose(Rainfall_ts_data)
summary(Decompose_tsRainM)

## Length Class Mode
## x 74 ts numeric
## seasonal 74 ts numeric
## trend 74 ts numeric
## random 74 ts numeric
## figure 12 -none- numeric
## type 1 -none- character

plot(Decompose_tsRainM)


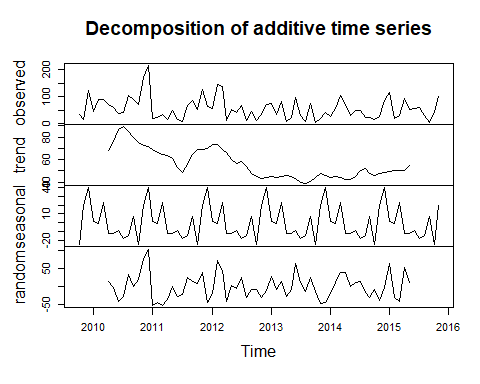


Quantitatively assess the trend, seasonality and stationarity of the data.

# Assess trend and seasonality
fit_Rain_dataM = lm(Rainfall_ts_data ~ time(Rainfall_ts_data) + factor(cycle(Rainfall_ts_data)))
summary(fit_Rain_dataM)

##
## Call:
## lm(formula = Rainfall_ts_data ~ time(Rainfall_ts_data) + factor(cycle(Rainfall_ts_data)))
##
## Residuals:
## Min 1Q Median 3Q Max
## -66.87 -28.45 -4.26 21.10 108.41
##
## Coefficients:
## Estimate Std. Error t value Pr(>|t|)
## (Intercept) 8079.93470 5220.28469 1.548 0.1268
## time(Rainfall_ts_data) -3.98680 2.59392 -1.537 0.1295
## factor(cycle(Rainfall_ts_data))2 4.79890 22.76256 0.211 0.8337
## factor(cycle(Rainfall_ts_data))3 23.69447 22.76564 1.041 0.3021
## factor(cycle(Rainfall_ts_data))4 -10.80997 22.77077 -0.475 0.6367
## factor(cycle(Rainfall_ts_data))5 -10.11107 22.77795 -0.444 0.6587
## factor(cycle(Rainfall_ts_data))6 -5.56883 22.78718 -0.244 0.8078
## factor(cycle(Rainfall_ts_data))7 -11.44660 22.79846 -0.502 0.6174
## factor(cycle(Rainfall_ts_data))8 -13.04770 22.81178 -0.572 0.5694
## factor(cycle(Rainfall_ts_data))9 0.08453 22.82713 0.004 0.9971
## factor(cycle(Rainfall_ts_data))10 -21.79568 21.94315 -0.993 0.3245
## factor(cycle(Rainfall_ts_data))11 15.72798 21.95060 0.717 0.4764
## factor(cycle(Rainfall_ts_data))12 43.16777 22.76256 1.896 0.0626 .
## ---
## Signif. codes: 0 '***' 0.001 '**' 0.01 '*' 0.05 '.' 0.1 ' ' 1
##
## Residual standard error: 39.42 on 61 degrees of freedom
## Multiple R-squared: 0.2273, Adjusted R-squared: 0.07531
## F-statistic: 1.495 on 12 and 61 DF, p-value: 0.1507

confint(fit_Rain_dataM)

## 2.5 % 97.5 %
## (Intercept) -2358.669697 18518.539091
## time(Rainfall_ts_data) -9.173656 1.200063
## factor(cycle(Rainfall_ts_data))2 -40.717660 50.315460
## factor(cycle(Rainfall_ts_data))3 -21.828250 69.217183
## factor(cycle(Rainfall_ts_data))4 -56.342943 34.723008
## factor(cycle(Rainfall_ts_data))5 -55.658402 35.436267
## factor(cycle(Rainfall_ts_data))6 -51.134624 39.996954
## factor(cycle(Rainfall_ts_data))7 -57.034937 34.141733
## factor(cycle(Rainfall_ts_data))8 -58.662667 32.567263
## factor(cycle(Rainfall_ts_data))9 -45.561143 45.730205
## factor(cycle(Rainfall_ts_data))10 -65.673724 22.082360
## factor(cycle(Rainfall_ts_data))11 -28.164962 59.620922
## factor(cycle(Rainfall_ts_data))12 -2.348793 88.684327

# Tests for stationarity
Rainfall_ts_data %>% ggtsdisplay(theme = themeVB)


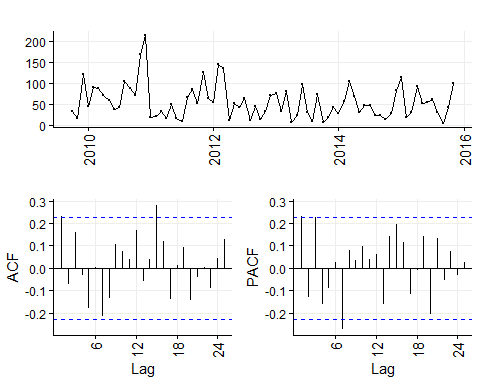


# We run `ndiffs` and `nsdiffs` (seasonal) from the `forecast` package to assess how many first differences are needed to induce stationarity.
ndiffs(Rainfall_ts_data)

## [1] 0

nsdiffs(Rainfall_ts_data)

## [1] 0

## Monthly time-series tests for stationarity
# 1.
Box.test(Rainfall_ts_data, lag = 12, type="Ljung-Box")

##
## Box-Ljung test
##
## data: Rainfall_ts_data
## X-squared = 18.627, df = 12, p-value = 0.09794

# 2.
adf.test(Rainfall_ts_data, alternative = "stationary", k = 24)

## Warning in adf.test(Rainfall_ts_data, alternative = "stationary", k = 24):
## p-value greater than printed p-value

##
## Augmented Dickey-Fuller Test
##
## data: Rainfall_ts_data
## Dickey-Fuller = -0.071898, Lag order = 24, p-value = 0.99
## alternative hypothesis: stationary

# 3.
kpss.test(Rainfall_ts_data, null = 'Trend')

## Warning in kpss.test(Rainfall_ts_data, null = "Trend"): p-value greater
## than printed p-value

##
## KPSS Test for Trend Stationarity
##
## data: Rainfall_ts_data
## KPSS Trend = 0.052388, Truncation lag parameter = 3, p-value = 0.1

kpss.test(Rainfall_ts_data, null = 'Level')

## Warning in kpss.test(Rainfall_ts_data, null = "Level"): p-value greater
## than printed p-value

##
## KPSS Test for Level Stationarity
##
## data: Rainfall_ts_data
## KPSS Level = 0.30441, Truncation lag parameter = 3, p-value = 0.1

There is no quantitative evidence for trend and seasonality of monthly rainfall at Mudgee. Also, the raw time series appears to be stationary; significant correlations appear at lag 15, but not in the first few lags, the functions ‘ndiff’ and ‘nsdiff’ indicate that differencing is not required, and statistical tests indicate stationarity.

1. We can therefore, use the raw rainfall series in the model.

fit <- auto.arima(Parvo_ts_dataM, xreg=Rainfall_ts_data)
summary(fit)

## Series: Parvo_ts_dataM
## Regression with ARIMA(2,1,1)(2,0,0)[12] errors
##
## Coefficients:
## ar1 ar2 ma1 sar1 sar2 xreg
## 0.8769 -0.305 -0.9209 0.1032 0.3378 -0.1057
## s.e. 0.1300 0.131 0.0810 0.1204 0.1303 0.0393
##
## sigma^2 estimated as 266.7: log likelihood=-306.4
## AIC=626.81 AICc=628.53 BIC=642.84
##
## Training set error measures:
## ME RMSE MAE MPE MAPE MASE
## Training set -0.8473194 15.53964 12.91691 -37.30511 61.52266 0.5888593
## ACF1
## Training set -0.0273563

confint(fit)

## 2.5 % 97.5 %
## ar1 0.6221184 1.13175374
## ar2 -0.5617401 -0.04830879
## ma1 -1.0796567 -0.76214332
## sar1 -0.1328496 0.33927111
## sar2 0.0823451 0.59329433
## xreg -0.1827247 -0.02877087

checkresiduals(fit)


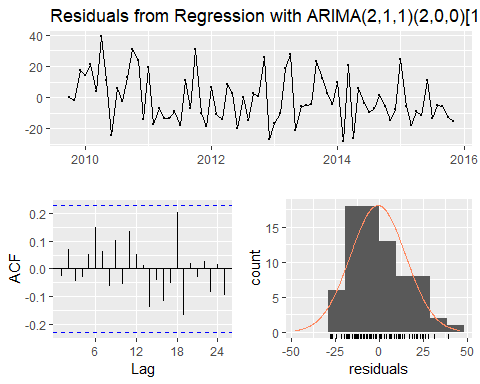


##
## Ljung-Box test
##
## data: Residuals from Regression with ARIMA(2,1,1)(2,0,0)[12] errors
## Q* = 8.5041, df = 9, p-value = 0.4842
##
## Model df: 6. Total lags used: 15

fcast <- forecast(fit, xreg=rep(mean(Rainfall_ts_data),24))

autoplot(fcast) + xlab("Year") + ylab("Events")


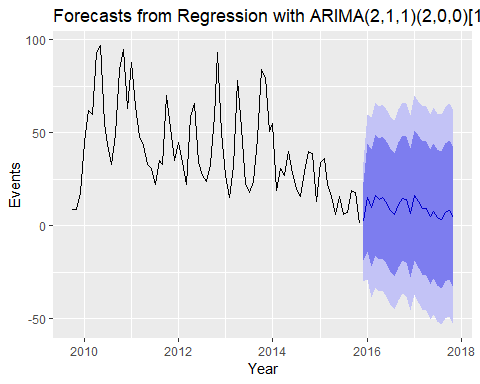


An increase in rainfall is associated with a decrease in parvo events, and vice versa. This model has a lower AICc (628.53) than the model without rainfall as a predictor (AICc = 632.61). The forecast plot results in a slightly narrower range of predictions over time than the model without rainfall as a predictor.

### Vector auto-regression (VAR) models

In these models there are no assumptions made about the direction of prediction, and the potential influence of one time-series on the other is symmetrical. Therefore, parvovirus events could predict rainfall, or rainfall could predict parvovirus events. Obviously, this is a ridiculous suggestion in this context, but it can be useful to give insights in other contexts. Examples could include exploration of the direction of disease spread between two populations (we want to explore causation). It is also useful for forecasting when we simply want a mathematically useful predictive model (we have made a prior causal model and are interested in predictions in one direction).

Initially we examine the correlation between lags of monthly parvovirus reports and rainfall using cross-correlation plots.

ccf(Parvo_ts_dataM, Rainfall_ts_data, type = 'correlation')


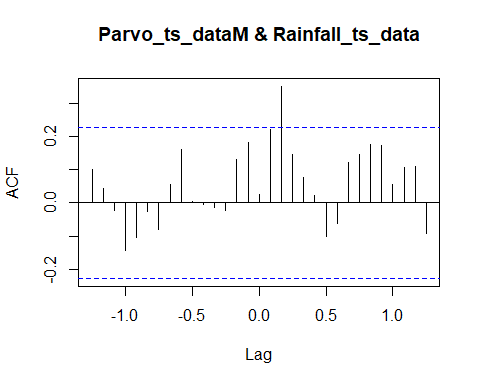


There appears to be cross-correlation between parvovirus events and rainfall at +2 months (rainfall leads parvovirus events on this side of the plot, which is also biologically sensible).

The model we are going to fit is a VAR model (no I or MA), so data need to be differenced to induce stationarity if necessary prior to model fitting. A predictive equation is fitted for each of parvovirus events and rainfall. The equations are symmetrical (same number of predictor variables and lags).

# Difference Parvo data because model is VARMA (no I)
ParvoM_diff = diff(Parvo_ts_dataM)

Differenced_data <- cbind(ParvoM_diff, Rainfall_ts_data) # Combine in a dataframe with the rainfall data.
Differenced_data[is.na(Differenced_data)] <- 0 # Convert first value from NA to 0

In our example, we have two variables (parvovirus events and rainfall, k = 2). We need to determine the number of AR lags (p) for each equation in the model. We use the VARselect function to estimate the p order in the model.

The output gives: 1. AIC − for VAR models, the BIC (SC) is preferable, because AIC tends to select large numbers of lags. 2. HQ − Hannan-Quinn criterion 3. SC − another name for the BIC (SC stands for Schwarz Criterion, after Gideon Schwarz who proposed it) and 4. FPE − “Final Prediction Error” criterion.

The number of coefficients estimated in a VAR = k+pk^2^. Note that because every variable is assumed to influence every other variable in the system, it makes a direct interpretation of the estimated coefficients difficult.

# estimate orders for AR(p) and MA(q) between parvo events and rainfall
library(vars)
VARselect(Differenced_data, lag.max = 12, type = "const") # AR(p) = 1 if use SC (BIC), or p = 4 if use the other information criteria.

## $selection
## AIC(n) HQ(n) SC(n) FPE(n)
## 4 4 1 4
##
## $criteria
## 1 2 3 4 5
## AIC(n) 13.41217 13.39258 13.30062 13.17956 13.25029
## HQ(n) 13.49299 13.52728 13.48920 13.42203 13.54664
## SC(n) 13.61802 13.73566 13.78094 13.79712 14.00508
## FPE(n) 668187.14652 655582.27185 598721.72745 531623.77958 572564.43269
## 6 7 8 9 10
## AIC(n) 13.35469 13.32362 13.3147 13.42150 13.36591
## HQ(n) 13.70492 13.72773 13.7727 13.93338 13.93167
## SC(n) 14.24671 14.35288 14.4812 14.72523 14.80688
## FPE(n) 638779.39943 623554.67441 623747.0729 702355.16589 674422.90712
## 11 12
## AIC(n) 13.48327 13.56223
## HQ(n) 14.10291 14.23575
## SC(n) 15.06146 15.27766
## FPE(n) 772645.69114 855383.04320

# Automated model fit
Mod1 <- VAR(Differenced_data, p=1, type = "const") # automated
Mod1

##
## VAR Estimation Results:
## =======================
##
## Estimated coefficients for equation ParvoM_diff:
## ================================================
## Call:
## ParvoM_diff = ParvoM_diff.l1 + Rainfall_ts_data.l1 + const
##
## ParvoM_diff.l1 Rainfall_ts_data.l1 const
## 0.06779822 0.10898642 -6.20340599
##
##
## Estimated coefficients for equation Rainfall_ts_data:
## =====================================================
## Call:
## Rainfall_ts_data = ParvoM_diff.l1 + Rainfall_ts_data.l1 + const
##
## ParvoM_diff.l1 Rainfall_ts_data.l1 const
## 0.1854748 0.2506427 42.7428547

summary(Mod1)

##
## VAR Estimation Results:
## =========================
## Endogenous variables: ParvoM_diff, Rainfall_ts_data
## Deterministic variables: const
## Sample size: 73
## Log Likelihood: -689.54
## Roots of the characteristic polynomial:
## 0.3283 0.009813
## Call:
## VAR(y = Differenced_data, p = 1, type = "const")
##
##
## Estimation results for equation ParvoM_diff:
## ============================================
## ParvoM_diff = ParvoM_diff.l1 + Rainfall_ts_data.l1 + const
##
## Estimate Std. Error t value Pr(>|t|)
## ParvoM_diff.l1 0.0678 0.1194 0.568 0.5720
## Rainfall_ts_data.l1 0.1090 0.0582 1.872 0.0653 .
## const -6.2034 3.9966 -1.552 0.1251
## ---
## Signif. codes: 0 '***' 0.001 '**' 0.01 '*' 0.05 '.' 0.1 ' ' 1
##
##
## Residual standard error: 19.84 on 70 degrees of freedom
## Multiple R-Squared: 0.04828, Adjusted R-squared: 0.02109
## F-statistic: 1.775 on 2 and 70 DF, p-value: 0.1769
##
##
## Estimation results for equation Rainfall_ts_data:
## =================================================
## Rainfall_ts_data = ParvoM_diff.l1 + Rainfall_ts_data.l1 + const
##
## Estimate Std. Error t value Pr(>|t|)
## ParvoM_diff.l1 0.1855 0.2436 0.761 0.4490
## Rainfall_ts_data.l1 0.2506 0.1187 2.111 0.0384 *
## const 42.7429 8.1534 5.242 1.6e-06 ***
## ---
## Signif. codes: 0 '***' 0.001 '**' 0.01 '*' 0.05 '.' 0.1 ' ' 1
##
##
## Residual standard error: 40.48 on 70 degrees of freedom
## Multiple R-Squared: 0.0615, Adjusted R-squared: 0.03468
## F-statistic: 2.293 on 2 and 70 DF, p-value: 0.1085
##
##
## Covariance matrix of residuals:
## ParvoM_diff Rainfall_ts_data
## ParvoM_diff 393.6 -218.7
## Rainfall_ts_data -218.7 1638.2
##
## Correlation matrix of residuals:
## ParvoM_diff Rainfall_ts_data
## ParvoM_diff 1.0000 -0.2724
## Rainfall_ts_data -0.2724 1.0000

serial.test(Mod1, lags.pt=12)

##
## Portmanteau Test (asymptotic)
##
## data: Residuals of VAR object Mod1
## Chi-squared = 60.176, df = 44, p-value = 0.05278

Mod2 <- VAR(Differenced_data, p=4, type = "const") # automated
Mod2

##
## VAR Estimation Results:
## =======================
##
## Estimated coefficients for equation ParvoM_diff:
## ================================================
## Call:
## ParvoM_diff = ParvoM_diff.l1 + Rainfall_ts_data.l1 + ParvoM_diff.l2 + Rainfall_ts_data.l2 + ParvoM_diff.l3 + Rainfall_ts_data.l3 + ParvoM_diff.l4 + Rainfall_ts_data.l4 + const
##
## ParvoM_diff.l1 Rainfall_ts_data.l1 ParvoM_diff.l2
## -0.12612139 0.08036881 -0.23760011
## Rainfall_ts_data.l2 ParvoM_diff.l3 Rainfall_ts_data.l3
## 0.13623172 -0.31163388 -0.11432214
## ParvoM_diff.l4 Rainfall_ts_data.l4 const
## -0.36440792 -0.01166325 -5.66543045
##
##
## Estimated coefficients for equation Rainfall_ts_data:
## =====================================================
## Call:
## Rainfall_ts_data = ParvoM_diff.l1 + Rainfall_ts_data.l1 + ParvoM_diff.l2 + Rainfall_ts_data.l2 + ParvoM_diff.l3 + Rainfall_ts_data.l3 + ParvoM_diff.l4 + Rainfall_ts_data.l4 + const
##
## ParvoM_diff.l1 Rainfall_ts_data.l1 ParvoM_diff.l2
## 0.25266461 0.38468136 0.32449908
## Rainfall_ts_data.l2 ParvoM_diff.l3 Rainfall_ts_data.l3
## -0.23646772 0.01717014 0.21493518
## ParvoM_diff.l4 Rainfall_ts_data.l4 const
## 0.18977775 -0.14593788 44.59590673

summary(Mod2)

##
## VAR Estimation Results:
## =========================
## Endogenous variables: ParvoM_diff, Rainfall_ts_data
## Deterministic variables: const
## Sample size: 70
## Log Likelihood: -643.678
## Roots of the characteristic polynomial:
## 0.8163 0.8163 0.7091 0.7091 0.643 0.643 0.6323 0.6323
## Call:
## VAR(y = Differenced_data, p = 4, type = "const")
##
##
## Estimation results for equation ParvoM_diff:
## ============================================
## ParvoM_diff = ParvoM_diff.l1 + Rainfall_ts_data.l1 + ParvoM_diff.l2 + Rainfall_ts_data.l2 + ParvoM_diff.l3 + Rainfall_ts_data.l3 + ParvoM_diff.l4 + Rainfall_ts_data.l4 + const
##
## Estimate Std. Error t value Pr(>|t|)
## ParvoM_diff.l1 -0.12612 0.12112 -1.041 0.30187
## Rainfall_ts_data.l1 0.08037 0.05744 1.399 0.16683
## ParvoM_diff.l2 -0.23760 0.11217 -2.118 0.03825 *
## Rainfall_ts_data.l2 0.13623 0.06052 2.251 0.02800 *
## ParvoM_diff.l3 -0.31163 0.11220 -2.778 0.00727 **
## Rainfall_ts_data.l3 -0.11432 0.05887 -1.942 0.05675 .
## ParvoM_diff.l4 -0.36441 0.11621 -3.136 0.00264 **
## Rainfall_ts_data.l4 -0.01166 0.05815 -0.201 0.84171
## const -5.66543 5.34005 -1.061 0.29290
## ---
## Signif. codes: 0 '***' 0.001 '**' 0.01 '*' 0.05 '.' 0.1 ' ' 1
##
##
## Residual standard error: 17.31 on 61 degrees of freedom
## Multiple R-Squared: 0.3499, Adjusted R-squared: 0.2646
## F-statistic: 4.104 on 8 and 61 DF, p-value: 0.00058
##
##
## Estimation results for equation Rainfall_ts_data:
## =================================================
## Rainfall_ts_data = ParvoM_diff.l1 + Rainfall_ts_data.l1 + ParvoM_diff.l2 + Rainfall_ts_data.l2 + ParvoM_diff.l3 + Rainfall_ts_data.l3 + ParvoM_diff.l4 + Rainfall_ts_data.l4 + const
##
## Estimate Std. Error t value Pr(>|t|)
## ParvoM_diff.l1 0.25266 0.27317 0.925 0.35864
## Rainfall_ts_data.l1 0.38468 0.12955 2.969 0.00426 **
## ParvoM_diff.l2 0.32450 0.25298 1.283 0.20445
## Rainfall_ts_data.l2 -0.23647 0.13649 -1.732 0.08825 .
## ParvoM_diff.l3 0.01717 0.25304 0.068 0.94612
## Rainfall_ts_data.l3 0.21494 0.13276 1.619 0.11060
## ParvoM_diff.l4 0.18978 0.26209 0.724 0.47178
## Rainfall_ts_data.l4 -0.14594 0.13115 -1.113 0.27018
## const 44.59591 12.04326 3.703 0.00046 ***
## ---
## Signif. codes: 0 '***' 0.001 '**' 0.01 '*' 0.05 '.' 0.1 ' ' 1
##
##
## Residual standard error: 39.04 on 61 degrees of freedom
## Multiple R-Squared: 0.2003, Adjusted R-squared: 0.09537
## F-statistic: 1.909 on 8 and 61 DF, p-value: 0.07474
##
##
## Covariance matrix of residuals:
## ParvoM_diff Rainfall_ts_data
## ParvoM_diff 299.6 -135.9
## Rainfall_ts_data -135.9 1524.0
##
## Correlation matrix of residuals:
## ParvoM_diff Rainfall_ts_data
## ParvoM_diff 1.0000 -0.2012
## Rainfall_ts_data -0.2012 1.0000

serial.test(Mod2, lags.pt=12)

##
## Portmanteau Test (asymptotic)
##
## data: Residuals of VAR object Mod2
## Chi-squared = 29.716, df = 32, p-value = 0.5826

# we plot forecasts from each predictive equation
forecast(Mod1) %>%
 autoplot() + xlab("Year")


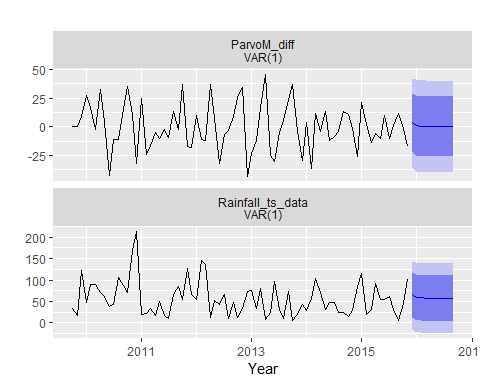


forecast(Mod2) %>%
 autoplot() + xlab("Year")


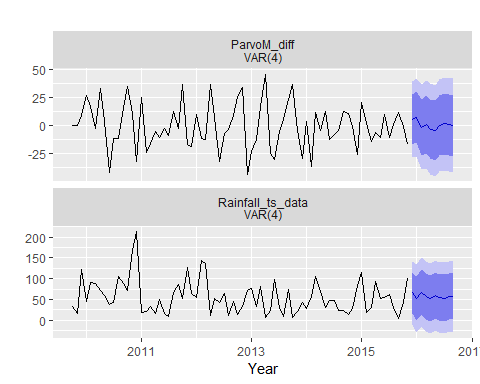


In the model with p = 1, there are three coefficients estimated for each equation (1 lag each for rainfall and parvovirus events as predictors of parvovirus events [and rainfall events], and 1 constant). In this model, none of the coefficients for the parvovirus events equation are significantly predictive and in the Portmanteau test, P = 0.05 (rejecting the null hypothesis of no correlation between residuals).

In the model with p = 4, there are 18 coefficients (9 for each equation). There are significant predictors of parvovirus events (previous parvovirus events and rainfall at lags 2, 3 and 4). In the Portmanteau test, P = 0.58 (consistent with the null hypothesis of no correlation between residuals).

We would select the model with p = 4 as more useful for prediction of parvovirus events. In this case, we have no interest in prediction of rainfall, but this predictive equation would also be of interest if a plausible causal relationship was agreed *a priori*.

For further information about vector auto-regressive models and other extensions of time-series analysis and forecasting, we recommend:

Hyndman, R.J., & Athanasopoulos, G. (2018) Forecasting: principles and practice, 2nd edition, OTexts: Melbourne, Australia. OTexts.com/fpp2. Accessed on 24/10/2019.

An online copy of this book can be found [here](https://otexts.com/fpp2/).
